# Defining the preadmission factors that modify risk of acute complications, survival and long-term recovery from COVID-19: prospective, longitudinal, multisite, cohort study based in England

**DOI:** 10.1136/bmjopen-2025-109653

**Published:** 2026-09-21

**Authors:** H E Baxendale, Martin Law, Claire Matthews, Janet Piggott, Rama Vancheeswaran, Alain Vuylsteke, Catherine Wilson, Erin Hopley, Mark Toshner, Iryna Boubriak, Joseph Newman, Nasir Hannan, Alexander JK Wilkinson, Andrew Barlow, Nicole Harriott, Nicola Jones, Stephen Webb, William Schwaeble, Jonathan Heeney, Dominique Couturier

**Affiliations:** 1 Royal Papworth Hospital, Cambridge, England, UK; 2 University of Cambridge, Cambridge, England, UK; 3 Royal Papworth Hospital, Papworth Trials Unit Collaboration, Cambridge, England, UK; 4 University of Cambridge, MRC Biostatistics Unit, Cambridge, England, UK; 5 NIHR LCRN Eastern Core Team, London, England, UK; 6 West Hertfordshire Teaching Hospitals NHS Trust, Respiratory Medicine Watford, Cambridge, England, UK; 7 Victor Phillip Dahdaleh Heart & Lung Research Institute, University of Cambridge, Cambridge, UK; 8 Priory Gardens Surgery, Luton and Dunstable Community Healthcare Trust. Dunstable, Cambridge, England, UK; 9 Respiratory Department, East and North Hertfordshire NHS Trust, Stevenage, UK; 10 Department of Veterinary Medicine, University of Cambridge, Cambridge, UK

**Keywords:** COVID-19, Post-Acute COVID-19 Syndrome, EPIDEMIOLOGY, Adult intensive & critical care

## Abstract

**Abstract:**

**Objectives:**

To understand the pre-admission factors associated with COVID-19 clinical outcome with and without controlling for acute COVID-19 severity score.

**Design:**

We ran a prospective, longitudinal, multicentre cohort study.

**Setting:**

Patients were recruited from four hospitals and one community General Practice in England

**Participants:**

425 patients with COVID-19 from the start of the UK pandemic in April 2020 through the first three waves with study completing in 2022.

**Primary and secondary outcome measures:**

Data were collected and analysed to identify demographic factors and preadmission symptoms for hospitalised patients that were associated with COVID-19 disease severity at recruitment, acute clinical course and long-term recovery. Analysis methods included logistic regression, time-to-event analysis, mixed-effect models and K-medoids clustering.

**Results:**

The cohort was skewed to patients with severe COVID (60%). Preadmission symptom cluster was associated with risk of thrombosis (OR 2.7 (95% CI 1.6 to 6.1), p=0.021) and renal disease (OR 3.3 (95% CI 1.4 to 8.0), p=0.008). Duration of symptoms less than 1 week was associated with pneumothorax (OR 3.1 (95% CI 1.2 to 8.3), p=0.025). Renal complications were more likely to be seen in the first wave compared with the second wave of the pandemic (OR 3.4 (95% CI 1.5 to 7.5), p=0.003). Using a logistic regression model, survival to discharge was associated with white (vs unknown) ethnicity (OR 6.6 (95% CI 2.8 to 15.2), p<0.0001), lower age (<40 years vs >60 years, OR 4.9 (95% CI 1.2 to 20.0), p=0.027) and absence of comorbidities (OR 2.7 (95% CI 1.2 to 5.9), p=0.016). However, using a survival model, hazard of death was increased for all hospitalised who had duration of symptoms less than 1 week (HR 5.6 (95% CI 1.3 to 24.4), p=0.023) or had comorbidities (HR 33.3 (95% CI 3.1 to 333.3), p=0.0035). Over 80% of patients reported long-term sequelae with neuromuscular and cognitive effects dominating early on and mood disturbance and breathlessness persisting to 12 months. Gender had the strongest association with dyspnoea (OR 99.8 (95% CI 2.4 to 4123.1, p=0.02) and persistent mood disorder (OR 8.3 (95% CI 1.7 to 39.9), p=0.008) and associated with persistent gastrointestinal problems (loose bowels (OR 3.3 (95% CI 1.1 to 10.8), p=0.045), abdominal pain (OR 5.0 (95% CI 1.4 to 18.1), p=0.014), constipation (OR 3.8 (95% CI 1.6 to 9.2), p=0.003)) and cognitive problems (OR 2.3 (95% CI 1.1 to −4.8), p=0.024).

**Conclusions:**

These results support an association between specific demographic factors and early infection symptoms that impact on acute and long-term outcomes of COVID-19 in this cohort after accounting for COVID-19 disease severity at enrolment. Understanding the disease mechanisms that explain these relationships is needed to inform targeted therapeutics to prevent and/or manage these complications.

**STRENGTHS AND LIMITATIONS OF THIS STUDY**

- The prospective, longitudinal nature of this study.
- Following a patient cohort from preadmission to 12-month recovery.
- Detailed documentation of the short-term and long-term outcomes of predominantly severe COVID-19 disease.
- The applicability of the findings to patients with milder disease is unclear.
- Loss to follow-up presents risk of ascertainment bias.

## Introduction

 Since the start of the COVID-19 pandemic, huge progress had been made in understanding the risk factors for developing and dying from severe disease. Early in the pandemic, dominant themes emerged reporting demographic factors such as gender, ethnicity, body mass, age and disease comorbidities associated with severe disease and reduced survival from acute infection.^1^ Admission severity scoring systems have been developed, some of which take certain demographic features into account to inform early triage and treatment pathways. Age (>50 years), male gender and presence of comorbidities are demographic features included in some scoring,^2^ however there is clearly regional variation in their applicability noting the global regional variation seen in risk factors,^3^ survival^4^ viral variant dominance^5^ and vaccination status^6^ as carefully monitored by global data reports.^7^ Applying a risk scoring system generated for one population may not be pertinent to another with a recent report of overestimation of death when a mortality scoring system developed in the UK from UK cohorts^8^ was applied in Australia.^9^ Local knowledge relevant to the population being treated is needed to ensure applicability.

As people recovered from acute infection, persistent and new sequelae manifest.^10 11^ These have been found to disproportionately affect certain patient groups and while severity of acute illness is a risk factor, several demographic factors also associated including female sex, middle age, two or more comorbidities and more acute severe illness.^12^ Global research efforts are ongoing to understand the sequelae of COVID and determine the biological mechanisms that explain them.^13^ The prospective long-term follow-up UK study of patients hospitalised with COVID^14^ reports that the persistent symptoms of breathlessness are not associated with objective measures of cardiorespiratory compromise^15^ and likely to be multifactorial with emotional and physical factors contributing.^16^ In contrast, cognitive impairment, while also multifactorial, had been associated with signatures of inflammation.^17^

Much of the learning about the risk factors associated with the clinical course of acute COVID is based on patients recruited early in the pandemic. There have been fewer studies evaluating the change in course with subsequent waves of infection with new viral variants and the impact of vaccination on the identified risk factors for severe acute disease and poor recovery. In addition, while there is now an extensive body of knowledge detailing the longer term sequelae of COVID-19, fewer studies detailing the entire clinical course from symptom onset to long-term recovery in well characterised clinical cohorts.

Understanding the demographic factors that predict COVID disease severity and acute clinical course is important for health service planning, informed triage at admission, understanding disease mechanisms and developing new therapies.

Here, we report the clinical results forming part of the prospective longitudinal multicentre study (the Humoral Immune Correlates of Protection study (HICC),^18^ describing the demographic, clinical course and outcome to 12 months after hospital discharge in a large cohort of patients with predominantly severe disease, recruited across three waves of the UK pandemic of whom 17% received at least one vaccination. We identify preadmissions risk factors that associate with COVID-19 acute complications, survival and long-term recovery with and without adjustment for WHO COVID-19 Severity Classification.^19^

## Methods summary

### Study design and approvals

This study is part of the HICC Study funded as an Urgent Public Health study.^18^ It is a prospective multicentre study in England, recruiting patients hospitalised with COVID-19 over the first 3 waves of the COVID-19 pandemic and following them through the acute phase of disease and in convalescence up to 1 year. As the study was observational, no sample size calculation was undertaken. While the original study design proposed recruitment of 500 patients with similar proportions of mild, moderate and severe disease, infection control measures implemented early in the pandemic limited opportunity to recruit patients with mild disease and moderate disease. As such recruitment was predominantly of hospitalised patients with severe disease. Patients were recruited from Royal Papworth, Burnley General Teaching, Watford General and Lister NHS Hospitals. Non-hospitalised patients were also recruited from Luton and Dunstable Community Healthcare Trust, representing patients with asymptomatic or mild disease. In this report, we describe the demographic of the patient cohort by disease severity, complications of acute disease survival to discharge and long-term recovery. We define the demographic risk factors for poor outcome of hospital admission and chronic sequelae of COVID-19. Inclusion criteria: COVID-19 disease. Exclusion criteria: refusal to provide informed consent. Enrolment was by written informed consent. Where the patient was unable to provide this due to the severity of their illness, consultee written informed consent was used to enrol the patient who was reconsented if/when able to provide their own consent, in accordance with the ethical approval of the study. Patient and public involvement was not possible in establishing the design or conduct of the study due to the nature of the pandemic.

### Participant details

425 COVID-19 patients were recruited to the study between April 2020 and December 2021 (four additional patients consented but CRFs were not completed). 405 COVID-19 patients were hospitalised and 24 recruited in the community. Demographic features including age, sex and ethnicity were recorded for all individuals at recruitment.

### Clinical data collection and processing

Following informed written consent, systematic detailed demographic and clinical profiling of the COVID-19 patients was undertaken including assessment of comorbidities, dates of onset of symptoms, first swab positive and hospital admission. Clinical course and outcome of hospitalisation. Comorbidity was recorded in accordance with the ISARIC-4C clinical characterisation protocol based on the Charlston comorbidity index.^8 20^ WHO criteria scoring of severity were conducted following the ‘COVID-19 Clinical Management: living guidance’.^3^ Individuals were assigned to wave 1 (admission 1 March 2020–31 August 2020), 2 (1 September 2020–28 April 2021) or 3 (29 April 2021–June 2022) of the COVID-19 pandemic based on the date of onset of symptoms^21^ which approximated to the evolution of dominant SARS-CoV-2 variants: using UK government surveillance data,^22^ wild type (WT) variant dominated in wave 1 (April 2020–December 2020), alpha (B1.1.7) variant in wave 2 (20 December 2020–28 April 2021) and delta (B1.617) variants in wave 3 (21 April 2021–14 June 2022) of the COVID-19 pandemic. 72 patients had received vaccination prior to admission (‘prevaccinated’) of whom two were in the Oxford Vaccine trial,^23^ the remainder will have been prioritised based on their increased risk of severe COVID in accordance with national guidelines. 49 had received two vaccination doses. The median time between first vaccination dose and symptom onset was 161 days (124–85, 95% CI) and between second vaccination dose and symptom onset was 108 days (82–123, 95% CI).

Following discharge, patients were invited to follow-up visits at 3–6 and 12 months to monitor for recovery from COVID-19. This was assessed by healthcare worker delivered questionnaires that the patient completed. Fields included persistent COVID-19-related symptoms subclassified as neurological, cardiac, gastrointestinal and COVID-19 symptoms, the MRC breathless score,^24^ a modified Patient Health Questionnaire (PHQ-9)^25^ /Generalised Anxiety Disorder Score^26^ to assess psychological well-being. In addition, patients were asked at each visit to grade their symptoms of breathlessness, cough, fatigue and sleep disturbance and indicate whether it was getting better, the same or worse. Vaccination status pre-COVID-19 and in follow-up was recorded for all individuals. Recruitment and Clinical Data were systematically recorded using the web-based platform Open Clinica^27^ including use of the self-reporting portal ‘participate’.^28^

### Data management and statistical analysis

Data generated through the CRFs were collated in Excel. GraphPad prism was used to generate the figures. All statistical analyses were performed using R software.^29^ For time course analysis, ‘date of symptom onset’ was used as day 0 (D0). Where date of symptom onset was missing or the individual was asymptomatic (n=34, 8% of the hospitalised cohort), a value of 14 days prior to recruitment was assigned as D0 to permit inclusion in time course data analysis. This was the median date value between symptom onset and recruitment for the overall cohort (14 days (12–14 days 95% CI), n=371). Unless stated otherwise, all tests were two-sided, and no multiplicity correction was used.

### Demographic profile by COVID-19 severity

In online supplemental tables S1.1 and S1.2, the demographic profile of the cohort is described and the relationship between severity (as a 5-level factor) and each demographic factor of interest (with numbers of levels ranging from 2 to 8) shown (analysed using contingency tables on which Fisher’s tests of independence were performed). The p value from the Fisher test was obtained through Monte Carlo simulation, considering 25 000 samples, as implemented in the *fisher.test* function of the R package *stats*, applied to the contingency table generated after discarding missing values. In online supplemental figure S1, the pattern of missingness was analysed using a heatmap that displays the missingness status through a colour code. A complete-case analysis, including all patients without missing observations in the variables of interest, was conducted for each analysis presented in this paper. As the level of missingness was generally low and clustered, its impact on the results is expected to be minimal.

### Clustering by symptoms and clustering by comorbidities

Symptoms and comorbidities presence/absence data were separately clustered by partitioning participants around medoids (as implemented in the *pamk* function of the R package *fpc*), based on Jaccard dissimilarity measures (obtained using the *dissimilarity* function from the R package *arules*), allowing for a suitable definition of the ‘distance’ between binary vectors. In online supplemental figure S2, the probability of observing each symptom and comorbidity per cluster was compared using proportion tests (as implemented in the *prop.test* function of the R package *stats*). The aim of the clustering was to help adjusting regression-like analyses for large differences in symptoms and comorbidities.

### Acute clinical course of hospitalised patients

In tables 1–6, for each clinical endpoint of interest, the presence or absence of the relevant event as a function of severity was analysed using adjusted and unadjusted logistic regression with a logit link (as fitted using the *glm* function of the R package *stats*, with the family argument set to *binomial*). Unadjusted analyses considered severity solely as an ordered categorical predictor (with *mild* as the reference group). Adjusted analyses controlled for sex, obesity, smoking status, duration of symptoms, ethnicity, vaccination status, COVID wave, age group and symptom and comorbidity clusters. OR estimates and 95% CIs, along with the corresponding Wald z-test, were calculated. Model checks, assessed using randomised quantile residuals from the R package *gamlss*, indicated a good fit of the model to the data across all clinical endpoints. In table 7, survival analyses, specifically Cox regressions, are used as an alternative to the logistic regressions considered in table 6. Cox regressions have the advantage of being able to handle time-to-event data and account for censoring, while logistic regressions are simpler, rely on different assumptions but do not consider time. Right-censored and left-truncated times to death were modelled using adjusted and unadjusted Cox regression analyses with a log link (as fitted using the *coxph* function of the R package *survival*). In this analysis, follow-up times for patients who were discharged or transferred were treated as right-censored, while time to death was treated as left-truncated, since patients who developed symptoms and died before reaching the hospital were not recorded. Both unadjusted and adjusted analyses considered the same predictors as those used in the logistic regression. HR estimates and 95% CIs, along with the corresponding Wald z-test, were calculated. In analyses of tables 1–7, predictors were simplified by combining levels or were removed when a strong imbalance in the number of successes and failures per combination of predictors resulted in unreasonably large standard errors due to numerical issues. This frequently occurred with the predictor severity, which was therefore treated as a 2- or 3-level factor in many analyses. As a complement to the *comprehensive*-adjusted analyses described above, we report two further sets of associations. First, we report crude associations between risk factor and outcome, that is, with no adjustment. Second, we fit a *conditional on severity* model where each potential risk factor is adjusted for severity. For each outcome, all three sets of associations use the same set of patients. With respect to statistical significance, results from both sets of analyses *- conditional on severity* and comprehensive - agree unless indicated otherwise in the text. As this is an observational study, we make no formal causal claims. In our adjusted *comprehensive* models, coefficients represent conditional associations, that is, the relationship between a risk factor and the outcome while holding all other included covariates constant. We acknowledge that if a covariate (such as WHO severity) acts as a mediator on the causal path, these estimates have a different interpretation (a ‘direct’ effect rather than a ‘total’ effect). Results should, therefore, be interpreted as hypothesis-generating.

**Table 1:** Thrombosis

Factor	Crude analysis		Conditional on severity		Adjusted ‘comprehensive’ analysis	
	OR (95% CI)	P value	OR (95% CI)	P value	OR (95% CI)	P value
(*Intercept*)	–	–	–	–	0.009 (0.001 to 0.153)	**0.0010**
Severity						
Mild/moderate	Ref (S2.1.1)		Ref (S2.1.1)		Ref	
Severe	1.278 (0.138 to 11.868)	0.8290	1.278 (0.138 to 11.868)	0.8290	1.551 (0.156 to 15.431)	0.7082
Critical ARDS*	1.329 (0.133 to 13.282)	0.8089	1.329 (0.133 to 13.282)	0.8089	1.366 (0.126 to 14.793)	0.7975
Critical Sepsis	22.683 (3.012 to 170.815)	**0.0024**	22.683 (3.012 to 170.815)	**0.0024**	21.426 (2.583 to 177.737)	**0.0045**
Sex						
Male	Ref (S2.1.2)		Ref (S2.1.3)		Ref	
Female	0.547 (0.300 to 0.997)	**0.0487**	0.670 (0.341 to 1.313)	0.2433	0.870 (0.410 to 1.847)	0.7169
Obesity						
No	Ref (S2.1.4)		Ref (S2.1.5)		Ref	
Yes	0.909 (0.509 to 1.623)	0.7472	0.637 (0.331 to 1.226)	0.1772	0.707 (0.332 to 1.510)	0.3708
Smoking status						
No	Ref (S2.1.6)		Ref (S2.1.7)		Ref	
Ex-smoker	0.813 (0.442 to 1.497)	0.5068	1.157 (0.570 to 2.348)	0.6858	1.305 (0.605 to 2.815)	0.4976
Current	0.315 (0.040 to 2.477)	0.2722	0.384 (0.042 to 3.491)	0.3951	0.419 (0.041 to 4.250)	0.4620
Duration symptoms						
Less than 1 week	Ref (S2.1.8)		Ref (S2.1.9)		Ref	
More than 1 week	1.069 (0.626 to 1.826)	0.8071	1.073 (0.584 to 1.972)	0.8211	1.212 (0.624 to 2.354)	0.5703
Ethnicity						
White	Ref (S2.1.10)		Ref (S2.1.11)		Ref	
Non-White	1.141 (0.543 to 2.394)	0.7278	1.009 (0.437 to 2.328)	0.9839	1.030 (0.410 to 2.590)	0.9496
Unknown	4.107 (2.128 to 7.924)	**<0.0001**	1.488 (0.719 to 3.079)	0.2845	1.111 (0.491 to 2.517)	0.8006
Vaccination						
No	Ref (S2.1.12)		Ref (S2.1.13)		Ref	
Yes	0.335 (0.116 to 0.968)	**0.0433**	0.814 (0.243 to 2.722)	0.7379	0.652 (0.150 to 2.836)	0.5689
Wave						
First	Ref (S2.1.14)		Ref (S2.1.15)		Ref	
Second	0.532 (0.291 to 0.971)	**0.0399**	0.558 (0.281 to 1.107)	0.0949	0.720 (0.341 to 1.519)	0.3887
Third	0.297 (0.133 to 0.667)	**0.0033**	0.806 (0.303 to 2.147)	0.6664	1.194 (0.358 to 3.982)	0.7726
Age						
<40 years	Ref (S2.1.16)		Ref (S2.1.17)		Ref	
40–60 years	1.767 (0.740 to 4.220)	0.1996	1.936 (0.736 to 5.089)	0.1805	2.254 (0.771 to 6.589)	0.1375
>60 years	0.871 (0.330 to 2.299)	0.7796	0.991 (0.340 to 2.890)	0.9866	1.281 (0.363 to 4.518)	0.7004
Clustering symptoms						
Cluster 1	Ref (S2.1.18)		Ref (S2.1.19)		Ref	
Cluster 2	5.968 (2.932 to 12.149)	**<0.0001**	2.983 (1.366 to 6.514)	**0.0061**	2.654 (1.159 to 6.076)	**0.0209**
Clustering comorbidities						
Cluster 1	Ref (S2.1.20)		Ref (S2.1.21)		Ref	
Cluster 2	1.399 (0.818 to 2.394)	0.2205	1.582 (0.852 to 2.938)	0.1464	1.347 (0.665 to 2.729)	0.4077

Defining the relationship between preadmission risk factors and the specified acute clinical outcome in Hospitalised Patients with and without controlling for COVID-19 Severity Score and other potential confounders.

Statistical analysis: OR estimates and 95% CIs, along with the raw p-value from the corresponding Wald z-test, are presented for crude associations (left-hand column), logistic regression conditional on severity (centre column) and fully adjusted logistic regression (right-hand column). These analyses aim to predict the presence or absence of the clinical endpoint of interest, controlling for severity alone and controlling for severity plus additional potential confounders.

Crude=single-predictor model (factor alone); Conditional on severity=factor adjusted for WHO severity only; Adjusted ‘comprehensive’ = all listed predictors mutually adjusted. Reference-level cells link (S2.k.m) to the supplementary table holding that model’s full coefficients incl. intercept. Significant p-values (<0.05) in bold and highlighted.

* Acute Respiratory Distress Syndrome (ARDS).

**Table 2:** Neurological events

Factor	Crude analysis		Conditional on severity		Adjusted ‘comprehensive’ analysis	
	OR (95% CI)	P value	OR (95% CI)	P value	OR (95% CI)	P value
(*Intercept*)	–	–	–	–	0.022(0.003 to 0.183)	**0.0004**
Severity						
Other	Ref (S2.2.1)		Ref (S2.2.1)		Ref	
Critical sepsis	13.527 (4.647 to 39.375)	**<0.0001**	13.527 (4.647 to 39.375)	**<0.0001**	15.977 (4.426 to 57.668)	**<0.0001**
Sex						
Male	Ref (S2.2.2)		Ref (S2.2.3)		Ref	
Female	0.741 (0.342 to 1.605)	0.4475	0.957 (0.422 to 2.169)	0.9159	0.731 (0.286 to 1.868)	0.5129
Obesity						
No	Ref (S2.2.4)		Ref (S2.2.5)		Ref	
Yes	2.145 (1.048 to 4.389)	**0.0367**	1.824 (0.855 to 3.889)	0.1198	1.719 (0.708 to 4.176)	0.2315
Smoking status						
No	Ref (S2.2.6)		Ref (S2.2.7)		Ref	
Smoke (days)	0.770 (0.347 to 1.711)	0.5218	1.032 (0.442 to 2.410)	0.9416	1.358 (0.532 to 3.463)	0.5219
Duration symptoms						
Less than 1 week	Ref (S2.2.8)		Ref (S2.2.9)		Ref	
More than 1 week	0.764 (0.376 to 1.552)	0.4565	0.732 (0.346 to 1.547)	0.4137	0.804 (0.357 to 1.809)	0.5982
Ethnicity						
White	Ref (S2.2.10)		Ref (S2.2.11)		Ref	
Non-White	1.738 (0.677 to 4.462)	0.2506	1.623 (0.599 to 4.397)	0.3409	1.714 (0.579 to 5.070)	0.3305
Unknown	4.320 (1.882 to 9.914)	**0.0006**	1.820 (0.757 to 4.375)	0.1808	1.812 (0.665 to 4.936)	0.2451
Vaccination						
No	Ref (S2.2.12)		Ref (S2.2.13)		Ref	
Yes	0.172 (0.023 to 1.289)	0.0868	0.373 (0.047 to 2.971)	0.3514	0.213 (0.023 to 1.995)	0.1757
Wave						
First	Ref (S2.2.14)		Ref (S2.2.15)		Ref	
Second	0.650 (0.288 to 1.472)	0.3019	0.737 (0.313 to 1.736)	0.4846	0.762 (0.293 to 1.979)	0.5766
Third	0.566 (0.208 to 1.537)	0.2638	1.635 (0.526 to 5.081)	0.3951	3.001 (0.774 to 11.630)	0.1118
Age						
<40 years	Ref (S2.2.16)		Ref (S2.2.17)		Ref	
40–60 years	1.004 (0.386 to 2.611)	0.9934	0.953 (0.342 to 2.655)	0.9262	1.151 (0.362 to 3.661)	0.8116
>60 years	0.185 (0.044 to 0.776)	**0.0210**	0.184 (0.042 to 0.814)	**0.0257**	0.222 (0.041 to 1.196)	0.0799
Clustering symptoms						
Cluster 1	Ref (S2.2.18)		Ref (S2.2.19)		Ref	
Cluster 2	2.753 (1.209 to 6.269)	**0.0159**	1.202 (0.490 to 2.953)	0.6876	0.815 (0.294 to 2.262)	0.6948
Clustering comorbidities						
Cluster 1	Ref (S2.2.20)		Ref (S2.2.21)		Ref	
Cluster 2	0.773 (0.364 to 1.643)	0.5035	0.762 (0.345 to 1.683)	0.5014	0.495 (0.198 to 1.240)	0.1332

Defining the relationship between preadmission risk factors and the specified acute clinical outcome in Hospitalised Patients with and without controlling for COVID-19 Severity Score and other potential confounders.

Statistical analysis: OR estimates and 95% CIs, along with the raw p value from the corresponding Wald z-test, are presented for crude associations (left-hand column), logistic regression conditional on severity (centre column) and fully adjusted logistic regression (right-hand column). These analyses aim to predict the presence or absence of the clinical endpoint of interest, controlling for severity alone and controlling for severity plus additional potential confounders.

Crude=single-predictor model (factor alone); conditional on severity=factor adjusted for WHO severity only; adjusted ‘comprehensive’ = all listed predictors mutually adjusted. Reference-level cells link (S2.k.m) to the supplementary table holding that model’s full coefficients including intercept. Significant p values (<0.05) in bold and highlighted.

**Table 3:** Renal complications

Factor	Crude analysis		Conditional on severity		Adjusted ‘comprehensive’ analysis	
	OR (95% CI)	P value	OR (95% CI)	P value	OR (95% CI)	P value
(*Intercept*)	–	–	–	–	0.015(0.002 to 0.117)	**0.0001**
Severity						
Other	Ref (S2.3.1)		Ref (S2.3.1)		Ref	
Critical sepsis	18.639 (8.536 to 40.701)	**<0.0001**	18.639 (8.536 to 40.701)	**<0.0001**	19.659 (7.565 to 51.083)	**<0.0001**
Sex						
Male	Ref (S2.3.2)		Ref (S2.3.3)		Ref	
Female	0.534 (0.293 to 0.971)	**0.0397**	0.648 (0.330 to 1.271)	0.2071	0.619 (0.287 to 1.337)	0.2222
Obesity						
No	Ref (S2.3.4)		Ref (S2.3.5)		Ref	
Yes	1.699 (0.984 to 2.933)	0.0572	1.431 (0.766 to 2.673)	0.2612	2.465 (1.118 to 5.436)	**0.0253**
Smoking status						
No	Ref (S2.3.6)		Ref (S2.3.7)		Ref	
Ex-smoker	0.599 (0.315 to 1.141)	0.1189	0.777 (0.375 to 1.612)	0.4987	0.699 (0.305 to 1.603)	0.3976
Current	0.638 (0.137 to 2.968)	0.5667	0.892 (0.154 to 5.170)	0.8983	0.928 (0.140 to 6.170)	0.9385
Duration symptoms						
Less than 1 week	Ref (S2.3.8)		Ref (S2.3.9)		Ref	
More than 1 week	1.103 (0.647 to 1.881)	0.7174	1.118 (0.609 to 2.054)	0.7180	1.407 (0.691 to 2.864)	0.3467
Ethnicity						
White	Ref (S2.3.10)		Ref (S2.3.11)		Ref	
Non-White	0.633 (0.268 to 1.495)	0.2967	0.492 (0.193 to 1.254)	0.1372	0.755 (0.264 to 2.164)	0.6015
Unknown	4.855 (2.540 to 9.279)	**<0.0001**	1.785 (0.865 to 3.685)	0.1170	2.020 (0.867 to 4.706)	0.1031
Vaccination						
No	Ref (S2.3.12)		Ref (S2.3.13)		Ref	
Yes	0.537 (0.219 to 1.321)	0.1760	1.587 (0.529 to 4.761)	0.4103	1.761 (0.439 to 7.073)	0.4247
Wave						
First	Ref (S2.3.14)		Ref (S2.3.15)		Ref	
Second	0.332 (0.182 to 0.607)	**0.0003**	0.298 (0.148 to 0.600)	**0.0007**	0.298 (0.134 to 0.662)	**0.0030**
Third	0.252 (0.116 to 0.547)	**0.0005**	0.663 (0.257 to 1.710)	0.3949	0.984 (0.277 to 3.495)	0.9804
Age						
<40 years	Ref (S2.3.16)		Ref (S2.3.17)		Ref	
40–60 years	1.141 (0.491 to 2.651)	0.7586	1.107 (0.430 to 2.851)	0.8326	1.082 (0.347 to 3.370)	0.8923
>60 years	1.310 (0.540 to 3.179)	0.5500	1.713 (0.623 to 4.712)	0.2972	2.640 (0.707 to 9.862)	0.1487
Clustering symptoms						
Cluster 1	Ref (S2.3.18)		Ref (S2.3.19)		Ref	
Cluster 2	6.115 (3.006 to 12.441)	**<0.0001**	3.007 (1.384 to 6.537)	**0.0054**	3.320 (1.371 to 8.041)	**0.0078**
Clustering comorbidities						
Cluster 1	Ref (S2.3.20)		Ref (S2.3.21)		Ref	
Cluster 2	0.672 (0.382 to 1.184)	0.1691	0.610 (0.322 to 1.156)	0.1299	0.585 (0.264 to 1.297)	0.1873

Defining the relationship between preadmission risk factors and the specified acute clinical outcome in Hospitalised Patients with and without controlling for COVID-19 Severity Score and other potential confounders.

Statistical analysis: OR estimates and 95% CIs, along with the raw p value from the corresponding Wald z-test, are presented for crude associations (left-hand column), logistic regression conditional on severity (centre column) and fully adjusted logistic regression (right-hand column). These analyses aim to predict the presence or absence of the clinical endpoint of interest, controlling for severity alone and controlling for severity plus additional potential confounders.

Crude=single-predictor model (factor alone); conditional on severity=factor adjusted for WHO severity only; adjusted ‘comprehensive’ = all listed predictors mutually adjusted. Reference-level cells link (S2.k.m) to the supplementary table holding that model’s full coefficients including intercept. Significant p values (<0.05) in bold and highlighted.

**Table 4:** Cardiac events

Factor	Crude analysis		Conditional on severity		Adjusted ‘comprehensive’ analysis	
	OR (95% CI)	P value	OR (95% CI)	P value	OR (95% CI)	P value
(*Intercept*)	–	–	–	–	0.014 (0.001 to 0.247)	**0.0036**
Severity						
Mild/moderate	Ref (S2.4.1)		Ref (S2.4.1)		Ref	
Severe	0.949 (0.095 to 9.455)	0.9644	0.949 (0.095 to 9.455)	0.9644	1.130 (0.105 to 12.207)	0.9196
Critical ARDS*	1.329 (0.133 to 13.282)	0.8089	1.329 (0.133 to 13.282)	0.8089	1.405 (0.132 to 14.914)	0.7780
Critical sepsis	6.948 (0.907 to 53.235)	0.0621	6.948 (0.907 to 53.235)	0.0621	6.477 (0.781 to 53.715)	0.0834
Sex						
Male	Ref (S2.4.2)		Ref (S2.4.3)		Ref	
Female	0.777 (0.357 to 1.691)	0.5251	0.955 (0.426 to 2.141)	0.9119	0.992 (0.417 to 2.358)	0.9856
Obesity						
No	Ref (S2.4.4)		Ref (S2.4.5)		Ref	
Yes	1.505 (0.719 to 3.150)	0.2778	1.264 (0.587 to 2.721)	0.5497	1.468 (0.622 to 3.465)	0.3806
Smoking status						
No	Ref (S2.4.6)		Ref (S2.4.7)		Ref	
Ex-smoker	0.546 (0.217 to 1.372)	0.1982	0.660 (0.255 to 1.706)	0.3911	0.652(0.241 to 1.769)	0.4013
Current	0.683 (0.085 to 5.466)	0.7191	0.881 (0.102 to 7.593)	0.9079	1.062(0.114 to 9.915)	0.9577
Duration symptoms						
Less than 1 week	Ref (S2.4.8)		Ref (S2.4.9)		Ref	
More than 1 week	0.625(0.304 to 1.285)	0.2011	0.603(0.286 to 1.271)	0.1836	0.657(0.298 to 1.449)	0.2983
Ethnicity						
White	Ref (S2.4.10)		Ref (S2.4.11)		Ref	
Non-White	1.203(0.457 to 3.168)	0.7077	1.106(0.406 to 3.015)	0.8437	1.065(0.345 to 3.283)	0.9133
Unknown	2.477(1.043 to 5.880)	**0.0398**	1.168(0.468 to 2.914)	0.7395	1.036(0.376 to 2.857)	0.9449
Vaccination						
No	Ref (S2.4.12)		Ref (S2.4.13)		Ref	
Yes	0.850(0.285 to 2.534)	0.7699	1.806(0.541 to 6.029)	0.3366	2.944(0.630 to 13.769)	0.1701
Wave						
First	Ref (S2.4.14)		Ref (S2.4.15)		Ref	
Second	0.695(0.310 to 1.560)	0.3782	0.780(0.339 to 1.797)	0.5599	0.854(0.342 to 2.132)	0.7347
Third	0.394(0.131 to 1.188)	0.0983	0.824(0.249 to 2.734)	0.7523	0.562(0.122 to 2.598)	0.4611
Age						
<40 years	Ref (S2.4.16)		Ref (S2.4.17)		Ref	
40–60 years	1.228(0.399 to 3.784)	0.7205	1.190(0.373 to 3.795)	0.7693	1.335(0.372 to 4.793)	0.6575
>60 years	0.926(0.270 to 3.174)	0.9032	1.025(0.288 to 3.652)	0.9694	1.585(0.362 to 6.938)	0.5407
Clustering symptoms						
Cluster 1	Ref (S2.4.18)		Ref (S2.4.19)		Ref	
Cluster 2	3.888(1.562 to 9.676)	**0.0035**	2.225(0.840 to 5.896)	0.1076	2.006(0.719 to 5.601)	0.1837
Clustering comorbidities						
Cluster 1	Ref (S2.4.20)		Ref (S2.4.21)		Ref	
Cluster 2	1.637(0.797 to 3.363)	0.1799	1.721(0.816 to 3.632)	0.1542	1.969(0.851 to 4.558)	0.1136

Defining the relationship between preadmission risk factors and the specified acute clinical outcome in Hospitalised Patients with and without controlling for COVID-19 Severity Score and other potential confounders.

Statistical Analysis: OR estimates and 95% CIs, along with the raw p-value from the corresponding Wald z-test, are presented for crude associations (left-hand column), logistic regression conditional on severity (centre column) and fully adjusted logistic regression (right-hand column). These analyses aim to predict the presence or absence of the clinical endpoint of interest, controlling for severity alone and controlling for severity plus additional potential confounders.

Crude=single-predictor model (factor alone); Conditional on severity=factor adjusted for WHO severity only; Adjusted ‘comprehensive’ = all listed predictors mutually adjusted. Reference-level cells link (S2.k.m) to the supplementary table holding that model’s full coefficients incl. intercept. Significant p-values (<0.05) in bold and highlighted.

* Acute Respiratory Distress Syndrome (ARDS).

**Table 5:** Pneumothorax

Factor	Crude analysis		Conditional on severity		Adjusted ‘comprehensive’ analysis	
	OR (95% CI)	P value	OR (95% CI)	P value	OR (95% CI)	P value
(*Intercept*)	–	–	–	–	0.018 (0.002 to 0.180)	**0.0006**
Severity						
Other	Ref (S2.5.1)		Ref (S2.5.1)		Ref	
Critical sepsis	5.835 (2.113 to 16.115)	**0.0007**	5.835 (2.113 to 16.115)	**0.0007**	4.248 (1.224 to 14.751)	**0.0227**
Sex						
Male	Ref (S2.5.2)		Ref (S2.5.3)		Ref	
Female	0.974 (0.401 to 2.366)	0.9535	1.198 (0.480 to 2.987)	0.6987	0.946 (0.331 to 2.703)	0.9167
Obesity						
No	Ref (S2.5.4)		Ref (S2.5.5)		Ref	
Yes	1.200 (0.493 to 2.922)	0.6880	1.011 (0.406 to 2.514)	0.9817	0.773 (0.267 to 2.241)	0.6353
Smoking status						
No	Ref (S2.5.6)		Ref (S2.5.7)		Ref	
Smoke (days)	0.590 (0.213 to 1.633)	0.3100	0.723 (0.255 to 2.047)	0.5412	0.824 (0.268 to 2.535)	0.7360
Duration symptoms						
Less than 1 week	Ref (S2.5.8)		Ref (S2.5.9)		Ref	
More than 1 week	0.319 (0.128 to 0.796)	**0.0143**	0.301 (0.118 to 0.765)	**0.0117**	0.322(0.120 to 0.869)	**0.0252**
Ethnicity						
White	Ref (S2.5.10)		Ref (S 2.5.11)		Ref	
Non-White	0.883(0.242 to 3.228)	0.8508	0.809(0.217 to 3.014)	0.7517	0.534(0.130 to 2.192)	0.3836
Unknown	3.318(1.282 to 8.592)	**0.0135**	1.707(0.620 to 4.699)	0.3009	1.008(0.313 to 3.254)	0.9888
Vaccination						
No	Ref (S2.5.12)		Ref (S2.5.13)		Ref	
Yes	0.269(0.035 to 2.042)	0.2042	0.476(0.060 to 3.787)	0.4829	0.307(0.031 to 3.014)	0.3110
Wave						
First	Ref (S2.5.14)		Ref (S2.5.15)		Ref	
Second	0.850(0.303 to 2.384)	0.7580	0.962(0.336 to 2.754)	0.9420	1.294(0.400 to 4.186)	0.6670
Third	0.939(0.290 to 3.036)	0.9163	2.109(0.588 to 7.559)	0.2519	3.638(0.790 to 16.762)	0.0976
Age						
<40 years	Ref (S2.5.16)		Ref (S2.5.17)		Ref	
40–60 years	1.092(0.351 to 3.398)	0.8786	1.059(0.330 to 3.393)	0.9237	1.512(0.384 to 5.955)	0.5540
>60 years	0.095(0.010 to 0.879)	**0.0381**	0.099(0.011 to 0.929)	**0.0429**	0.162(0.014 to 1.810)	0.1394
Clustering symptoms						
Cluster 1	Ref (S2.5.18)		Ref (S2.5.19)		Ref	
Cluster 2	5.644(1.645 to 19.365)	**0.0059**	3.428(0.948 to 12.398)	0.0603	3.258(0.794 to 13.370)	0.1012
Clustering comorbidities						
Cluster 1	Ref (S2.5.20)		Ref (S2.5.21)		Ref	
Cluster 2	1.566(0.670 to 3.659)	0.3002	1.613(0.677 to 3.845)	0.2802	1.155(0.425 to 3.141)	0.7775

Defining the relationship between preadmission risk factors and the specified acute clinical outcome in Hospitalised Patients with and without controlling for COVID-19 Severity Score and other potential confounders.

Statistical analysis: OR estimates and 95% CIs, along with the raw p value from the corresponding Wald z-test, are presented for crude associations (left-hand column), logistic regression conditional on severity (centre column) and fully adjusted logistic regression (right-hand column). These analyses aim to predict the presence or absence of the clinical endpoint of interest, controlling for severity alone and controlling for severity plus additional potential confounders.

Crude=single-predictor model (factor alone); conditional on severity=factor adjusted for WHO severity only; adjusted ‘comprehensive’=all listed predictors mutually adjusted. Reference-level cells link (S2.k.m) to the supplementary table holding that model’s full coefficients including intercept. Significant p values (<0.05) in bold and highlighted.

**Table 6:** Survival to discharge

Factor	Crude analysis		Conditional on severity		Adjusted ‘comprehensive’ analysis	
	OR (95% CI)	P value	OR (95% CI)	P value	OR (95% CI)	P value
(*Intercept*)	–	–	–	–	184.181(20.646 to 1643.024)	**<0.0001**
Severity						
Non-critical pneumonia	Ref (S2.6.1)		Ref (S2.6.1)		Ref	
Critical ARDS*	0.446 (0.131 to 1.513)	0.1951	0.446 (0.131 to 1.513)	0.1951	0.334 (0.088 to 1.266)	0.1068
Critical sepsis	0.051 (0.020 to 0.131)	**<0.0001**	0.051 (0.020 to 0.131)	**<0.0001**	0.040 (0.012 to 0.134)	**<0.0001**
Sex						
Male	Ref (S2.6.2)		Ref (S2.6.3)		Ref	
Female	1.315 (0.754 to 2.294)	0.3342	0.999 (0.531 to 1.879)	0.9970	1.220 (0.568 to 2.617)	0.6104
Obesity						
No	Ref (S2.6.4)		Ref (S2.6.5)		Ref	
Yes	0.910 (0.525 to 1.578)	0.7363	1.254 (0.675 to 2.328)	0.4737	0.958 (0.454 to 2.018)	0.9095
Smoking status						
No	Ref (S2.6.6)		Ref (S2.6.7)		Ref	
Smoke (days)	1.647 (0.907 to 2.992)	0.1014	1.288 (0.659 to 2.517)	0.4589	1.264 (0.587 to 2.722)	0.5493
Duration symptoms						
Less than 1 week	Ref (S2.6.8)		Ref (S2.6.9)		Ref	
More than 1 week	0.936 (0.556 to 1.576)	0.8038	0.891 (0.495 to 1.603)	0.7000	0.786 (0.393 to 1.570)	0.4948
Ethnicity						
White	Ref (S2.6.10)		Ref (S2.6.11)		Ref	
Non-white	1.384 (0.582 to 3.289)	0.4621	1.620 (0.645 to 4.070)	0.3050	1.283 (0.467 to 3.525)	0.6290
Unknown	0.095 (0.048 to 0.185)	**<0.0001**	0.210 (0.100 to 0.438)	**<0.0001**	0.152 (0.066 to 0.353)	**<0.0001**
Vaccination						
No	Ref (S2.6.12)		Ref (S2.6.13)		Ref	
Yes	1.718 (0.739 to 3.996)	0.2090	0.577 (0.206 to 1.614)	0.2949	0.853 (0.225 to 3.244)	0.8160
Wave						
First	Ref (S2.6.14)		Ref (S2.6.15)		Ref	
Second	1.329 (0.726 to 2.433)	0.3572	1.140 (0.581 to 2.237)	0.7028	0.887 (0.396 to 1.985)	0.7699
Third	2.164 (1.009 to 4.641)	**0.0474**	0.639 (0.250 to 1.634)	0.3500	0.265 (0.068 to 1.027)	0.0546
Age						
<40 years	Ref (S2.6.16)		Ref (S2.6.17)		Ref	
40–60 years	0.449 (0.167 to 1.209)	0.1132	0.412 (0.142 to 1.192)	0.1017	0.361 (0.102 to 1.280)	0.1146
>60 years	0.393 (0.141 to 1.094)	0.0738	0.262 (0.086 to 0.802)	**0.0190**	0.205 (0.050 to 0.834)	**0.0269**
Clustering symptoms						
Cluster 1	Ref (S2.6.18)		Ref (S2.6.19)		Ref	
Cluster 2	0.339 (0.190 to 0.607)	**0.0003**	0.772 (0.394 to 1.513)	0.4513	0.774 (0.355 to 1.690)	0.5205
Clustering comorbidities						
Cluster 1	Ref (S2.6.20)		Ref (S2.6.21)		Ref	
Cluster 2	2.080 (1.161 to 3.728)	**0.0139**	2.463 (1.286 to 4.719)	**0.0066**	2.679 (1.206 to 5.948)	**0.0155**

Defining the relationship between preadmission risk factors and the specified acute clinical outcome in Hospitalised Patients with and without controlling for COVID-19 Severity Score and other potential confounders.

Statistical analysis: OR estimates and 95% CIs, along with the raw p value from the corresponding Wald z-test, are presented for crude associations (left-hand column), logistic regression conditional on severity (centre column) and fully adjusted logistic regression (right-hand column). These analyses aim to predict the presence or absence of the clinical endpoint of interest, controlling for severity alone and controlling for severity plus additional potential confounders.

Crude=single-predictor model (factor alone); conditional on severity=factor adjusted for WHO severity only; adjusted ‘comprehensive’=all listed predictors mutually adjusted. Reference-level cells link (S2.k.m) to the supplementary table holding that model’s full coefficients including intercept. Significant p values (<0.05) in bold and highlighted.

* Acute Respiratory Distress Syndrome (ARDS).

**Table 7:** Time to death (Cox)

Factor	Crude analysis		Conditional on severity		Adjusted ‘comprehensive’ analysis	
	HR (95% CI)	P value	HR (95% CI)	P value	HR (95% CI)	P value
(*Intercept*)	–	–	–	–		
Severity						
Non-critical pneumonia	Ref (S2.7.1)		Ref (S2.7.1)		Ref	
Critical ARDS*	1.524 (0.378 to 6.144)	0.5536	1.524 (0.378 to 6.144)	0.5536	3.263 (0.472 to 22.546)	0.2305
Critical sepsis	2.694 (0.808 to 8.985)	0.1068	2.694 (0.808 to 8.985)	0.1068	30.920 (3.128 to 305.606)	**0.0033**
Sex						
Male	Ref (S2.7.2)		Ref (S2.7.3)		Ref	
Female	0.690 (0.243 to 1.962)	0.4866	0.691 (0.243 to 1.963)	0.4873	0.384 (0.116 to 1.272)	0.1175
Obesity						
No	Ref (S2.7.4)		Ref (S2.7.5)		Ref	
Yes	1.162 (0.425 to 3.175)	0.7701	1.075 (0.393 to 2.943)	0.8873	2.013 (0.634 to 6.394)	0.2353
Smoking status						
No	Ref (S2.7.6)		Ref (S2.7.7)		Ref	
Smoke (days)	1.417 (0.536 to 3.746)	0.4821	1.891 (0.679 to 5.266)	0.2229	2.151 (0.581 to 7.962)	0.2515
Duration symptoms						
Less than 1 week	Ref (S2.7.8)		Ref (S2.7.9)		Ref	
More than 1 week	0.838 (0.310 to 2.263)	0.7274	0.755 (0.267 to 2.139)	0.5972	0.180 (0.041 to 0.789)	**0.0230**
Ethnicity						
White	Ref (S2.7.10)		Ref (S2.7.11)		Ref	
Non-White	0.475 (0.136 to 1.662)	0.2439	0.403 (0.114 to 1.428)	0.1591	0.378 (0.089 to 1.603)	0.1869
Vaccination						
No	Ref (S2.7.12)		Ref (S2.7.13)		Ref	
Yes	1.281 (0.450 to 3.644)	0.6425	1.488 (0.514 to 4.304)	0.4633	6.360 (1.226 to 33.007)	**0.0277**
Clustering symptoms						
Cluster 1	Ref (S2.7.14)		Ref (S2.7.15)		Ref	
Cluster 2	0.330 (0.074 to 1.466)	0.1451	0.313 (0.070 to 1.400)	0.1284	0.243 (0.044 to 1.339)	0.1043
Clustering comorbidities						
Cluster 1	Ref (S2.7.16)		Ref (S2.7.17)		Ref	
Cluster 2	0.158 (0.021 to 1.195)	0.0739	0.128 (0.017 to 0.972)	**0.0469**	0.030 (0.003 to 0.318)	**0.0035**

Defining the relationship between preadmission risk factors and the specified acute clinical outcome in Hospitalised Patients with and without controlling for COVID-19 Severity Score and other potential confounders.

Survival model: HR estimates and 95% CIs, along with the raw p value from the corresponding Wald z-test are presented for crude associations (left-hand column), Cox regression conditional on severity (centre column) and fully Cox regression analyses (right-hand column). These analyses aim to assess the hazard of death, controlling for severity alone and controlling for severity in addition to other potential confounders. ‘Ref’ refers to the comparator reference group.

Crude=single-predictor model (factor alone); conditional on severity=factor adjusted for WHO severity only; adjusted ‘comprehensive’ = all listed predictors mutually adjusted. Reference-level cells link (S2.k.m) to the supplementary table holding that model’s full coefficients including intercept. Significant p-values (<0.05) in bold and highlighted.

* Acute Respiratory Distress Syndrome (ARDS).

### Long-term recovery from COVID-19

In tables 8 and 9, adjusted and unadjusted generalised linear mixed models for binary endpoints, as fitted using the *glmer* function from the *lme4* package with the family argument set to *binomial*, were used to analyse the effect of severity on two recovery endpoints—the MRC Breathlessness score ranged from 0 to 4 and was dichotomised into 0 (no breathlessness) to 1–4 (mild to severe breathlessness), and mood disorder from 0 (least disorder) to 16 based on four parameters (interest, feeling down, nervous and worrying)—measured at up to three time points per patient and was dichotomised into 0 vs >0. All models treated patients as random effects. Unadjusted analyses solely predicted the probability of success based on severity (a three-level factor with mild as the reference group) and time (a factor with levels 3, 6 and 12 months, with 3 months as the reference group), while adjusted analyses further controlled for sex, obesity, smoking status, duration of symptoms, ethnicity, vaccination status, age group, and symptom and comorbidity clusters. OR estimates and 95% CIs, along with the corresponding Wald z-test, were calculated, and p values were not adjusted for multiplicity. Similarly to the logistic regression and time to event analyses above, we additionally fit a model for each risk factor ‘conditional on severity’, ie, adjusting for severity and that risk factor only, indicating differences in the text.

**Table 8:** MRC Breathlessness Score

Factor	Crude analysis		Conditional on severity		Adjusted ‘comprehensive’ analysis	
	OR (95% CI)	P value	OR (95% CI)	P value	OR (95% CI)	P value
(*Intercept*)	–	–	–	–	0.002 (0.000 to 2.191)	0.0828
Severity						
Non-critical pneumonia	Ref (S2.8.1)		Ref (S2.8.1)		Ref	
Critical ARDS	1.626 (0.186 to 14.176)	0.6602	1.626 (0.186 to 14.176)	0.6602	2.819 (0.188 to 42.207)	0.4528
Critical sepsis	31.786 (2.704 to 373.638)	**0.0059**	31.786 (2.704 to 373.638)	**0.0059**	87.818 (1.511 to 5102.447)	**0.0308**
Sex						
Male	Ref (S2.8.2)		Ref (S2.8.3)		Ref	
Female	27.530 (2.727 to 277.951)	**0.0049**	51.944 (3.076 to 877.188)	**0.0062**	99.796 (2.416 to 4122.305)	**0.0153**
Obesity						
No	Ref (S2.8.4)		Ref (S2.8.5)		Ref	
Yes	9.695 (1.100 to 85.413)	**0.0407**	5.648 (0.835 to 38.212)	0.0759	3.754 (0.324 to 43.567)	0.2902
Smoking status						
No	Ref (S2.8.6)		Ref (S2.8.7)		Ref	
Smoke (days)	0.535 (0.105 to 2.716)	0.4506	0.563 (0.095 to 3.335)	0.5271	0.977 (0.112 to 8.564)	0.9836
Duration symptoms						
Less than 1 week	Ref (S2.8.8)		Ref (S2.8.9)		Ref	
More than 1 week	2.266 (0.492 to 10.444)	0.2939	2.001 (0.375 to 10.670)	0.4165	5.851 (0.467 to 73.392)	0.1710
Ethnicity						
White	Ref (S2.8.10)		Ref (S2.8.11)		Ref	
Non-white	2.400 (0.403 to 14.288)	0.3361	1.324 (0.201 to 8.712)	0.7701	3.170 (0.207 to 48.506)	0.4072
Vaccination						
No	Ref (S2.8.12)		Ref (S2.8.13)		Ref	
Yes	0.660 (0.075 to 5.812)	0.7081	1.288 (0.110 to 15.133)	0.8406	1.056 (0.027 to 42.060)	0.9769
Wave						
First	Ref (S2.8.14)		Ref (S2.8.15)		Ref	
Second	2.582 (0.351 to 19.002)	0.3516	3.693 (0.477 to 28.600)	0.2110	7.897 (0.451 to 138.410)	0.1573
Third	0.174 (0.013 to 2.420)	0.1931	0.471 (0.028 to 7.961)	0.6016	2.120 (0.037 to 122.825)	0.7167
Age						
<40 years	Ref (S2.8.16)		Ref (S2.8.17)		Ref	
40-60 yrs	5.395 (0.475 to 61.305)	0.1741	7.776 (0.468 to 129.199)	0.1526	4.915 (0.156 to 155.010)	0.3659
>60 years	6.412 (0.492 to 83.540)	0.1560	17.650 (0.758 to 410.773)	0.0738	12.594 (0.180 to 880.664)	0.2424
Clustering symptoms						
Cluster 1	Ref (S2.8.18)		Ref (S2.8.19)		Ref	
Cluster 2	2.237 (0.476 to 10.517)	0.3080	0.959 (0.171 to 5.373)	0.9616	4.613 (0.355 to 59.965)	0.2427
Clustering comorbidities						
Cluster 1	Ref (S2.8.20)		Ref (S2.8.21)		Ref	
Cluster 2	0.057 (0.003 to 0.994)	**0.0496**	0.058 (0.006 to 0.540)	**0.0124**	0.128 (0.011 to 1.478)	0.0996

Defining the relationship between pre-admission risk factors and recovery from COVID-19 over time up to 12 months after admission with and without controlling for COVID-19 Severity Score and other potential confounders.

OR estimates and 95% CIs, along with the raw p value from the corresponding Wald z-test, are presented for crude associations (left-hand column), conditional on severity (centre column) and fully adjusted logistic regression (right-hand column). These analyses aim to predict the presence or absence of the clinical endpoint of interest controlling for severity alone and controlling for severity plus additional potential confounders.

Crude=single-predictor model (factor alone); conditional on severity=factor adjusted for WHO severity only; adjusted ‘comprehensive’=all listed predictors mutually adjusted. Reference-level cells link (S2.k.m) to the supplementary table holding that model’s full coefficients including intercept. Significant p values (<0.05) in bold and highlighted.

**Table 9:** Mood disorder Score

Factor	Crude analysis		Conditional on severity		Adjusted ‘comprehensive’ analysis	
	OR (95% CI)	P value	OR (95% CI)	P value	OR (95% CI)	P value
(*Intercept*)	–	–	–	–	2.931(0.111 to 77.714)	0.5202
Severity						
Non-critical pneumonia	Ref (S2.9.1)		Ref (S2.9.1)		Ref	
Critical ARDS*	0.726 (0.174 to 3.034)	0.6611	0.726 (0.174 to 3.034)	0.6611	0.449 (0.081 to 2.477)	0.3580
Critical sepsis	4.950 (1.166 to 21.016)	**0.0302**	4.950 (1.166 to 21.016)	**0.0302**	2.915 (0.527 to 16.116)	0.2202
Sex						
Male	Ref (S2.9.2)		Ref (S2.9.3)		Ref	
Female	7.164 (1.817 to 28.247)	**0.0049**	9.788 (2.192 to 43.697)	**0.0028**	8.300 (1.726 to 39.921)	**0.0083**
Obesity						
No	Ref (S2.9.4)		Ref (S2.9.5)		Ref	
Yes	7.632 (1.800 to 32.356)	**0.0058**	7.133 (1.630 to 31.217)	**0.0091**	3.231 (0.664 to 15.718)	0.1462
Smoking status						
No	Ref (S2.9.6)		Ref (S2.9.7)		Ref	
Smoke (days)	0.688 (0.207 to 2.284)	0.5415	0.728 (0.215 to 2.471)	0.6109	1.085 (0.277 to 4.252)	0.9067
Duration symptoms						
Less than 1 week	Ref (S2.9.8)		Ref (S2.9.9)		Ref	
More than 1 week	0.692 (0.216 to 2.218)	0.5358	0.604 (0.183 to 1.993)	0.4081	0.888 (0.223 to 3.541)	0.8666
Ethnicity						
White	Ref (S2.9.10)		Ref (S2.9.11)		Ref	
Non-white	2.404 (0.602 to 9.606)	0.2145	1.798 (0.445 to 7.253)	0.4100	1.763 (0.359 to 8.668)	0.4851
Vaccination						
No	Ref (S2.9.12)		Ref (S2.9.13)		Ref	
Yes	0.435 (0.082 to 2.309)	0.3286	0.548 (0.099 to 3.043)	0.4913	1.139 (0.118 to 11.014)	0.9104
Wave						
First	Ref (S2.9.14)		Ref (S2.9.15)		Ref	
Second	1.068 (0.271 to 4.208)	0.9249	1.120 (0.274 to 4.567)	0.8749	0.956 (0.176 to 5.200)	0.9589
Third	0.204 (0.034 to 1.213)	0.0805	0.264 (0.040 to 1.747)	0.1674	0.206 (0.016 to 2.613)	0.2230
Age						
<40 years	Ref (S2.9.16)		Ref (S2.9.17)		Ref	
40-60 years	0.620 (0.122 to 3.144)	0.5636	0.661 (0.128 to 3.407)	0.6211	0.307 (0.043 to 2.191)	0.2389
>60 years	0.366 (0.063 to 2.133)	0.2640	0.464 (0.079 to 2.740)	0.3969	0.200 (0.021 to 1.915)	0.1627
Clustering symptoms						
Cluster 1	Ref (S2.9.18)		Ref (S2.9.19)		Ref	
Cluster 2	2.240 (0.686 to 7.318)	0.1817	1.458 (0.432 to 4.922)	0.5433	1.658 (0.391 to 7.025)	0.4926
Clustering comorbidities						
Cluster 1	Ref (S2.9.20)		Ref (S2.9.21)		Ref	
Cluster 2	0.201 (0.055 to 0.733)	**0.0151**	0.187 (0.050 to 0.693)	**0.0121**	0.231(0.051 to 1.047)	0.0573

Defining the relationship between pre-admission risk factors and recovery from COVID-19 over time up to 12 months after admission with and without controlling for COVID-19 Severity Score and other potential confounders.

OR estimates and 95% CIs, along with the raw p value from the corresponding Wald z-test, are presented crude associations (left-hand column), conditional on severity (centre column) and fully adjusted logistic regression (right-hand column). These analyses aim to predict the presence or absence of the clinical endpoint of interest controlling for severity alone and controlling for severity plus additional potential confounders.

Crude=single-predictor model (factor alone); conditional on severity=factor adjusted for WHO severity only; adjusted ‘comprehensive’=all listed predictors mutually adjusted. Reference-level cells link (S2.k.m) to the supplementary table holding that model’s full coefficients including intercept. Significant p values (<0.05) in bold and highlighted.

* Acute Respiratory Distress Syndrome (ARDS).

To make the underlying estimates fully transparent and to facilitate these comparisons, we additionally report the complete coefficients of every model underlying tables 1–9) in a new set of supplementary tables (online supplemental tables S2.1.1 to S2.9.22).

### Trajectory of recovery from COVID-19 to 12 months after hospital discharge

**Figure 1:**
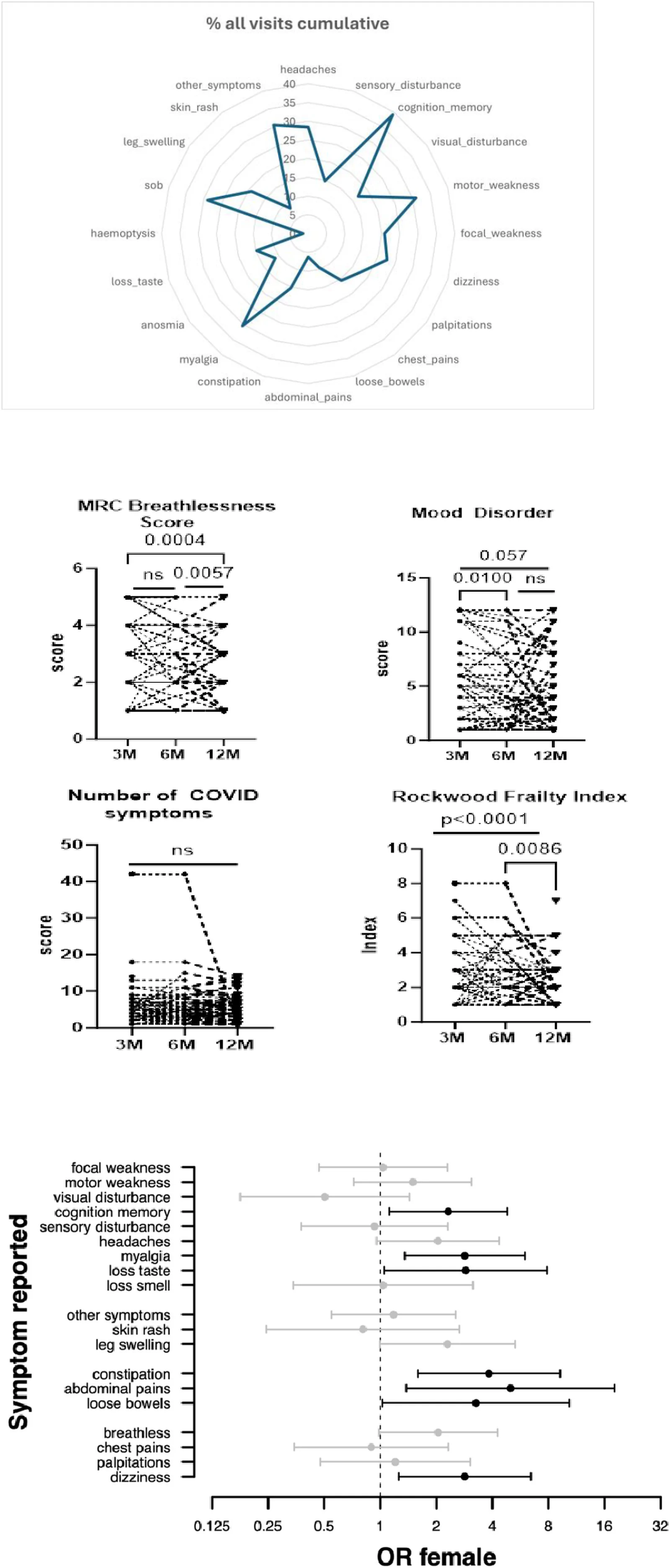
Prevalence of persisting COVID symptoms in recovery. Symptom prevalence. Spider plot showing the relative frequency (%) of the presence of each clinical endpoint of interest in the study cohort. Trajectory of recovery over time. Patients’ trajectories (lines) as a function of time (x-axes) for different clinical endpoints (plots) are shown by longitudinal analysis over time (Wilcoxon two-tailed). P values of paired t-tests (on the linear scale) are indicated for each combination of time points. COVID-19 symptoms by gender 12 months after symptom onset. OR (x-axis) is provided related to the reporting persistent symptoms by gender (y-axis), where OR estimates and CIs are respectively denoted by a dot and a segment, which are colour-coded in black in grey and black; black when the OR is different from 1 at the 5% type I error level*. Median time since symptom onset was 376 days (95% CI: 360 to 398 days). Women more likely to suffer with persistent COVID-19 symptoms: specifically, cognitive problems (p=0.024), myalgia (p=0.006), loss of taste (p=0.039), gastrointestinal symptoms (abdominal pain p=0.014, constipation p=0.003, loose stool p=0.044* and dizziness (p=0.012).

The trajectory of recovery from COVID following hospital discharge was assessed by comparing four endpoints over time: MRC Breathlessness Score, Mood Disorder Score, number of COVID symptoms and Rockwood Frailty Index, collected at 3, 6 and 12 months. Wilcoxon two-tailed t-tests of paired data were used to test for change in symptoms over each period (figure 1).

### Persistence of COVID-19 symptoms up to 12 months by gender

The OR with 95% CI of reporting persistent COVID-19 symptoms at the 12-month follow-up visit by gender was determined Wald z-tests of univariate logistic regression, with p value generated for difference in symptom presence between gender (figure 1).

## Results

### Recruitment demographic

A detailed description of the clinical and demographic characteristics of the overall patient cohort by COVID-19 disease severity score on admission is provided in online supplemental tables S1.1 and S1.2. 425 patients were recruited to the study across the first three waves of the COVID-19 pandemic. The median age of the cohort was 53 years (95% CI 51 to 56 years). 66% of patients recruited were male and of white ethnicity. 65% of individuals recruited had at least 1 comorbidity of which hypertension dominated (33% of all individuals); 27% had 1 and 38% 2 or more comorbidities. 29% of individuals were obese, of whom one-third had a (body mass index)>40. 405 patients were recruited in hospital (95% of the patient cohort), of whom 88% were admitted for management of COVID-19, 2% with acute cardiac event and 10% for other reasons including acute medical review (3%) or surgical procedure (3.5%). 47% of patients were admitted to the study recruitment hospital from another hospital, <1% from a care home and 48% of patients were admitted from home (data not shown). 5% patients were recruited in the community during wave 3 of the pandemic. Using date of symptom onset to assign ‘wave’ and thus likely SARS-CoV-2 variant causing disease, 21% of patients were admitted in wave 1, 50% in wave 2 and 29% in wave 3 of the COVID-19 pandemic. Disease severity varied and was dominated by patients with severe COVID-19 disease accounting for 60% of the cohort. We show that multiple demographic factors varied significantly by disease severity score on admission including age, gender, obesity, ethnicity, premorbid conditions such as heart disease and taking immunosuppressive treatment, reason for admission, duration of preadmission symptoms, wave of the epidemic at admission, vaccination status, number of doses and vaccine type received. 17% of patients had received at least one vaccination at presentation, predominantly in wave 3 of the pandemic (online supplemental table S1.2) of whom 27% were admitted with acute respiratory distress syndrome (ARDS) or critical sepsis.

### Acute clinical course and outcome of hospitalised patients

**Figure 2:**
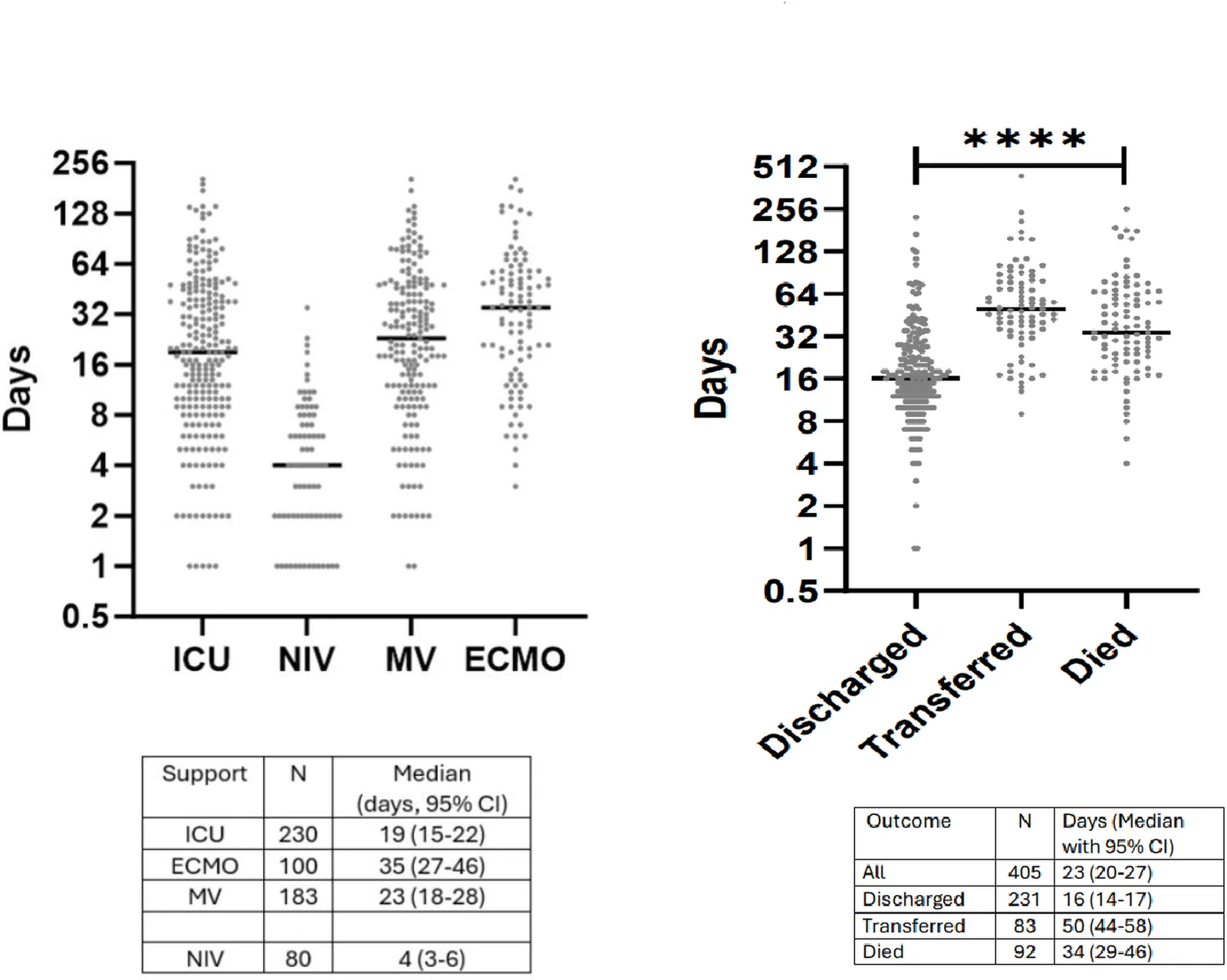
Acute clinical course of hospitalised patients. Duration of acute support by treatment modality. Dot plot showing the number of days (y-axis, log2 scale) by clinical pathway (number of days in intensive care unit (ICU) and duration of respiratory support by support modality (non-invasive ventilation (NIV), mechanical ventilation (MV) and extracorporeal-membrane oxygenation (ECMO), x-axis) per patient (dots). The horizontal bar displays the median. The sample size, median estimate and 95% CI for each clinical pathway are also given. Time from symptom onset to outcome of admission. Dot plot showing the number of days from the onset of symptoms to outcome of hospital admission (y-axis, log2 scale) per patient (dots), overall and according to outcome: discharge, transfer to another hospital for de-escalation of care and death (x-axis). The horizontal bar displays the median. The sample size, median estimate, and 95% CI for each outcome are indicated in the table below. The p value of a test for equality of medians is also indicated.

The clinical course of the acute phase of COVID-19 for patients recruited in hospital (n=405) is summarised in table 10 including the type of respiratory support given (many patients progressing through modality), clinical trial and adjunctive treatments, complications and outcome of admission. In brief, 86% of the cohort received respiratory support and over 50% of the cohort were admitted to the intensive care unit (ICU) with 21% of cohort receiving extracorporeal membrane oxygenation (ECMO) therapy (table 10). 37% patients were recruited to clinical trials and 17% randomised to treatment arms, which included convalescent plasma, biologics and antivirals. Adjunctive treatments were given to at least 24% of patients including haemofiltration, plasmapheresis and CytoSorb. The median duration of ICU stay and ventilatory support by modality is shown in figure 2, noting that duration of ICU stay and ventilatory support was widely spread with some patients remaining on ECMO for many months. Most of the cohort was discharged to home or for de-escalation of care to local hospital trust, however 22% of patients died in hospital (table 10), including eight patients (~2% of the cohort) who had been prevaccinated in accordance with high risk of severe disease status. Admission duration varied from 1 to over 128 days. Median length of stay was greatest in those who were ultimately transferred for de-escalation of care, (50 days) (figure 2).

**Table 10:** Clinical Course of Acute Admission and Outcome of hospitalised patients

Complications		Medical management	N=	%
Medical support		ICU admission	232	54.6
Respiratory support		Respiratory support	365	85.9
		Oxygen via cannula or mask	99	23.3
		High flow nasal oxygen	28	6.6
		NIV	41	9.6
		MV	86	20.2
		ECMO	89	20.9
Treatment trial		Trial treatment enrolled	158	37.2
		Treatment arm	70	16.5
		Standard of care	88	20.7
		Convalescent plasma	11	2.6
		Convalescent plasma and colchicine	3	0.7
		Convalescent plasma and aspirin and colchicine	2	0.5
		Convalescent plasma and aspirin and simvastatin	1	0.2
		Regeneron	3	0.7
	mAb antiviral	Regeneron and colchicine	1	0.2
		Regeneron and aspirin	5	1.2
		Regeneron and Baricitinib	1	0.2
		Baricitinib	6	1.4
	mAb anti-inflammatory	Tocilizumab	3	0.7
		Tocilizumab and colchicine	2	0.5
		Tocilizumab and hydroxychloroquine	2	0.5
		Rovelizumab	1	0.2
		aspirin	4	0.9
	other	aspirin and colchicine	3	0.7
		colchicine	1	0.2
		azithromycin	1	0.2
		hydroxychloroquine	11	2.6
		dexamethasone	13	3.1
	steroid	dexamethasone and remdesivir	1	0.2
	antiviral	lopinavir ritonavir	1	0.2
	At discharge	HEAL-COVID	4	0.9
Adjunctive treatment		haemofiltration	103	24.2
		plasmapheresis	16	3.8
		CytoSorb	23	5.4
Complications		thrombotic events	76	17.9
		cardiac events	36	8.5
		neurological events	40	9.4
		renal complications	71	16.7
		pneumothorax	24	5.6
		secondary bacterial pneumonia	25	5.9
Outcome		Discharge to home	230	54.1
		Transfer for de-escalation of care	83	19.5
		Death	92	21.6

Number of patients requiring intensive care unit (ICU) admission, respiratory support including mode of support, enrolment in treatment trials and treatments given, complications of admission, and outcome of admission are given.

ECMO, Extracorporeal Membrane Oxygenation; MV, Mechanical ventilation; NIV, Non-invasive ventilation.

Thrombotic, neurological, renal, cardiac events and pneumothorax were reported in several patients, with thrombosis and renal failure dominated reported in 18% and 17% of patients, respectively (table 10). These complications were mainly seen in patients with high disease severity score. The OR of these complications (except for cardiac event, table 4) increased in patients with critical sepsis, (Severity Score 5 and 6) (thrombotic events OR 21.4 (95% CI 2.6 to 177.7), p=0.005, neurological events OR 16.0 (95% CI 4.4, 57.7), p<0.001, renal events OR 19.7 (95% CI 7.6 to 51.1), p<0.001, pneumothorax OR 4.2 (95% CI 1.2 to 14.8), p=0.002) (tables 1–3 and 5), respectively). These increases are in comparison to a reference group of patients of lower severity, where the exact level of severity of the reference group varies depending on the complication. Certain demographic factors modified the point estimate of the complication risk although few reached significance. The relationship between the 11 most common symptoms of COVID and complications of admission was explored with symptoms first clustered into two main groups (online supplemental figure S2) and then evaluated to determine whether cluster membership may be an independent predictor of acute clinical course additional to COVID severity score on admission. Symptom cluster 2, a respiratory dominated symptom cluster, associated with increased risk of thrombosis (OR 2.7 (95% CI 1.2 to 6.1), p=0.021) and renal complications (OR 3.3 (95% CI 1.4 to 8.0), p=0.008) (tables 1–3); admission in wave 1 (compared with wave 2 but not wave 3) with increased risk of renal complications (OR 3.4 (95% CI 1.5 to 7.5), p=0.003); and symptoms for less than 1 week before admission with increased risk of pneumothorax (OR 3.1 (95% CI 1.2 to 8.3), p=0.025) (table 5). When adjusting for individual risk factors and severity only, younger age (<40 vs >60 years) was associated with increased probability of neurological events (OR 5.4 (95% CI 1.2 to 23.8), p=0.026) and pneumothorax (OR 10.10 (95% CI 1.08 to 90.91), p=0.043)

In the cohort overall, the OR of being alive at discharge (logistic regression model) was reduced in patients with the most severe disease and this association remained when adjusted for ethnicity, age and comorbidities as well as all other covariates recorded (OR 0.04 (95% CI 0.01 to 0.13), p<0.001) (table 6). In the time to event analysis, the hazard ratio of death was increased in patients with the most severe disease compared with patients with mild or moderate disease (HR 30.9 (95% CI 3.1 to 305.6), p=0.003) (table 7). Comorbidities cluster 1 was also associated with significantly increased hazard of death (HR 33.3 (95% CI 3.1 to 333.3), p=0.004) (table 7). While there was no association between vaccination status or time to admission and survival time in the individual analyses, associations were found once other risk factors were taken into account in the comprehensive analysis: specifically, positive vaccination status (HR 6.4 (95% CI 1.2 to 33.0), p=0.028) and shorter time to admission (less vs greater than 1 week) (HR 5.6 (95% CI 1.3 to 24.4), p=0.023) were associated with poorer survival (table 7).

### Clinical recovery to 12-month postadmission

433 follow-up visits were made by 231 patients (69% of the surviving to discharge cohort) at: 3 months (N=183), 6 months (N=131) and 12 months (N=128) following hospital discharge (or recruitment visit for patients recruited in the community). 68 Patients had one visit, 115 had two visits and 48 had three visits. Admission to discharge CRFs were matched with follow-up CRFs to evaluate recovery from COVID and how recovery related to the severity of initial COVID presentation and the demographic of the cohort. Results are shown in figure 1. There was no difference in the age or gender demographic of the cohort from admission to those attending each follow-up visit, however patients with the most severe COVID-19 were less well represented in the earlier follow visits (65% at 12 months, 49% at 3 and 6 months, p=0.04) (data not shown).

Of those patients attending follow-up, persisting COVID-19-related symptoms were reported in over 80% of individuals with neurocognitive and neuromuscular problems and breathlessness most reported (figure 1, 40%, 30% and 30%, respectively). Over 50% of patients reported at least three symptoms that they related to their COVID-19 infection with over 40% of individuals reporting symptoms consistent with a degree of mood disorder (psychological well-being score >4 by CRF where 1=‘not at all’, 2=several days, 3=more than half the days and 4=nearly every day for each of the four parameters assessed). Prevalence of symptoms differed over time since discharge for some but not all parameters. The demographic of the cohorts varied by follow-up visit and the number of follow-up visits by each patient was variable, potentially confounding interpretation of trajectory of recovery over time. A matched pair analysis for 3–6M visits (N=100) and 3–12M visits (N=90) was performed as is shown in figure 1. A significant improvement in MRC breathlessness and Rockwood frailty index was seen by 12 months postdischarge, however there was no significant change in mood disorder score and number of COVID-19 symptoms reported.

Women were more likely to report persistent COVID-19 symptoms in 12-month follow-up. The OR for many symptoms was greater in women than men and this reached significance for symptoms of myalgia (OR 2.8 (95% CI 1.4 to 6.0), p=0.006), cognitive problems (OR 2.3 (95% CI 1.1 to 4.8), p=0.024), loss of taste (OR 2.9 (95% CI 1.1 to 7.9), p=0.039), gastrointestinal symptoms of constipation (OR 3.8 (95% CI 1.6 to 9.2), p=0.003), loose stools (OR 3.3 (95% CI 1.0 to 10.4), p=0.045), abdominal pain (OR 5.0 (95% CI 1.4 to 18.1), p=0.014) and dizziness (OR, 2.8 (95% CI 1.3 to 6.4), p=0.012) (figure 1). There was no significant difference in COVID-19 severity score at recruitment by gender in this follow-up cohort (p=0.33, data not shown).

To determine the relationship between acute COVID-19 severity score and demographic factors that may predispose patients to persistent symptoms of breathlessness and of mood disorder, we generated the adjusted ORs for symptoms. We found that female gender (OR 99.8 (95% CI 2.4 to 4123.1), p=0.015) and critical sepsis (compared with mild/moderate/severe COVID-19) (OR 87.8 (95% CI 1.5 to 5103.0), p=0.031) were significantly associated with breathlessness in crude, conditional and comprehensive analysis (table 8). Obesity also associated with persistent breathlessness, however, significance was not sustained when adjusting for COVID-19 severity. Comorbidity cluster 1 was associated with persistent breathlessness on crude and conditional analysis, however this association did not persist in the comprehensive analysis.

Critical sepsis, female gender, obesity and comorbidity cluster 1 were also significantly associated with mood disorder in crude and conditional analysis. However, in the comprehensive analysis, female gender was the only significant association (OR 8.3 (95% CI 1.7 to 39.9), p=0.008) (table 9).

## Discussion

This study describes the clinical course of a cohort of patients with predominantly severe COVID-19 (including 89 patients treated with ECMO) through the first three waves of the COVID-19 pandemic in England from preadmission symptoms to recovery to 12 months and identifies the demographic factors associated with complications of acute disease, survival and persistent COVID-19 symptoms. Our cohort was more severe than in many published studies with nearly 60% requiring intensive care and 21% receiving ECMO. We found that the acute course was often prolonged extending to months in many individuals and complicated predominantly by thrombotic and renal events. We, as others have reported, found that the distribution of disease severity differs by age, gender, obesity, ethnicity, heart disease, immune suppressant medication, admission reason, wave of admission, vaccine received and vaccination status (online supplemental table S1.1).

While most inpatients survived to discharge, 22% of the cohort died. COVID-19 disease severity at admission was the dominant risk factor for four of the five major complications reported: thrombotic events, neurological events, renal complications and pneumothorax and with survival (tables 1–3, 5–7), however certain demographic and preadmission symptoms associated with acute complications and the association was sustained when COVID-19 severity score (conditional) and covariates were taken into account (comprehensive analysis). Renal complications were also associated with obesity (in the *comprehensive*’ model) and with admission in the first wave (compared with second wave) of the pandemic.

COVID is known to be associated with cardiovascular events including arrhythmic, myocarditis and acute thrombosis, of which thrombosis is most reported. The risk is highest in the acute phase. Thrombosis was the most common acute complication reported in our cohort, disproportionately represented by patients with critical sepsis and associated with symptom cluster 2 (respiratory dominant symptoms). Acute respiratory failure is the main complication of severe COVID and ARDS is in part attributed to coagulopathy. Acute COVID infection has been shown to cause micro thrombotic and thromboembolic disease in the lung with many patients showing evidence of coagulopathy on admission to hospital. Multiple mechanisms contribute to this, including direct viral activation of the endothelium and uncontrolled activation of the complement and inflammatory response pathways.^30^ The use of routine anticoagulation therapy for all hospitalised patients to reduce thrombotic risk and improve outcome has been explored and a Cochrane review reported in 2023 that all-cause mortality and thrombotic event (deep vein thrombosis or pulmonary embolism) were reduced in patients hospitalised with COVID treated with anticoagulation on admission; however, certainty of the evidence grading was considered very low for all parameters due to risk of critical bias in the studies reviewed^31^ and risk of bleeding in the treated cohort was also increased. An additional Cochrane report on the potential benefits and harms of prophylactic anticoagulants in non-hospitalised patients with COVID reported little or no difference in risk of venous thromboembolism, hospitalisation or adverse events; however, the evidence was again reported as of low certainty,^32^ emphasising the challenge of generating a robust evidence base to inform clinical practice.

Cardiac complications were reported in 9% of our cohort and did not associate either with COVID-19 severity score or our patient demographic. This contrasts with a careful systematic review of cardiovascular disease in COVID, which reports that COVID disease severity is associated with increased risk of pulmonary embolism, ischaemic stroke and myocardial infarction.^33^ In this study report, a gender bias in risk was found with pulmonary embolus being more common in men and myocardial infarction in women; the impact of age was less clear. Inadequate size effect may explain the discrepancy in study results; however, other confounding variables may be relevant. We did not differentiate patients admitted presenting with an acute cardiac event and those admitted with COVID respiratory disease who subsequently developed cardiac complications and as such our study may not have the granularity to identify demographic risk factors associated with cardiovascular complications of COVID with confidence.

Neurological complications were reported in 9% of our cohort and while the adjusted OR for neurological events increased a little when demographic factors were considered, we found no independent risk association of demographic factors. This may reflect the power of our study to detect effect noting that meta-analysis including larger cohorts have reported ischaemic stroke to have a prevalence of ≈2%^34^ in hospitalised patients increasing with comorbidities including hypertension, hyperlipidaemia and diabetes and with male gender. We did not differentiate between haemorrhagic and ischaemic stroke noting that both occurred in our cohort. There is little data reported on risk association with haemorrhagic stroke and identifying demographic/preadmission predictor of risk is complicated by inpatient care; anticoagulant treatment is routinely used to protect against the more common thrombotic complication of pulmonary embolism.

Chronic renal disease was identified as a significant preadmission risk factor in the early studies reporting outcomes of hospitalisation with COVID;^1 2^ however, in our cohort, relatively few patients were reported to have renal impairment at admission. 17% of patients developed acute renal complications during admission and while COVID severity score was the dominant risk factor, obesity, admission in wave 1 and respiratory dominant symptom cluster were associated.

The association of obesity with increased risk of acute renal complications has been reported by others. Page-Wilson *et al* also found a slight increased OR of obesity associating with acute renal injury.^35^ The reasons for this association are likely to be multifactorial including premorbid renal impairment and altered regulation of the immune response to SARS-CoV-2.^36^ Obesity may associate with a heightened preadmission pro-inflammatory state as determined by urokinase plasminogen activator receptor (suPAR) concentration, which has been reported to be a key mediator between obesity and poor outcome from COVID-19.^37^ Detailed serum cytokine analysis of patients with acute severe COVID has shown distinct inflammatory profiles between obese and non-obese patients segregating with higher prevalence of liver/kidney and lung damage, respectively.^38^ In addition, there may be a direct association with glomerular damage and the metabolome associated with obesity. It has been shown that SARS-CoV-2 causes a direct glomerular injury, collapsing glomerulopathy (CG) and that CGCG is strongly associated with apolipoprotein L1 risk variants, which themselves are associated with obesity risk.^39^

Being admitted in the first wave of the pandemic compared with the second wave was also associated with increased renal complications; however, there was no difference between first and third wave, despite third wave patients generally having milder disease (online supplemental table S1.2). Higher risk of renal complication in wave 1 compared with wave 2 may reflect a combination of the learning and changes in medical management of patients with severe COVID-19 as the pandemic evolved. Early reports from China suggested that there was no risk of acute kidney injury (AKI) in patients hospitalised with COVID;^40^ however, subsequent studies from Europe and the USA reported a greatly increased risk with meta-analyses reporting renal complications in 5%–40% of patient hospitalised with COVID.^41^ This reduction in risk of AKI over the first two waves that we report may reflect the increased awareness of risk of AKI and changes in fluid management policy in subsequent waves that reduced the risk of AKI over time, noting that others have reported reducing incidence of AKI over time in wave 2 of the UK pandemic in two London hospitals.^42^ The similar levels of renal complications in wave 3 patients compared with wave 1 is difficult to explain and may be due to inadequate power in the smaller wave 3 cohort to test for wave 3 as an independent confounding variable.

In our study, 6% of our hospitalised cohort developed pneumothorax. This risk was increased in patients with the most severe admission score and more rapid progression from symptom onset to admission. While the prevalence of pneumothorax in patients hospitalised with COVID is reported to be relatively low at 0.3%, it is known to be much greater in patients with severe disease requiring ventilatory support. The association of pneumothorax with shorter time to admission likely reflects a more aggressive disease course to needing ventilatory support, a key admission requirement at the time of the study. Notably, pneumothorax did not associate with premorbid lung disease, as previously noted,^43^ and we found no association with gender or age consistent with previous reports that the increased prevalence of pneumothorax in males may be attributed to more severe COVID-19 disease.^44^

We used two types of analysis, logistic regression and survival analysis, to evaluate preadmission factors associated with survival to discharge, to examine survival at time of discharge and time to death, respectively. In the first analysis, increased COVID severity (critical sepsis), older age (>60 years vs <40 years old), presence of comorbidities and being of unassigned ethnicity impacted was associated with increased probability of death before discharge. The latter highlights the deficit in ethnicity recording in medical records and the challenge of assigning correct ethnicity in the intensive care setting. The effect of missingness in the ethnicity variable was mitigated by treating it as a three-level factor, with ‘unknown’ as an additional level alongside ‘white’ and ‘non-white’ and using this factor in regression-type analyses. In the second analysis, increased COVID severity (critical sepsis) and presence of comorbidities are associated with increased hazard of death. So too are positive vaccination status and shorter duration of symptoms (<1 week) prior to admission (in the ‘*comprehensive*’ analysis). In contrast to the first analysis (logistic regression), which examines probability of death before discharge, this statistical approach (survival analysis) compares risk of death at any point and accounts for each patient’s length of time in the study. These data corroborate other studies including the large multicentre ISARC-4C study by Docherty *et al* reporting that patients of older age, male gender, with comorbidities were less likely to survive hospitalisation.^45^

Of our results, the impact of the wave of the pandemic and vaccination status on survival and time to death warrants some discussion. While viral variants (and virulence) evolved through successive waves of the pandemic, medical preparedness and management also evolved and the seasons were distinct presenting multiple potential confounding variables contributing to this association of wave and mortality. Vaccination at the time of the study, except for the 2 patients in a vaccine trial, was selectively offered to patients at high risk of disease. As such, our data suggest that vaccine failure in high-risk patients associated with shorter time to death in this cohort, which may be understood in that this cohort had been predefined as vulnerable to death from COVID. These data emphasise that while new variants of COVID have different virulence patterns and vaccination largely protects against severe infection, for patients who require hospitalisation for COVID, mortality remains high. Detailed serological studies are in process to determine whether those individuals vaccinated prior to admission had primary or secondary vaccine failure.

### Recovery

Patients were invited for follow-up at regular intervals to 12 months postdischarge and most patients attended at least one follow-up visit with attending at 12 months postdischarge. Multiple COVID-19 symptoms persisted in many patients and while breathlessness improved by 12 months postdischarge, symptoms of mood disorder did not. In contrast to acute complications, breathlessness was the only symptom associating with COVID-19 acute disease severity score. However female gender was also associated with persistent breathlessness, and this was at a similar OR to, and independent of COVID-19 disease severity on admission. Female gender also associated with persistent symptoms of mood disorder and with neurological and abdominal symptoms. Gender was the only demographic factor associated independently with recovery.

These findings corroborate the numerous case series and systematic reviews now published including a large multicentre UK study,^46^ showing that life-changing sequelae of COVID are common, complex, unrelated to acute disease severity, predominantly gender-biased and may not improve at least over the first year after hospital discharge. Women are reported to be at increased risk of several neurological symptoms including brain fog, fatigue and depression with a suggestion of some improvement at a year although few studies are reported that extended to or beyond this time frame.^47^ Multiple mechanisms have been proposed including the propensity of women to chronic inflammatory/autoimmune diseases.^48^ Neuroinflammation is reported in acute infection^49 50^ and delayed resolution may explain some of the symptoms reported such as cognitive function and depression. Autonomic dysregulation is also widely reported, which may present with cardiovascular symptoms, such as dizziness, palpitations, temperature instability and fatigue and may exacerbate symptoms of depression and impaired cognition.^51 52^ Autonomic mechanism may also explain the persistent gastrointestinal symptoms reported more commonly in women after acute COVID infection. Indirect risk factors such as chronic stress, deconditioning, perception of pain and psychosocial factors including carer responsibilities also have a gender bias that may impact on symptom perception and burden in women.

Our study, although multicentre and prospective, was relatively small and biased to patients with very severe COVID, many requiring prolonged intensive care. The study design adapted to clinical and practical constraints in implementation as the pandemic evolved and it was focused on identifying clinical correlates of disease severity in our cohort to be considered in serological studies exploring the humoral immune correlates of protection. As such, this might limit the generalisability of our findings as applied to all hospitalised patients. However, as systematic reviews and meta-analysis over the years since the pandemic have shown, results of studies such as ours are generally validated in larger more mixed cohorts from similar geographical areas. The prospective nature of our study, from preadmission symptoms, acute disease to recovery to 12 months with matched pair analysis provides some confidence in associating clinical course with demographic and preadmission risk factors; however, there may be more associations that the study was inadequately powered to identify. Similarly, while many potentially confounding variables were considered in our analysis, the therapies given during hospital stay were many and diverse such that it was not possible to analyse their contribution to outcome. Mortality following admission with COVID-19 may be under-represented as we did not evaluate mortality postdischarge, which has recently been reported to be 5% by systematic review and meta-analysis of long-term posthospital mortality.^53^

Identifying real independent associations of patient-reported symptoms with acute clinical course of disease is extremely challenging due to the high numbers of potentially confounding variables including the reliability of symptom reporting. The biological mechanisms that may explain the association we report of thrombotic and renal complications with patients who cluster to a narrower breadth of respiratory predominant symptoms likely reflect a bias in dominant symptom reporting by patients with most severe disease, noting that many of our patients were recruited in critical care with pre-COVID symptoms collated from medical records. Other studies have reported an association between COVID-related gastrointestinal disturbance and acute clinical course.^54–56^ We did not see this. No other preadmission symptoms have been found to predict risk of acute disease complications independent of COVID-19 disease severity score as far as we are aware.

While many patients at least one follow-up visit, a significant proportion of the cohort is not represented and as such there is a risk of ascertainment bias in the recovery analysis presented. However, it is unclear in which direction this bias may be noting some patients with more severe disabling symptoms may have felt unable to attend, particularly if they had a long distance to travel to the appointment. However, our results are consistent with what has been reported by other groups although for all such studies, those patients lost to follow-up present an important cohort for whom recovery may be different. In the last 12 months to November 2024 in the UK, an average of 322 patients are hospitalised with COVID each day and 8.5% of deaths have COVID listed as a contributing cause.^57^ This cohort of patients are mostly vaccinated (in accordance with national strategy), infected with further evolved variants of the SARS-CoV2 virus and have comorbidities more likely to be associated with both primary and secondary vaccine failure.^58 59^ As such, the applicability of our findings to patients currently hospitalised with severe COVID is unclear. Ongoing prospective multicentre studies are required to ensure that the extensive learning achieved in the early waves of the pandemic continues to evolve, remaining applicable in real time for this rapidly evolving disease.

## Supplementary material

10.1136/bmjopen-2025-109653 online supplemental figure 1

10.1136/bmjopen-2025-109653 online supplemental figure 2

10.1136/bmjopen-2025-109653 online supplemental table 1

10.1136/bmjopen-2025-109653 online supplemental table 2

## Data Availability

Data are available upon reasonable request.
