# Supplementary material for: Defining the preadmission factors that modify risk of acute complications, survival and long-term recovery from COVID-19: prospective, longitudinal, multisite, cohort study based in England
Source: BMJ Open. 2026 Sep 21;16(9):e109653. doi: 10.1136/bmjopen-2025-109653 (PMC13599834; doi:10.1136/bmjopen-2025-109653)
Supplement: Supplementary file 1 [file bmjopen-16-9-s001.pdf]

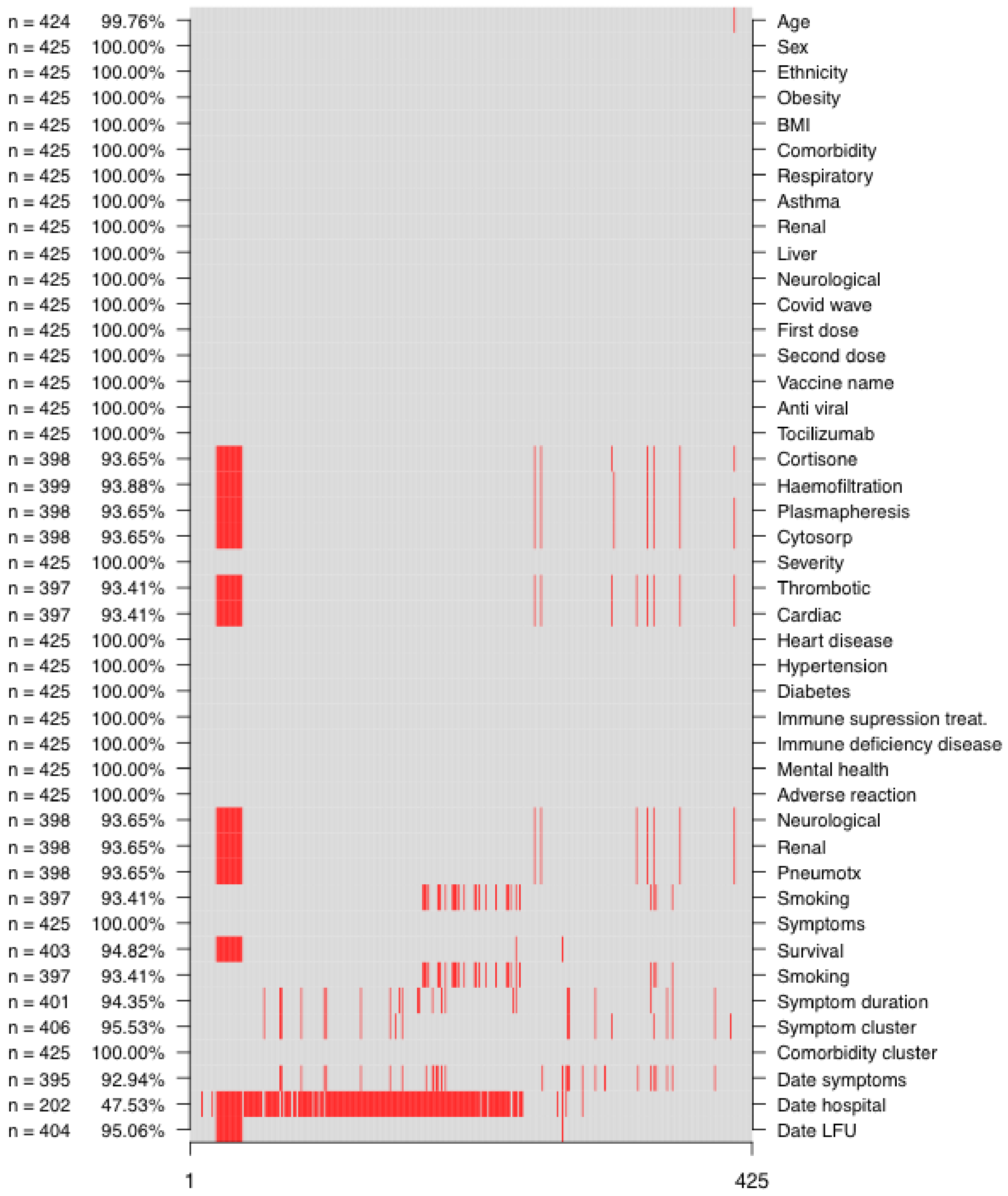

n = 424 99.76% Age
n = 425 100.00% Sex
n = 425 100.00% Ethnicity
n = 425 100.00% Obesity
n = 425 100.00% BMI
n = 425 100.00% Comorbidity
n = 425 100.00% Respiratory
n = 425 100.00% Asthma
n = 425 100.00% Renal
n = 425 100.00% Liver
n = 425 100.00% Neurological
n = 425 100.00% Covid wave
n = 425 100.00% First dose
n = 425 100.00% Second dose
n = 425 100.00% Vaccine name
n = 425 100.00% Anti viral
n = 425 100.00% Tocilizumab
n = 398 93.65% Cortisone
n = 399 93.88% Haemofiltration
n = 398 93.65% Plasmapheresis
n = 398 93.65% Cytosorp
n = 425 100.00% Severity
n = 397 93.41% Thrombotic
n = 397 93.41% Cardiac
n = 425 100.00% Heart disease
n = 425 100.00% Hypertension
n = 425 100.00% Diabetes
n = 425 100.00% Immune supression treat.
n = 425 100.00% Immune deficiency disease
n = 425 100.00% Mental health
n = 425 100.00% Adverse reaction
n = 398 93.65% Neurological
n = 398 93.65% Renal
n = 398 93.65% Pneumotx
n = 397 93.41% Smoking
n = 425 100.00% Symptoms
n = 403 94.82% Survival
n = 397 93.41% Smoking
n = 401 94.35% Symptom duration
n = 406 95.53% Symptom cluster
n = 425 100.00% Comorbidity cluster
n = 395 92.94% Date symptoms
n = 202 47.53% Date hospital
n = 404 95.06% Date LFU
1
425
