# Supplementary material for: Defining the preadmission factors that modify risk of acute complications, survival and long-term recovery from COVID-19: prospective, longitudinal, multisite, cohort study based in England
Source: BMJ Open. 2026 Sep 21;16(9):e109653. doi: 10.1136/bmjopen-2025-109653 (PMC13599834; doi:10.1136/bmjopen-2025-109653)
Supplement: Supplementary file 2 [file bmjopen-16-9-s002.pdf]

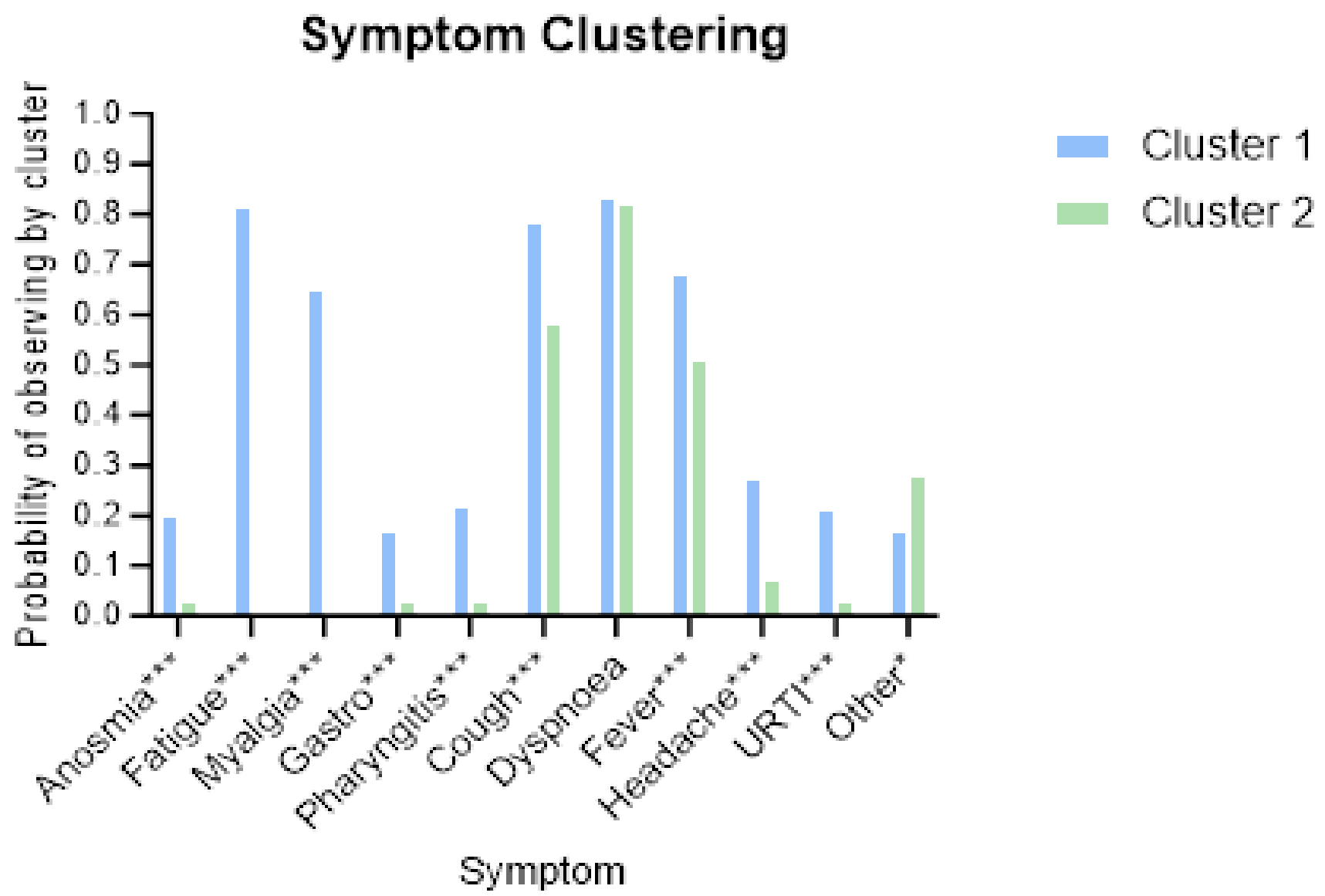

Symptom Clustering
Probability of observing by cluster
1.0
0.9
0.8
0.7
0.6
0.5
0.4
0.3
0.2
0.1
0.0
Cluster 1
Cluster 2
Anosmia***
Fatigue***
Myalgia***
Gastro***
Pharyngitis***
Cough***
Dyspnoea
Fever***
Headache***
URTI***
Other*
Symptom

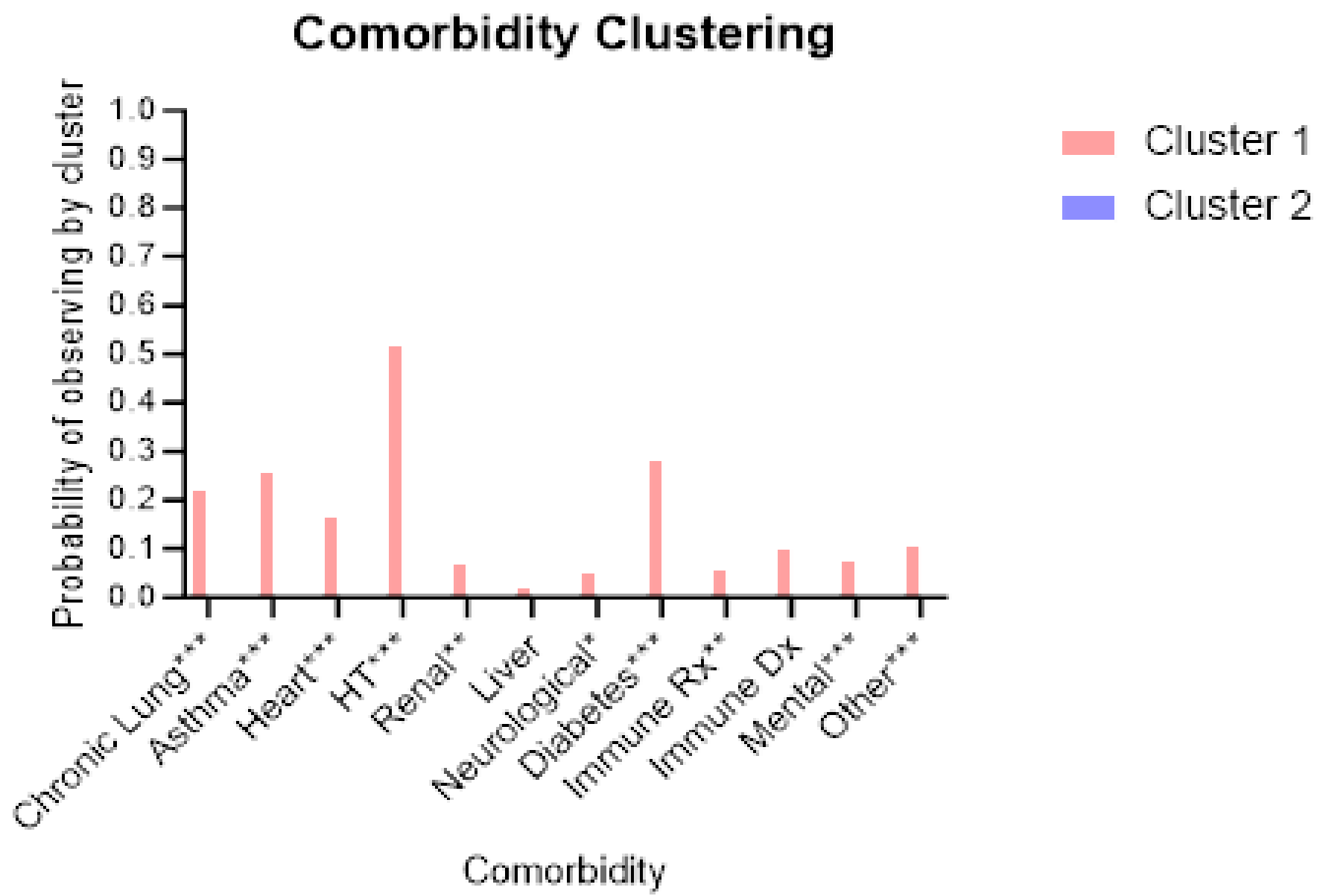

Comorbidity Clustering
Probability of observing by cluster
1.0
0.9
0.8
0.7
0.6
0.5
0.4
0.3
0.2
0.1
0.0
Cluster 1
Cluster 2
Chronic Lung***
Asthma***
Heart***
HT***
Renal**
Liver
Neurological*
Diabetes***
Immune Rx**
Immune Dx
Mental***
Other***
Comorbidity
