# Supplementary material for: Defining the preadmission factors that modify risk of acute complications, survival and long-term recovery from COVID-19: prospective, longitudinal, multisite, cohort study based in England
Source: BMJ Open. 2026 Sep 21;16(9):e109653. doi: 10.1136/bmjopen-2025-109653 (PMC13599834; doi:10.1136/bmjopen-2025-109653)
Supplement: Supplementary file 3 [file bmjopen-2025-109653.reviewer_comments.pdf]

PEER REVIEW HISTORY

BMJ Open publishes all reviews undertaken for accepted manuscripts. Reviewers are asked to complete a checklist review form and are provided with free text boxes to elaborate on their assessment. These free text comments are reproduced below.

ARTICLE DETAILS

Title (Provisional)

Defining the pre-admission factors that modify risk of acute complications, survival and long-term recovery from COVID-19: a prospective, longitudinal, multisite, cohort study based in England.

Authors

Baxendale, H E; Law, Martin; Matthews, Claire; Piggott, Janet; Vancheeswaran, Rama; Vuylsteke, Alain; Wilson, Catherine; Hopley, Erin; Toshner, Mark; Boubriak, Iryna; Newman, Joseph; Hannan, Nasir; Wilkinson, Alexander JK; Barlow, Andrew; Harriott, Nicole; Jones, Nicola; Webb, Stephen; Schwaeble, William; Heeney, Jonathan; Couturier, Dominique

---

VERSION 1 - REVIEW

**Reviewer**	**1**
**Name**	**Kelson, Zoe**
**Affiliation**	**University of Exeter, Mathematics**
**Date**	**01-Oct-2025**
**COI**	**None**

This study aims to identify the demographic factors and pre-admission symptoms impacting on COVID-19 disease severity, acute clinical course and long-term recovery to be considered in subsequent serological analysis.

Reviewer comments:

Can the authors please summarise the statistical methods applied in the Abstract?

"Preadmission symptom cluster and duration of symptoms associated with risk of thrombosis/renal disease and pneumothorax respectively. Renal complications were more likely to be seen in the first wave compared to the second wave of the pandemic. Survival to discharge was independently associated with ethnicity, age (<40yrs vs >60yrs) and absence of comorbidities however hazard of death was increased for all hospitalised patients who had received at least one COVID vaccine (as high-risk patient) pre-admission, had shorter time from symptom onset to admission, or had comorbidities. Most patients reported long term sequelae with neuromuscular and cognitive effects dominating early on and mood

disturbance and breathlessness persisted to 12 months. Whilst COVID-19 severity score was the dominant risk factor for persistent dyspnoea, gender was the dominant risk factor for persistent mood disorder and associated with a greater risk of persistent gastrointestinal and cognitive problems." [Abstract]

Can the authors please report statistical outcomes alongside and in support of study findings stated in the Abstract?

"prospective multicentre study in England, recruiting patients hospitalised with COVID-19 over the first 3 waves of the COVID-19 pandemic and following them through the acute phase of disease and in convalescence up to 1 year. Non-hospitalised patients were also recruited representing patients with asymptomatic or mild disease."

Can the authors please comment on whether the included cohort can be considered to be representative?

Can the authors please provide a discussion on sample size?

" Recruitment and Clinical Data were systematically recorded"

and

" pattern of missingness was analysed using a heatmap that displays the missingness status through a colour code"

Can the authors please comment on whether missing data can be considered to be missing at random?

Can the authors please clarify how missing data was handled in the analysis?

"analysed using contingency tables on which Fisher's tests of independence were performed. The p-value from the Fisher test was obtained through Monte Carlo simulation, considering 25,000 samples"

and

"Symptoms and comorbidities presence/absence data were separately clustered by partitioning participants around medoids... based on Jaccard dissimilarity measures... the probability of observing each symptom and comorbidity per cluster was compared using proportion tests"

and

"analysed using adjusted and unadjusted logistic regression with a logit link... Unadjusted analyses considered severity solely as an ordered categorical predictor (with mild as the reference group). Adjusted analyses controlled for sex, obesity, smoking status, duration of symptoms, ethnicity, vaccination status, COVID wave, age group, and symptom and comorbidity clusters. Odds ratio estimates and 95% confidence intervals, along with the corresponding Wald z-test, were calculated... survival analyses, specifically Cox regressions, are used as an alternative to the logistic regressions considered"

and

"adjusted and unadjusted generalised linear mixed models for binary endpoints, as fitted using the glmer function from the lme4 package with the family argument set to binomial, were used to analyse the effect of severity on two recovery endpoints—the MRC Breathlessness score ranged from 0-4 and was dichotomised into 0 (no breathlessness) to 1-4 (mild to severe breathlessness), and mood disorder from 0 (least disorder) to 16 based on 4 parameters (interest, feeling down, nervous and worrying) — measured at up to three time points per patient and was dichotomised into 0 vs >0. Details of the follow up questionnaires are found in Supplementary File S1.4. All models treated patients as random effects. Unadjusted analyses solely predicted the probability of success based on severity (a 3-level factor with mild as the reference group) and time (a factor with levels 3, 6, and 12 months, with 3 months as the reference group), while adjusted analyses further controlled for sex, obesity, smoking status, duration of symptoms, ethnicity, vaccination status, age group, and symptom and comorbidity clusters. Odds ratio estimates and 95% confidence intervals, along with the corresponding Wald z-test, were calculated, and p-values were not adjusted for multiplicity."

and

"The trajectory of recovery from COVID following hospital discharge was assessed by matched pair analysis of four endpoints: MRC Breathlessness Score, Mood Disorder Score, number of COVID symptoms and Rockwood Frailty Index, collected at 3,6 and 12 months. Wilcoxon two tailed t-tests of paired data was used to test for change in symptoms over each period"

and

"The odds ratio with 95% CI of reporting persistent COVID-19 symptoms at the 12-month follow up visit by gender was determined Wald z-tests of univariate logistic regression, with p value generated for difference in symptom reporting between gender"

Appropriate statistical and modelling methods have been applied.

Can statistical outcomes please be reported alongside and in support of stated study findings throughout the Results text?

"One of the limitations of the study is the COVID-19 disease severity bias to patients critically ill with COVID60 19. As such, the applicability of the findings to patients with milder disease is unclear. " [Strengths and Limitations of this Study]

and

"Our study, although multicentre and prospective, was relatively small and biased to patients with very severe COVID, many requiring prolonged intensive care. The study design adapted to clinical and practical constraints in implementation as the pandemic evolved and it was focused on identifying clinical correlates of disease severity in our cohort to be considered in

serological studies exploring the humoral immune correlates of protection. As such this might limit the generalisability of our findings as applied to all hospitalised patients. However as systematic reviews and meta-analysis over the years since the pandemic have shown, results of studies such as ours are generally validated in larger more mixed cohorts from similar geographical areas. The prospective nature of our study, from pre-admission symptoms, acute disease to recovery to 12 months with matched pair analysis provides some confidence in associating clinical course with demographic and pre-admission risk factors; however, there may be more associations that the study was inadequately powered to identify. Similarly, whilst many potentially confounding variables were considered in our analysis, the therapies given during hospital stay were many and diverse such that it was not possible to analyse their contribution to outcome. Mortality following admission with COVID-19 may be under-represented as we did not evaluate mortality post discharge which has recently been reported to be 5% by systemic review and metanalysis of longer-term post-hospital mortality {47}. "

The authors provide a discussion on the study limitations.

Can the authors please confirm if any reporting guidelines were adhered to, and provide a copy of the associated checklist?

**Reviewer**	**2**
**Name**	**Gómez Bravo, Raquel**
**Affiliation**	**Universite du Luxembourg, Institute for Health and Behaviour. Research Unite INSIDE**
**Date**	**30-Nov-2025**
**COI**	**None**

Dear Editor and Authors,

Thank you for the opportunity to review this manuscript addressing the long-term outcomes of patients hospitalised with COVID-19. The study presents important prospective data including detailed inpatient clinical profiles and structured follow-up extending to 12 months. The focus on individuals with severe and critical illness, many of whom required intensive care, fills an area of evidence that remains relatively under-represented.

There are nevertheless several substantive concerns. First, the cohort is heavily skewed towards the most severe forms of COVID-19, with a high rate of intensive care utilisation and inpatient mortality. This limits the broader applicability of the findings and should be made more explicit early in the manuscript. Demographic missingness is concentrated in the most severe subgroup, particularly for ethnicity, and the current text does not sufficiently describe how this was handled or what biases may result.

Second, the statistical analysis relies on a very large number of unadjusted hypothesis tests, raising the possibility of inflated false-positive findings. Greater caution is required in interpreting marginal associations, along with clearer justification for the analytic approach.

Third, the clustering analysis of acute symptoms and comorbidities is exploratory in nature. The binary input data, limited separation of clusters and absence of validation do not match the current standard in LC phenotyping. As such, these findings should be repositioned as preliminary observations and not used to imply mechanistic distinctions.

Regarding the reported sex differences in persistent symptoms, the finding that women experience higher burdens of breathlessness, fatigue and mood disturbance is consistent with the emerging LC literature. However, the manuscript does not adequately discuss the well-established biological and sociocultural factors underlying this pattern. There is a risk of oversimplification if female sex is presented without context or explanation. Expanding the discussion to recognise sex-specific immune and autonomic pathways, hormonal influences and social determinants of health would improve interpretability and avoid unintentional gender bias.

Furthermore, thrombotic complications represent one of the most frequent and clinically relevant acute events in this cohort, yet receive limited attention in the discussion. Given the central role of associated coagulopathy in patient outcomes and recovery, deeper examination of this finding would be valuable. Similarly, the persistence of symptoms such as dizziness, breathlessness and gastrointestinal issues raises the possibility of post-COVID autonomic dysfunction, including postural orthostatic tachycardia syndrome. Acknowledging this recognised mechanism of long-term morbidity would strengthen the clinical relevance of the results and align the manuscript with current scientific understanding.

The interpretation of vaccination as a risk factor for mortality requires clearer acknowledgment of confounding by indication. The use of pandemic waves as proxies for variant exposure should also be presented with greater caution. Considerable attrition in long-term follow-up introduces the potential for survivorship and non-response bias, which must be addressed when interpreting recovery trajectories.

Finally, improvements in clarity and presentation would enhance the manuscript's accessibility. The abstract should give greater emphasis to the study's limitations, and some tables may benefit from re-formatting to support readability.

In conclusion, this study contributes meaningful data on outcomes after life-threatening COVID-19. With revisions to strengthen analytical transparency, update the interpretive framework and expand discussion of key mechanisms, the manuscript would provide a clearer and more clinically informative contribution to the field.

Thank you again for the opportunity to review this work.

Sincerely

VERSION 1 - AUTHOR RESPONSE

Response to reviewer comments :

Reviewer: 1
Prof. Zoe Kelson, University of Exeter
Comments to the Author:

This study aims to identify the demographic factors and pre-admission symptoms impacting on COVID-19 disease severity, acute clinical course and long-term recovery to be considered in subsequent serological analysis.

Reviewer comments:

Can the authors please summarise the statistical methods applied in the Abstract?

- *We have now briefly summarised the statistical methods used, while keeping in mind the brevity required.*

"Preadmission symptom cluster and duration of symptoms associated with risk of thrombosis/renal disease and pneumothorax respectively. Renal complications were more likely to be seen in the first wave compared to the second wave of the pandemic. Survival to discharge was independently associated with ethnicity, age (<40yrs vs >60yrs) and absence of comorbidities however hazard of death was increased for all hospitalised patients who had received at least one COVID vaccine (as high-risk patient) pre-admission, had shorter time from symptom onset to admission, or had comorbidities. Most patients reported long term sequelae with neuromuscular and cognitive effects dominating early on and mood disturbance and breathlessness persisted to 12 months. Whilst COVID-19 severity score was the dominant risk factor for persistent dyspnoea, gender was the dominant risk factor for persistent mood disorder and associated with a greater risk of persistent gastrointestinal and cognitive problems." [Abstract]

Can the authors please report statistical outcomes alongside and in support of study findings stated in the Abstract?

- *Thank you. We have added point estimates, 95% confidence intervals and p values to the abstract (and throughout the manuscript) at the appropriate points.*

"prospective multicentre study in England, recruiting patients hospitalised with COVID-19 over the first 3 waves of the COVID-19 pandemic and following them through the acute phase of disease and in convalescence up to 1 year. Non-hospitalised patients were also recruited representing patients with asymptomatic or mild disease."

Can the authors please comment on whether the included cohort can be considered to be representative?

- *We consider the cohort to be bias to hospitalised patients with severe disease. In addition, the patients with mild disease were more likely to be recruited in the 3rd wave of the pandemic due to lock downs and practical difficulties associated with community recruitment. As such, we consider the cohort to be representative of patients with more severe disease admitted in wave 1 and 2 of the pandemic (ie predominantly before vaccination was introduced). We have discussed this bias in some detail in the discussion and it is included as a stated weakness of the study in 'strengths and weaknesses'.*

Can the authors please provide a discussion on sample size?

The study was an observational study, rather than a randomised or single-arm trial with an intervention, planned at the heat of the pandemic without much information on what was to be expected so that no sample size calculation was performed for this pragmatic study. We now state this in the manuscript, and also that study funding was awarded under the expectation of reaching 500 participants (Study Design and Approvals).

" Recruitment and Clinical Data were systematically recorded"
and
" pattern of missingness was analysed using a heatmap that displays the missingness status through a colour code"

Supplementary Figure S1 (now including improved labelling) displays the missingness status per patient (x-axis) and variable (y-axis): red if missing, grey if observed. We can note that, for many patients, the same variables are missing as a consequence, for example, being not relevant for a patient given their COVID severity.

Can the authors please clarify how missing data was handled in the analysis?

- *For each analysis, we performed complete-case analysis considering all patients without missing observations in the set of variables of interest. As the level of missingness was typically low (rarely more than 25 observations missing per variable out of 425 patients) and clustered (as explained in our previous answer), we expect the impact of missingness on results to be minimal. A notable exception is the variable 'Date of hospitalisation' which showed a large proportion of missing data corresponding to patients who were not hospitalised. This was handled by considering a Cox regression with both left truncation and right censoring (as explained in the manuscript).*

"analysed using contingency tables on which Fisher's tests of independence were performed. The p-value from the Fisher test was obtained through Monte Carlo simulation, considering 25,000 samples"
and
"Symptoms and comorbidities presence/absence data were separately clustered by partitioning participants around medoids... based on Jaccard dissimilarity measures... the probability of observing each symptom and comorbidity per cluster was compared using proportion tests"
and
"analysed using adjusted and unadjusted logistic regression with a logit link... Unadjusted analyses considered severity solely as an ordered categorical predictor (with mild as the reference group). Adjusted analyses controlled for sex, obesity, smoking status, duration of

symptoms, ethnicity, vaccination status, COVID wave, age group, and symptom and comorbidity clusters. Odds ratio estimates and 95% confidence intervals, along with the corresponding Wald z-test, were calculated… survival analyses, specifically Cox regressions, are used as an alternative to the logistic regressions considered"
and
"adjusted and unadjusted generalised linear mixed models for binary endpoints, as fitted using the glmer function from the lme4 package with the family argument set to binomial, were used to analyse the effect of severity on two recovery endpoints—the MRC Breathlessness score ranged from 0-4 and was dichotomised into 0 (no breathlessness) to 1-4 (mild to severe breathlessness), and mood disorder from 0 (least disorder) to 16 based on 4 parameters (interest, feeling down, nervous and worrying) — measured at up to three time points per patient and was dichotomised into 0 vs >0. Details of the follow up questionnaires are found in Supplementary File S1.4. All models treated patients as random effects. Unadjusted analyses solely predicted the probability of success based on severity (a 3-level factor with mild as the reference group) and time (a factor with levels 3, 6, and 12 months, with 3 months as the reference group), while adjusted analyses further controlled for sex, obesity, smoking status, duration of symptoms, ethnicity, vaccination status, age group, and symptom and comorbidity clusters. Odds ratio estimates and 95% confidence intervals, along with the corresponding Wald z-test, were calculated, and p-values were not adjusted for multiplicity."
and
"The trajectory of recovery from COVID following hospital discharge was assessed by matched pair analysis of four endpoints: MRC Breathlessness Score, Mood Disorder Score, number of COVID symptoms and Rockwood Frailty Index, collected at 3,6 and 12 months. Wilcoxon two tailed t-tests of paired data was used to test for change in symptoms over each period"
and
"The odds ratio with 95% CI of reporting persistent COVID-19 symptoms at the 12-month follow up visit by gender was determined Wald z-tests of univariate logistic regression, with p value generated for difference in symptom reporting between gender"
Appropriate statistical and modelling methods have been applied.

Can statistical outcomes please be reported alongside and in support of stated study findings throughout the Results text?

- *We have added point estimates, 95% confidence intervals and p values to stated study findings throughout the manuscript.*

"One of the limitations of the study is the COVID-19 disease severity bias to patients critically ill with COVID60 19. As such, the applicability of the findings to patients with milder disease is unclear. " [Strengths and Limitations of this Study]
and
"Our study, although multicentre and prospective, was relatively small and biased to patients with very severe COVID, many requiring prolonged intensive care. The study design adapted to clinical and practical constraints in implementation as the pandemic evolved and it was focused on identifying clinical correlates of disease severity in our cohort to be considered in serological studies exploring the humoral immune correlates of protection. As such this might limit the generalisability of our findings as applied to all hospitalised patients. However as systematic reviews and meta-analysis over the years since the pandemic have shown, results of studies such as ours are generally validated in larger more mixed cohorts from similar

geographical areas. The prospective nature of our study, from pre-admission symptoms, acute disease to recovery to 12 months with matched pair analysis provides some confidence in associating clinical course with demographic and pre-admission risk factors; however, there may be more associations that the study was inadequately powered to identify. Similarly, whilst many potentially confounding variables were considered in our analysis, the therapies given during hospital stay were many and diverse such that it was not possible to analyse their contribution to outcome. Mortality following admission with COVID-19 may be under-represented as we did not evaluate mortality post discharge which has recently been reported to be 5% by systemic review and metanalysis of longer-term post-hospital mortality {47}. "
The authors provide a discussion on the study limitations.

Can the authors please confirm if any reporting guidelines were adhered to, and provide a copy of the associated checklist?

- *Thank you for flagging this. We have adhered to the Strobe checklist guideline for reporting and provided a completed copy of this.*

Reviewer: 2
Dr. Raquel Gómez Bravo, Universite du Luxembourg
Comments to the Author:

Dear Editor and Authors,

Thank you for the opportunity to review this manuscript addressing the long-term outcomes of patients hospitalised with COVID-19. The study presents important prospective data including detailed inpatient clinical profiles and structured follow-up extending to 12 months. The focus on individuals with severe and critical illness, many of whom required intensive care, fills an area of evidence that remains relatively under-represented.

There are nevertheless several substantive concerns.

First, the cohort is heavily skewed towards the most severe forms of COVID-19, with a high rate of intensive care utilisation and inpatient mortality. This limits the broader applicability of the findings and should be made more explicit early in the manuscript.

- *This is an important aspect of the study which we have now emphasised this in the abstract, weaknesses of the study, methods and results.*

Demographic missingness is concentrated in the most severe subgroup, particularly for ethnicity, and the current text does not sufficiently describe how this was handled or what biases may result.

- We shared a similar concern. As a consequence, we imported 'Ethnicity' as a three-level factor with levels 'white' (reference group), 'non-white' and 'unknown', and used this variable in our adjusted analyses, hence also controlling for the missing status for that variable. As stated above, more generally, we expect the amount of missingness per variable (often around 25 observations out of 425, refer to Supplementary Figure S1) to

be too small to have a large impact on estimates. This is now mentioned in the manuscript.

Second, the statistical analysis relies on a very large number of unadjusted hypothesis tests, raising the possibility of inflated false-positive findings.

- The main analyses (Tables 3.1-3.7, Tables 4.1 and 4.2) use logistic regression and time-to-event analysis both with and without adjusting for covariates. With regards to adjustment for multiple comparisons, the study is observational, and as such, we do not conclude causality from results, instead treating them as hypothesis-generating (which was the aim of this pragmatic study launched at the height of the COVID pandemic). The treatment effect estimates are often large, and greater in number than would be expected by chance. For example, Figure 2.3 reports the odds of nineteen COVID-19 symptoms by gender, seven of which show increased odds when using a 95% confidence interval.

Third, the clustering analysis of acute symptoms and comorbidities is exploratory in nature. The binary input data, limited separation of clusters and absence of validation do not match the current standard in LC phenotyping. As such, these findings should be repositioned as preliminary observations and not used to imply mechanistic distinctions.

We agree. The aim of the clustering was to help adjusting regression-like analyses for large differences in symptoms and comorbidities. This is now mentioned in the manuscript.

Regarding the reported sex differences in persistent symptoms, the finding that women experience higher burdens of breathlessness, fatigue and mood disturbance is consistent with the emerging LC literature. However, the manuscript does not adequately discuss the well-established biological and sociocultural factors underlying this pattern. There is a risk of oversimplification if female sex is presented without context or explanation. Expanding the discussion to recognise sex-specific immune and autonomic pathways, hormonal influences and social determinants of health would improve interpretability and avoid unintentional gender bias.

- *Thank you for raising this point. We have included this now in the discussion lines .*

Furthermore, thrombotic complications represent one of the most frequent and clinically relevant acute events in this cohort, yet receive limited attention in the discussion. Given the central role of associated coagulopathy in patient outcomes and recovery, deeper examination of this finding would be valuable.

- *We have amended the discussion to address this important issue.*

Similarly, the persistence of symptoms such as dizziness, breathlessness and gastrointestinal issues raises the possibility of post-COVID autonomic dysfunction, including postural orthostatic tachycardia syndrome. Acknowledging this recognised mechanism of long-term morbidity would strengthen the clinical relevance of the results and align the manuscript with current scientific understanding.

- *Thank you for raising this. We have included this now in the discussion.*

The interpretation of vaccination as a risk factor for mortality requires clearer acknowledgment of confounding by indication.

- *Yes, we have noted this in the methods: 'will have been prioritised based on their increased risk of severe COVID in accordance with national guidelines' line 185-6*
- *And in results: 'who had been pre-vaccinated in accordance with high risk of severe disease status'*
- *And in discussion: 'Vaccination at the time of the study, with the exception of the 2 patients in a vaccine trial, was selectively offered to patients at high risk of disease. As such, our data suggests that vaccine failure in high risk patients associated with shorter time to death in this cohort which may be understood in that this cohort had been pre-defined as vulnerable to death from COVID'*

The use of pandemic waves as proxies for variant exposure should also be presented with greater caution.

- *Yes, we have noted this in the methods: 'which approximated to the evolution of dominant SARS-CoV-2 variants'*
- I*n the discussion re raise the issues confounding this association: 'Whilst viral variants (and virulence) evolved through successive waves of the pandemic, medical preparedness and management also evolved and the seasons were distinct presenting multiple potential confounding variables contributing to this reported association.'*

Considerable attrition in long-term follow-up introduces the potential for survivorship and non-response bias, which must be addressed when interpreting recovery trajectories.

- *Thank you. This is important and we have given this more emphasis:*
  Stated weakness;
  Discussion: 'Whilst many patients at least one follow-up visit, a significant proportion of the cohort is not represented and as such there is a risk of ascertainment bias in the recovery analysis presented. However, it is unclear in which direction this bias may be noting some patients with more severe disabling symptoms may have felt unable to attend, particularly if they had a long distance to travel to the appointment. However, our results are consistent with what has been reported by other groups although for all such studies, those patients lost to follow up present an important cohort for whom recovery may be different.

Finally, improvements in clarity and presentation would enhance the manuscript's accessibility. The abstract should give greater emphasis to the study's limitations, and some tables may benefit from re-formatting to support readability.

- We agree on all counts: we have made extensive edits to improve the clarity of the manuscript text, made a number of changes to the tables and have expanded upon the study's limitations.

In conclusion, this study contributes meaningful data on outcomes after life-threatening COVID-19. With revisions to strengthen analytical transparency, update the interpretive framework and expand discussion of key mechanisms, the manuscript would provide a clearer and more clinically informative contribution to the field.
