# Supplementary material for: Defining the preadmission factors that modify risk of acute complications, survival and long-term recovery from COVID-19: prospective, longitudinal, multisite, cohort study based in England
Source: BMJ Open. 2026 Sep 21;16(9):e109653. doi: 10.1136/bmjopen-2025-109653 (PMC13599834; doi:10.1136/bmjopen-2025-109653)
Supplement: Supplementary file 4 [file bmjopen-16-9-s003.docx]

**Table S1.1: Characteristics of the study population by WHO COVID-19 severity**

| **Factors** | **Mild n=47 (11.1%)** | **Moderate pneumonia n=18 (4.2%)** | **Severe pneumonia (RR>30 or O2 requirement) n=107 (25.2%)** | **Critical ARDS (PaO2/FiO2<300) n=78 (18.4%)** | **Critical Sepsis + Critical Septic shock (vasopressor requirement) n=175 (41.2%)** | **Total n=425 (100.0%)** | **p-value** |
| --- | --- | --- | --- | --- | --- | --- | --- |
| **Age** |  |  |  |  |  |  | **0.0008** |
| <40 | 16 (34.0%) | 2 (11.1%) | 15 (14.0%) | 9 (11.5%) | 27 (15.4%) | 69 (16.2%) |  |
| 40-60 | 13 (27.7%) | 7 (38.9%) | 52 (48.6%) | 48 (61.5%) | 100 (57.1%) | 220 (51.8%) |  |
| 60-80 | 13 (27.7%) | 6 (33.3%) | 35 (32.7%) | 20 (25.6%) | 44 (25.1%) | 118 (27.8%) |  |
| >80 | 5 (10.6%) | 3 (16.7%) | 4 (3.7%) | 1 (1.3%) | 4 (2.3%) | 17 (4.0%) |  |
| Missing | 0 (0.0%) | 0 (0.0%) | 1 (0.9%) | 0 (0.0%) | 0 (0.0%) | 1 (0.2%) |  |
| **Gender** |  |  |  |  |  |  | **0.0266** |
| Male | 25 (53.2%) | 14 (77.8%) | 63 (58.9%) | 50 (64.1%) | 128 (73.1%) | 280 (65.9%) |  |
| Female | 22 (46.8%) | 4 (22.2%) | 44 (41.1%) | 28 (35.9%) | 47 (26.9%) | 145 (34.1%) |  |
| **Ethnicity** |  |  |  |  |  |  | **<0.0001** |
| ASIAN | 9 (19.1%) | 5 (27.8%) | 11 (10.3%) | 9 (11.5%) | 25 (14.3%) | 59 (13.9%) |  |
| BLACK | 0 (0.0%) | 1 (5.6%) | 5 (4.7%) | 1 (1.3%) | 3 (1.7%) | 10 (2.4%) |  |
| Not reported | 0 (0.0%) | 0 (0.0%) | 3 (2.8%) | 7 (9.0%) | 59 (33.7%) | 69 (16.2%) |  |
| Other | 0 (0.0%) | 1 (5.6%) | 2 (1.9%) | 2 (2.6%) | 3 (1.7%) | 8 (1.9%) |  |
| Other Mixed | 0 (0.0%) | 0 (0.0%) | 0 (0.0%) | 2 (2.6%) | 1 (0.6%) | 3 (0.7%) |  |
| WHITE | 38 (80.9%) | 11 (61.1%) | 86 (80.4%) | 57 (73.1%) | 84 (48.0%) | 276 (64.9%) |  |
| **Obesity** |  |  |  |  |  |  | **0.0010** |
| No | 41 (87.2%) | 16 (88.9%) | 84 (78.5%) | 50 (64.1%) | 112 (64.0%) | 303 (71.3%) |  |
| Yes | 6 (12.8%) | 2 (11.1%) | 23 (21.5%) | 28 (35.9%) | 63 (36.0%) | 122 (28.7%) |  |
| **Body Mass Index** |  |  |  |  |  |  | **0.0040** |
| <30 | 41 (87.2%) | 16 (88.9%) | 84 (78.5%) | 50 (64.1%) | 111 (63.4%) | 302 (71.1%) |  |
| 30 to 39 | 6 (12.8%) | 2 (11.1%) | 15 (14.0%) | 21 (26.9%) | 41 (23.4%) | 85 (20.0%) |  |
| >39 | 0 (0.0%) | 0 (0.0%) | 8 (7.5%) | 7 (9.0%) | 23 (13.1%) | 38 (8.9%) |  |
| **Number Of Comorbidity** |  |  |  |  |  |  | **0.6005** |
| 0 | 18 (38.3%) | 7 (38.9%) | 38 (35.5%) | 22 (28.2%) | 43 (24.6%) | 128 (30.1%) |  |
| 1 | 12 (25.5%) | 5 (27.8%) | 28 (26.2%) | 17 (21.8%) | 48 (27.4%) | 110 (25.9%) |  |
| 2 | 8 (17.0%) | 4 (22.2%) | 24 (22.4%) | 17 (21.8%) | 48 (27.4%) | 101 (23.8%) |  |
| 3 | 7 (14.9%) | 2 (11.1%) | 7 (6.5%) | 15 (19.2%) | 25 (14.3%) | 56 (13.2%) |  |
| 4 | 2 (4.3%) | 0 (0.0%) | 7 (6.5%) | 5 (6.4%) | 4 (2.3%) | 18 (4.2%) |  |
| 5 | 0 (0.0%) | 0 (0.0%) | 3 (2.8%) | 2 (2.6%) | 5 (2.9%) | 10 (2.4%) |  |
| 6 | 0 (0.0%) | 0 (0.0%) | 0 (0.0%) | 0 (0.0%) | 1 (0.6%) | 1 (0.2%) |  |
| 7 | 0 (0.0%) | 0 (0.0%) | 0 (0.0%) | 0 (0.0%) | 1 (0.6%) | 1 (0.2%) |  |
| **Chronic Respiratory Diseases** |  |  |  |  |  |  | **0.9217** |
| Yes | 7 (14.9%) | 2 (11.1%) | 15 (14.0%) | 13 (16.7%) | 22 (12.6%) | 59 (13.9%) |  |
| No | 40 (85.1%) | 16 (88.9%) | 92 (86.0%) | 65 (83.3%) | 153 (87.4%) | 366 (86.1%) |  |
| **Asthma** |  |  |  |  |  |  | **0.4035** |
| Yes | 5 (10.6%) | 1 (5.6%) | 20 (18.7%) | 16 (20.5%) | 26 (14.9%) | 68 (16.0%) |  |
| No | 42 (89.4%) | 17 (94.4%) | 87 (81.3%) | 62 (79.5%) | 149 (85.1%) | 357 (84.0%) |  |
| **Heart Disease** |  |  |  |  |  |  | **0.0243** |
| Yes | 8 (17.0%) | 2 (11.1%) | 16 (15.0%) | 8 (10.3%) | 9 (5.1%) | 43 (10.1%) |  |
| No | 39 (83.0%) | 16 (88.9%) | 91 (85.0%) | 70 (89.7%) | 166 (94.9%) | 382 (89.9%) |  |
| **Hypertension** |  |  |  |  |  |  | **0.0823** |
| Yes | 9 (19.1%) | 7 (38.9%) | 31 (29.0%) | 32 (41.0%) | 63 (36.0%) | 142 (33.4%) |  |
| No | 38 (80.9%) | 11 (61.1%) | 76 (71.0%) | 46 (59.0%) | 112 (64.0%) | 283 (66.6%) |  |
| **Renal Disease** |  |  |  |  |  |  | **0.5736** |
| Yes | 2 (4.3%) | 1 (5.6%) | 6 (5.6%) | 1 (1.3%) | 9 (5.1%) | 19 (4.5%) |  |
| No | 45 (95.7%) | 17 (94.4%) | 101 (94.4%) | 77 (98.7%) | 166 (94.9%) | 406 (95.5%) |  |
| **Liver Disease** |  |  |  |  |  |  | **0.4153** |
| Yes | 0 (0.0%) | 1 (5.6%) | 1 (0.9%) | 1 (1.3%) | 2 (1.1%) | 5 (1.2%) |  |
| No | 47 (100.0%) | 17 (94.4%) | 106 (99.1%) | 77 (98.7%) | 173 (98.9%) | 420 (98.8%) |  |
| **Neurological Disease** |  |  |  |  |  |  | **0.2517** |
| Yes | 3 (6.4%) | 1 (5.6%) | 1 (0.9%) | 3 (3.8%) | 5 (2.9%) | 13 (3.1%) |  |
| No | 44 (93.6%) | 17 (94.4%) | 106 (99.1%) | 75 (96.2%) | 170 (97.1%) | 412 (96.9%) |  |
| **Diabetes** |  |  |  |  |  |  | **0.6139** |
| Yes | 7 (14.9%) | 2 (11.1%) | 16 (15.0%) | 16 (20.5%) | 37 (21.1%) | 78 (18.4%) |  |
| No | 40 (85.1%) | 16 (88.9%) | 91 (85.0%) | 62 (79.5%) | 138 (78.9%) | 347 (81.6%) |  |
| **Immunosuppression Treatment** |  |  |  |  |  |  | **0.0011** |
| Yes | 4 (8.5%) | 0 (0.0%) | 6 (5.6%) | 5 (6.4%) | 0 (0.0%) | 15 (3.5%) |  |
| No | 43 (91.5%) | 18 (100.0%) | 101 (94.4%) | 73 (93.6%) | 175 (100.0%) | 410 (96.5%) |  |
| **Immunosuppression Disease** |  |  |  |  |  |  | **0.8088** |
| Yes | 1 (2.1%) | 0 (0.0%) | 2 (1.9%) | 2 (2.6%) | 2 (1.1%) | 7 (1.6%) |  |
| No | 46 (97.9%) | 18 (100.0%) | 105 (98.1%) | 76 (97.4%) | 173 (98.9%) | 418 (98.4%) |  |
| **Mental Illness** |  |  |  |  |  |  | **0.2553** |
| Yes | 0 (0.0%) | 1 (5.6%) | 4 (3.7%) | 5 (6.4%) | 13 (7.4%) | 23 (5.4%) |  |
| No | 47 (100.0%) | 17 (94.4%) | 103 (96.3%) | 73 (93.6%) | 162 (92.6%) | 402 (94.6%) |  |
| **Dementia** |  |  |  |  |  |  |  |
| Yes | 0 (0.0%) | 0 (0.0%) | 0 (0.0%) | 0 (0.0%) | 0 (0.0%) | 0 (0.0%) |  |
| No | 47 (100.0%) | 18 (100.0%) | 107 (100.0%) | 78 (100.0%) | 175 (100.0%) | 425 (100.0%) |  |
| **Pregnancy** |  |  |  |  |  |  | **1.0000** |
| Yes | 0 (0.0%) | 0 (0.0%) | 1 (0.9%) | 1 (1.3%) | 3 (1.7%) | 5 (1.2%) |  |
| No | 47 (100.0%) | 18 (100.0%) | 106 (99.1%) | 77 (98.7%) | 172 (98.3%) | 420 (98.8%) |  |
| **Admission Reason** |  |  |  |  |  |  | **<0.0001** |
| Acute cardiac event | 3 (6.4%) | 0 (0.0%) | 2 (1.9%) | 0 (0.0%) | 1 (0.6%) | 6 (1.4%) |  |
| COVID-19 | 8 (17.0%) | 15 (83.3%) | 96 (89.7%) | 70 (89.7%) | 169 (96.6%) | 358 (84.2%) |  |
| Flu | 0 (0.0%) | 0 (0.0%) | 0 (0.0%) | 1 (1.3%) | 0 (0.0%) | 1 (0.2%) |  |
| Other | 13 (27.7%) | 3 (16.7%) | 9 (8.4%) | 3 (3.8%) | 4 (2.3%) | 32 (7.5%) |  |
| Routine admission | 3 (6.4%) | 0 (0.0%) | 0 (0.0%) | 4 (5.1%) | 1 (0.6%) | 8 (1.9%) |  |
| Not admitted | 20 (42.6%) | 0 (0.0%) | 0 (0.0%) | 0 (0.0%) | 0 (0.0%) | 20 (4.7%) |  |
| **Duration of Symptoms** |  |  |  |  |  |  | **<0.0001** |
| No pre-admission symptoms | 6 (12.8%) | 1 (5.6%) | 1 (0.9%) | 6 (7.7%) | 3 (1.7%) | 17 (4.0%) |  |
| 0 to 7 days | 13 (27.7%) | 8 (44.4%) | 44 (41.1%) | 27 (34.6%) | 38 (21.7%) | 130 (30.6%) |  |
| 8 to 14 days | 5 (10.6%) | 8 (44.4%) | 47 (43.9%) | 32 (41.0%) | 48 (27.4%) | 140 (32.9%) |  |
| 15 to 28 days | 3 (6.4%) | 1 (5.6%) | 8 (7.5%) | 9 (11.5%) | 64 (36.6%) | 85 (20.0%) |  |
| Over 28 days | 0 (0.0%) | 0 (0.0%) | 7 (6.5%) | 4 (5.1%) | 22 (12.6%) | 33 (7.8%) |  |
| Not admitted | 20 (42.6%) | 0 (0.0%) | 0 (0.0%) | 0 (0.0%) | 0 (0.0%) | 20 (4.7%) |  |

**Table S1.2: Vaccination status by wave and WHO COVID-19 severity**

| **Wave** | **Vaccination** | **Mild n=47 (11.1%)** | **Moderate pneumonia n=18 (4.2%)** | **Severe pneumonia (RR>30 or O2 requirement) n=107 (25.2%)** | **Critical ARDS (PaO2/FiO2<300) n=78 (18.4%)** | **Critical Sepsis + Critical Septic shock (vasopressor requirement) n=175 (41.2%)** | **Total n=425 (100.0%)** | **P-value per wave** |
| --- | --- | --- | --- | --- | --- | --- | --- | --- |
| **1 wave** | No | 11 (100.0%) | 0 (-) | 11 (100.0%) | 22 (100.0%) | 46 (100.0%) | 90 (100.0%) | - |
|  | Yes | 0 (0.0%) | 0 (-) | 0 (0.0%) | 0 (0.0%) | 0 (0.0%) | 0 (0.0%) |  |
| **2 wave** | No | 10 (90.9%) | 11 (91.7%) | 39 (92.9%) | 39 (97.5%) | 104 (98.1%) | 203 (96.2%) | 0.1786 |
|  | Yes | 1 (9.1%) | 1 (8.3%) | 3 (7.1%) | 1 (2.5%) | 2 (1.9%) | 8 (3.8%) |  |
| **3 wave** | No | 5 (20.0%) | 4 (66.7%) | 28 (51.9%) | 6 (37.5%) | 15 (65.2%) | 58 (46.8%) | 0.0118 |
|  | Yes | 20 (80.0%) | 2 (33.3%) | 26 (48.1%) | 10 (62.5%) | 8 (34.8%) | 66 (53.2%) |  |
