# Supplementary material for: Defining the preadmission factors that modify risk of acute complications, survival and long-term recovery from COVID-19: prospective, longitudinal, multisite, cohort study based in England
Source: BMJ Open. 2026 Sep 21;16(9):e109653. doi: 10.1136/bmjopen-2025-109653 (PMC13599834; doi:10.1136/bmjopen-2025-109653)
Supplement: Supplementary file 5 [file bmjopen-16-9-s004.docx]

**Supplementary Tables S2: full model coefficients**

*Every fitted model (crude, conditional-on-severity and comprehensive) for each outcome, with all coefficients including the intercept. Indexed S2.k.m as referenced from the main tables.*

**S2.1 Thrombotic events**

**S2.1.1 (sev)	te ~ severity4**

| **Term** | **OR [95% CI]** | **P-val.** |
| --- | --- | --- |
| (Intercept) | 0.032 [0.004, 0.236] | **0.0007** |
| Severity: Severe | 1.278 [0.138, 11.868] | 0.8290 |
| Severity: Critical ARDS | 1.329 [0.133, 13.282] | 0.8089 |
| Severity: Critical Sepsis | 22.683 [3.012, 170.815] | **0.0024** |

**S2.1.2 (crude)	te ~ sex2**

| **Term** | **OR [95% CI]** | **P-val.** |
| --- | --- | --- |
| (Intercept) | 0.293 [0.215, 0.400] | **<0.0001** |
| Sex: Female | 0.547 [0.300, 0.997] | **0.0487** |

**S2.1.3 (cond)	te ~ severity4 + sex2**

| **Term** | **OR [95% CI]** | **P-val.** |
| --- | --- | --- |
| (Intercept) | 0.037 [0.005, 0.277] | **0.0013** |
| Severity: Severe | 1.283 [0.138, 11.931] | 0.8264 |
| Severity: Critical ARDS | 1.311 [0.131, 13.122] | 0.8179 |
| Severity: Critical Sepsis | 21.960 [2.912, 165.617] | **0.0027** |
| Sex: Female | 0.670 [0.341, 1.313] | 0.2433 |

**S2.1.4 (crude)	te ~ obesity2**

| **Term** | **OR [95% CI]** | **P-val.** |
| --- | --- | --- |
| (Intercept) | 0.250 [0.182, 0.343] | **<0.0001** |
| Obesity: Yes | 0.909 [0.509, 1.623] | 0.7472 |

**S2.1.5 (cond)	te ~ severity4 + obesity2**

| **Term** | **OR [95% CI]** | **P-val.** |
| --- | --- | --- |
| (Intercept) | 0.035 [0.005, 0.254] | **0.0009** |
| Severity: Severe | 1.299 [0.140, 12.073] | 0.8180 |
| Severity: Critical ARDS | 1.431 [0.143, 14.356] | 0.7604 |
| Severity: Critical Sepsis | 24.812 [3.275, 187.965] | **0.0019** |
| Obesity: Yes | 0.637 [0.331, 1.226] | 0.1772 |

**S2.1.6 (crude)	te ~ smoking3**

| **Term** | **OR [95% CI]** | **P-val.** |
| --- | --- | --- |
| (Intercept) | 0.265 [0.194, 0.361] | **<0.0001** |
| Smoking status: Ex-smoker | 0.813 [0.442, 1.497] | 0.5068 |
| Smoking status: Current | 0.315 [0.040, 2.477] | 0.2722 |

**S2.1.7 (cond)	te ~ severity4 + smoking3**

| **Term** | **OR [95% CI]** | **P-val.** |
| --- | --- | --- |
| (Intercept) | 0.033 [0.004, 0.244] | **0.0008** |
| Severity: Severe | 1.235 [0.133, 11.486] | 0.8530 |
| Severity: Critical ARDS | 1.258 [0.125, 12.609] | 0.8454 |
| Severity: Critical Sepsis | 22.150 [2.931, 167.359] | **0.0027** |
| Smoking status: Ex-smoker | 1.157 [0.570, 2.348] | 0.6858 |
| Smoking status: Current | 0.384 [0.042, 3.491] | 0.3951 |

**S2.1.8 (crude)	te ~ dursymp2**

| **Term** | **OR [95% CI]** | **P-val.** |
| --- | --- | --- |
| (Intercept) | 0.234 [0.156, 0.350] | **<0.0001** |
| Duration symptoms: More than 1 week | 1.069 [0.626, 1.826] | 0.8071 |

**S2.1.9 (cond)	te ~ severity4 + dursymp2**

| **Term** | **OR [95% CI]** | **P-val.** |
| --- | --- | --- |
| (Intercept) | 0.031 [0.004, 0.234] | **0.0008** |
| Severity: Severe | 1.272 [0.137, 11.814] | 0.8325 |
| Severity: Critical ARDS | 1.331 [0.133, 13.307] | 0.8078 |
| Severity: Critical Sepsis | 22.643 [3.007, 170.525] | **0.0025** |
| Duration symptoms: More than 1 week | 1.073 [0.584, 1.972] | 0.8211 |

**S2.1.10 (crude)	te ~ ethnicity3**

| **Term** | **OR [95% CI]** | **P-val.** |
| --- | --- | --- |
| (Intercept) | 0.179 [0.125, 0.256] | **<0.0001** |
| Ethnicity: Non-White | 1.141 [0.543, 2.394] | 0.7278 |
| Ethnicity: Unknown | 4.107 [2.128, 7.924] | **<0.0001** |

**S2.1.11 (cond)	te ~ severity4 + ethnicity3**

| **Term** | **OR [95% CI]** | **P-val.** |
| --- | --- | --- |
| (Intercept) | 0.032 [0.004, 0.240] | **0.0008** |
| Severity: Severe | 1.268 [0.136, 11.803] | 0.8349 |
| Severity: Critical ARDS | 1.280 [0.128, 12.839] | 0.8339 |
| Severity: Critical Sepsis | 20.037 [2.625, 152.925] | **0.0038** |
| Ethnicity: Non-White | 1.009 [0.437, 2.328] | 0.9839 |
| Ethnicity: Unknown | 1.488 [0.719, 3.079] | 0.2845 |

**S2.1.12 (crude)	te ~ vaccination2**

| **Term** | **OR [95% CI]** | **P-val.** |
| --- | --- | --- |
| (Intercept) | 0.271 [0.206, 0.357] | **<0.0001** |
| Vaccination: Yes | 0.335 [0.116, 0.968] | **0.0433** |

**S2.1.13 (cond)	te ~ severity4 + vaccination2**

| **Term** | **OR [95% CI]** | **P-val.** |
| --- | --- | --- |
| (Intercept) | 0.033 [0.004, 0.243] | **0.0008** |
| Severity: Severe | 1.314 [0.141, 12.268] | 0.8105 |
| Severity: Critical ARDS | 1.332 [0.133, 13.316] | 0.8074 |
| Severity: Critical Sepsis | 22.374 [2.967, 168.715] | **0.0026** |
| Vaccination: Yes | 0.814 [0.243, 2.722] | 0.7379 |

**S2.1.14 (crude)	te ~ wave3**

| **Term** | **OR [95% CI]** | **P-val.** |
| --- | --- | --- |
| (Intercept) | 0.431 [0.270, 0.689] | **0.0004** |
| Wave: 2 | 0.532 [0.291, 0.971] | **0.0399** |
| Wave: 3 | 0.297 [0.133, 0.667] | **0.0033** |

**S2.1.15 (cond)	te ~ severity4 + wave3**

| **Term** | **OR [95% CI]** | **P-val.** |
| --- | --- | --- |
| (Intercept) | 0.047 [0.006, 0.369] | **0.0036** |
| Severity: Severe | 1.208 [0.128, 11.364] | 0.8688 |
| Severity: Critical ARDS | 1.235 [0.123, 12.410] | 0.8580 |
| Severity: Critical Sepsis | 22.246 [2.928, 168.998] | **0.0027** |
| Wave: 2 | 0.558 [0.281, 1.107] | 0.0949 |
| Wave: 3 | 0.806 [0.303, 2.147] | 0.6664 |

**S2.1.16 (crude)	te ~ age3**

| **Term** | **OR [95% CI]** | **P-val.** |
| --- | --- | --- |
| (Intercept) | 0.179 [0.080, 0.401] | **<0.0001** |
| Age: 40-60 yrs | 1.767 [0.740, 4.220] | 0.1996 |
| Age: >60 yrs | 0.871 [0.330, 2.299] | 0.7796 |

**S2.1.17 (cond)	te ~ severity4 + age3**

| **Term** | **OR [95% CI]** | **P-val.** |
| --- | --- | --- |
| (Intercept) | 0.024 [0.003, 0.211] | **0.0008** |
| Severity: Severe | 1.180 [0.126, 11.013] | 0.8848 |
| Severity: Critical ARDS | 1.127 [0.112, 11.370] | 0.9195 |
| Severity: Critical Sepsis | 20.544 [2.712, 155.603] | **0.0034** |
| Age: 40-60 yrs | 1.936 [0.736, 5.089] | 0.1805 |
| Age: >60 yrs | 0.991 [0.340, 2.890] | 0.9866 |

**S2.1.18 (crude)	te ~ clusters2**

| **Term** | **OR [95% CI]** | **P-val.** |
| --- | --- | --- |
| (Intercept) | 0.070 [0.037, 0.134] | **<0.0001** |
| Clustering Symptoms: Cluster 2 | 5.968 [2.932, 12.149] | **<0.0001** |

**S2.1.19 (cond)	te ~ severity4 + clusters2**

| **Term** | **OR [95% CI]** | **P-val.** |
| --- | --- | --- |
| (Intercept) | 0.015 [0.002, 0.121] | **0.0001** |
| Severity: Severe | 1.720 [0.182, 16.241] | 0.6357 |
| Severity: Critical ARDS | 1.511 [0.150, 15.254] | 0.7264 |
| Severity: Critical Sepsis | 20.381 [2.686, 154.620] | **0.0035** |
| Clustering Symptoms: Cluster 2 | 2.983 [1.366, 6.514] | **0.0061** |

**S2.1.20 (crude)	te ~ clusterc2**

| **Term** | **OR [95% CI]** | **P-val.** |
| --- | --- | --- |
| (Intercept) | 0.212 [0.150, 0.301] | **<0.0001** |
| Clustering Comorbidities: Cluster 2 | 1.399 [0.818, 2.394] | 0.2205 |

**S2.1.21 (cond)	te ~ severity4 + clusterc2**

| **Term** | **OR [95% CI]** | **P-val.** |
| --- | --- | --- |
| (Intercept) | 0.026 [0.003, 0.197] | **0.0004** |
| Severity: Severe | 1.285 [0.138, 11.954] | 0.8255 |
| Severity: Critical ARDS | 1.370 [0.137, 13.733] | 0.7887 |
| Severity: Critical Sepsis | 23.514 [3.112, 177.679] | **0.0022** |
| Clustering Comorbidities: Cluster 2 | 1.582 [0.852, 2.938] | 0.1464 |

**S2.1.22 (comp) te ~ severity4+sex2+obesity2+smoking3+dursymp2+ethnicity3+vaccination2+ wave3 + age3+clusters2+clusterc2**

| **Term** | **OR [95% CI]** | **P-val.** |
| --- | --- | --- |
| (Intercept) | 0.009 [0.001, 0.153] | **0.0010** |
| Severity: Severe | 1.551 [0.156, 15.431] | 0.7082 |
| Severity: Critical ARDS | 1.366 [0.126, 14.793] | 0.7975 |
| Severity: Critical Sepsis | 21.426 [2.583, 177.737] | **0.0045** |
| Sex: Female | 0.870 [0.410, 1.847] | 0.7169 |
| Obesity: Yes | 0.707 [0.332, 1.510] | 0.3708 |
| Smoking status: Ex-smoker | 1.305 [0.605, 2.815] | 0.4976 |
| Smoking status: Current | 0.419 [0.041, 4.250] | 0.4620 |
| Duration symptoms: More than 1 week | 1.212 [0.624, 2.354] | 0.5703 |
| Ethnicity: Non-White | 1.030 [0.410, 2.590] | 0.9496 |
| Ethnicity: Unknown | 1.111 [0.491, 2.517] | 0.8006 |
| Vaccination: Yes | 0.652 [0.150, 2.836] | 0.5689 |
| Wave: 2 | 0.720 [0.341, 1.519] | 0.3887 |
| Wave: 3 | 1.194 [0.358, 3.982] | 0.7726 |
| Age: 40-60 yrs | 2.254 [0.771, 6.589] | 0.1375 |
| Age: >60 yrs | 1.281 [0.363, 4.518] | 0.7004 |
| Clustering Symptoms: Cluster 2 | 2.654 [1.159, 6.076] | **0.0209** |
| Clustering Comorbidities: Cluster 2 | 1.347 [0.665, 2.729] | 0.4077 |

**S2.2 Neurological events**

**S2.2.1 (sev)	neuroE ~ severity2**

| **Term** | **OR [95% CI]** | **P-val.** |
| --- | --- | --- |
| (Intercept) | 0.020 [0.007, 0.053] | **<0.0001** |
| Severity: Critical Sepsis | 13.527 [4.647, 39.375] | **<0.0001** |

**S2.2.2 (crude)	neuroE ~ sex2**

| **Term** | **OR [95% CI]** | **P-val.** |
| --- | --- | --- |
| (Intercept) | 0.119 [0.078, 0.182] | **<0.0001** |
| Sex: Female | 0.741 [0.342, 1.605] | 0.4475 |

**S2.2.3 (cond)	neuroE ~ severity2 + sex2**

| **Term** | **OR [95% CI]** | **P-val.** |
| --- | --- | --- |
| (Intercept) | 0.020 [0.007, 0.057] | **<0.0001** |
| Severity: Critical Sepsis | 13.463 [4.609, 39.324] | **<0.0001** |
| Sex: Female | 0.957 [0.422, 2.169] | 0.9159 |

**S2.2.4 (crude)	neuroE ~ obesity2**

| **Term** | **OR [95% CI]** | **P-val.** |
| --- | --- | --- |
| (Intercept) | 0.081 [0.050, 0.131] | **<0.0001** |
| Obesity: Yes | 2.145 [1.048, 4.389] | **0.0367** |

**S2.2.5 (cond)	neuroE ~ severity2 + obesity2**

| **Term** | **OR [95% CI]** | **P-val.** |
| --- | --- | --- |
| (Intercept) | 0.016 [0.006, 0.046] | **<0.0001** |
| Severity: Critical Sepsis | 12.946 [4.436, 37.779] | **<0.0001** |
| Obesity: Yes | 1.824 [0.855, 3.889] | 0.1198 |

**S2.2.6 (crude)	neuroE ~ smoking2**

| **Term** | **OR [95% CI]** | **P-val.** |
| --- | --- | --- |
| (Intercept) | 0.117 [0.077, 0.177] | **<0.0001** |
| Smoking status: Smoke(d) | 0.770 [0.347, 1.711] | 0.5218 |

**S2.2.7 (cond)	neuroE ~ severity2 + smoking2**

| **Term** | **OR [95% CI]** | **P-val.** |
| --- | --- | --- |
| (Intercept) | 0.020 [0.007, 0.055] | **<0.0001** |
| Severity: Critical Sepsis | 13.576 [4.643, 39.698] | **<0.0001** |
| Smoking status: Smoke(d) | 1.032 [0.442, 2.410] | 0.9416 |

**S2.2.8 (crude)	neuroE ~ dursymp2**

| **Term** | **OR [95% CI]** | **P-val.** |
| --- | --- | --- |
| (Intercept) | 0.125 [0.075, 0.207] | **<0.0001** |
| Duration symptoms: More than 1 week | 0.764 [0.376, 1.552] | 0.4565 |

**S2.2.9 (cond)	neuroE ~ severity2 + dursymp2**

| **Term** | **OR [95% CI]** | **P-val.** |
| --- | --- | --- |
| (Intercept) | 0.023 [0.008, 0.067] | **<0.0001** |
| Severity: Critical Sepsis | 13.612 [4.672, 39.658] | **<0.0001** |
| Duration symptoms: More than 1 week | 0.732 [0.346, 1.547] | 0.4137 |

**S2.2.10 (crude)	neuroE ~ ethnicity3**

| **Term** | **OR [95% CI]** | **P-val.** |
| --- | --- | --- |
| (Intercept) | 0.069 [0.041, 0.117] | **<0.0001** |
| Ethnicity: Non-White | 1.738 [0.677, 4.462] | 0.2506 |
| Ethnicity: Unknown | 4.320 [1.882, 9.914] | **0.0006** |

**S2.2.11 (cond)	neuroE ~ severity2 + ethnicity3**

| **Term** | **OR [95% CI]** | **P-val.** |
| --- | --- | --- |
| (Intercept) | 0.017 [0.006, 0.048] | **<0.0001** |
| Severity: Critical Sepsis | 11.682 [3.889, 35.092] | **<0.0001** |
| Ethnicity: Non-White | 1.623 [0.599, 4.397] | 0.3409 |
| Ethnicity: Unknown | 1.820 [0.757, 4.375] | 0.1808 |

**S2.2.12 (crude)	neuroE ~ vaccination2**

| **Term** | **OR [95% CI]** | **P-val.** |
| --- | --- | --- |
| (Intercept) | 0.124 [0.086, 0.177] | **<0.0001** |
| Vaccination: Yes | 0.172 [0.023, 1.289] | 0.0868 |

**S2.2.13 (cond)	neuroE ~ severity2 + vaccination2**

| **Term** | **OR [95% CI]** | **P-val.** |
| --- | --- | --- |
| (Intercept) | 0.023 [0.008, 0.062] | **<0.0001** |
| Severity: Critical Sepsis | 12.254 [4.174, 35.981] | **<0.0001** |
| Vaccination: Yes | 0.373 [0.047, 2.971] | 0.3514 |

**S2.2.14 (crude)	neuroE ~ wave3**

| **Term** | **OR [95% CI]** | **P-val.** |
| --- | --- | --- |
| (Intercept) | 0.153 [0.081, 0.288] | **<0.0001** |
| Wave: 2 | 0.650 [0.288, 1.472] | 0.3019 |
| Wave: 3 | 0.566 [0.208, 1.537] | 0.2638 |

**S2.2.15 (cond)	neuroE ~ severity2 + wave3**

| **Term** | **OR [95% CI]** | **P-val.** |
| --- | --- | --- |
| (Intercept) | 0.018 [0.005, 0.062] | **<0.0001** |
| Severity: Critical Sepsis | 16.561 [5.320, 51.554] | **<0.0001** |
| Wave: 2 | 0.737 [0.313, 1.736] | 0.4846 |
| Wave: 3 | 1.635 [0.526, 5.081] | 0.3951 |

**S2.2.16 (crude)	neuroE ~ age3**

| **Term** | **OR [95% CI]** | **P-val.** |
| --- | --- | --- |
| (Intercept) | 0.150 [0.064, 0.354] | **<0.0001** |
| Age: 40-60 yrs | 1.004 [0.386, 2.611] | 0.9934 |
| Age: >60 yrs | 0.185 [0.044, 0.776] | **0.0210** |

**S2.2.17 (cond)	neuroE ~ severity2 + age3**

| **Term** | **OR [95% CI]** | **P-val.** |
| --- | --- | --- |
| (Intercept) | 0.029 [0.008, 0.104] | **<0.0001** |
| Severity: Critical Sepsis | 13.297 [4.538, 38.967] | **<0.0001** |
| Age: 40-60 yrs | 0.953 [0.342, 2.655] | 0.9262 |
| Age: >60 yrs | 0.184 [0.042, 0.814] | **0.0257** |

**S2.2.18 (crude)	neuroE ~ clusters2**

| **Term** | **OR [95% CI]** | **P-val.** |
| --- | --- | --- |
| (Intercept) | 0.056 [0.027, 0.113] | **<0.0001** |
| Clustering Symptoms: Cluster 2 | 2.753 [1.209, 6.269] | **0.0159** |

**S2.2.19 (cond)	neuroE ~ severity2 + clusters2**

| **Term** | **OR [95% CI]** | **P-val.** |
| --- | --- | --- |
| (Intercept) | 0.018 [0.006, 0.053] | **<0.0001** |
| Severity: Critical Sepsis | 12.667 [4.165, 38.524] | **<0.0001** |
| Clustering Symptoms: Cluster 2 | 1.202 [0.490, 2.953] | 0.6876 |

**S2.2.20 (crude)	neuroE ~ clusterc2**

| **Term** | **OR [95% CI]** | **P-val.** |
| --- | --- | --- |
| (Intercept) | 0.119 [0.077, 0.183] | **<0.0001** |
| Clustering Comorbidities: Cluster 2 | 0.773 [0.364, 1.643] | 0.5035 |

**S2.2.21 (cond)	neuroE ~ severity2 + clusterc2**

| **Term** | **OR [95% CI]** | **P-val.** |
| --- | --- | --- |
| (Intercept) | 0.022 [0.008, 0.060] | **<0.0001** |
| Severity: Critical Sepsis | 13.550 [4.653, 39.459] | **<0.0001** |
| Clustering Comorbidities: Cluster 2 | 0.762 [0.345, 1.683] | 0.5014 |

**S2.2.22 (comp) neuroE ~ severity2+sex2+obesity2+smoking2+dursymp2+ethnicity3+vaccination2+ wave3 + age3+clusters2+clusterc2**

| **Term** | **OR [95% CI]** | **P-val.** |
| --- | --- | --- |
| (Intercept) | 0.022 [0.003, 0.183] | **0.0004** |
| Severity: Critical Sepsis | 15.977 [4.426, 57.668] | **<0.0001** |
| Sex: Female | 0.731 [0.286, 1.868] | 0.5129 |
| Obesity: Yes | 1.719 [0.708, 4.176] | 0.2315 |
| Smoking status: Smoke(d) | 1.358 [0.532, 3.463] | 0.5219 |
| Duration symptoms: More than 1 week | 0.804 [0.357, 1.809] | 0.5982 |
| Ethnicity: Non-White | 1.714 [0.579, 5.070] | 0.3305 |
| Ethnicity: Unknown | 1.812 [0.665, 4.936] | 0.2451 |
| Vaccination: Yes | 0.213 [0.023, 1.995] | 0.1757 |
| Wave: 2 | 0.762 [0.293, 1.979] | 0.5766 |
| Wave: 3 | 3.001 [0.774, 11.630] | 0.1118 |
| Age: 40-60 yrs | 1.151 [0.362, 3.661] | 0.8116 |
| Age: >60 yrs | 0.222 [0.041, 1.196] | 0.0799 |
| Clustering Symptoms: Cluster 2 | 0.815 [0.294, 2.262] | 0.6948 |
| Clustering Comorbidities: Cluster 2 | 0.495 [0.198, 1.240] | 0.1332 |

**S2.3 Renal complications**

**S2.3.1 (sev)	rcomp ~ severity2**

| **Term** | **OR [95% CI]** | **P-val.** |
| --- | --- | --- |
| (Intercept) | 0.040 [0.020, 0.082] | **<0.0001** |
| Severity: Critical Sepsis | 18.639 [8.536, 40.701] | **<0.0001** |

**S2.3.2 (crude)	rcomp ~ sex2**

| **Term** | **OR [95% CI]** | **P-val.** |
| --- | --- | --- |
| (Intercept) | 0.301 [0.220, 0.410] | **<0.0001** |
| Sex: Female | 0.534 [0.293, 0.971] | **0.0397** |

**S2.3.3 (cond)	rcomp ~ severity2 + sex2**

| **Term** | **OR [95% CI]** | **P-val.** |
| --- | --- | --- |
| (Intercept) | 0.047 [0.023, 0.098] | **<0.0001** |
| Severity: Critical Sepsis | 18.079 [8.267, 39.536] | **<0.0001** |
| Sex: Female | 0.648 [0.330, 1.271] | 0.2071 |

**S2.3.4 (crude)	rcomp ~ obesity2**

| **Term** | **OR [95% CI]** | **P-val.** |
| --- | --- | --- |
| (Intercept) | 0.206 [0.147, 0.288] | **<0.0001** |
| Obesity: Yes | 1.699 [0.984, 2.933] | 0.0572 |

**S2.3.5 (cond)	rcomp ~ severity2 + obesity2**

| **Term** | **OR [95% CI]** | **P-val.** |
| --- | --- | --- |
| (Intercept) | 0.036 [0.017, 0.076] | **<0.0001** |
| Severity: Critical Sepsis | 18.207 [8.328, 39.805] | **<0.0001** |
| Obesity: Yes | 1.431 [0.766, 2.673] | 0.2612 |

**S2.3.6 (crude)	rcomp ~ smoking3**

| **Term** | **OR [95% CI]** | **P-val.** |
| --- | --- | --- |
| (Intercept) | 0.285 [0.210, 0.387] | **<0.0001** |
| Smoking status: Ex-smoker | 0.599 [0.315, 1.141] | 0.1189 |
| Smoking status: Current | 0.638 [0.137, 2.968] | 0.5667 |

**S2.3.7 (cond)	rcomp ~ severity2 + smoking3**

| **Term** | **OR [95% CI]** | **P-val.** |
| --- | --- | --- |
| (Intercept) | 0.044 [0.021, 0.092] | **<0.0001** |
| Severity: Critical Sepsis | 18.250 [8.341, 39.932] | **<0.0001** |
| Smoking status: Ex-smoker | 0.777 [0.375, 1.612] | 0.4987 |
| Smoking status: Current | 0.892 [0.154, 5.170] | 0.8983 |

**S2.3.8 (crude)	rcomp ~ dursymp2**

| **Term** | **OR [95% CI]** | **P-val.** |
| --- | --- | --- |
| (Intercept) | 0.234 [0.156, 0.350] | **<0.0001** |
| Duration symptoms: More than 1 week | 1.103 [0.647, 1.881] | 0.7174 |

**S2.3.9 (cond)	rcomp ~ severity2 + dursymp2**

| **Term** | **OR [95% CI]** | **P-val.** |
| --- | --- | --- |
| (Intercept) | 0.038 [0.017, 0.084] | **<0.0001** |
| Severity: Critical Sepsis | 18.651 [8.540, 40.733] | **<0.0001** |
| Duration symptoms: More than 1 week | 1.118 [0.609, 2.054] | 0.7180 |

**S2.3.10 (crude)	rcomp ~ ethnicity3**

| **Term** | **OR [95% CI]** | **P-val.** |
| --- | --- | --- |
| (Intercept) | 0.191 [0.134, 0.271] | **<0.0001** |
| Ethnicity: Non-White | 0.633 [0.268, 1.495] | 0.2967 |
| Ethnicity: Unknown | 4.855 [2.540, 9.279] | **<0.0001** |

**S2.3.11 (cond)	rcomp ~ severity2 + ethnicity3**

| **Term** | **OR [95% CI]** | **P-val.** |
| --- | --- | --- |
| (Intercept) | 0.044 [0.021, 0.090] | **<0.0001** |
| Severity: Critical Sepsis | 16.022 [7.138, 35.962] | **<0.0001** |
| Ethnicity: Non-White | 0.492 [0.193, 1.254] | 0.1372 |
| Ethnicity: Unknown | 1.785 [0.865, 3.685] | 0.1170 |

**S2.3.12 (crude)	rcomp ~ vaccination2**

| **Term** | **OR [95% CI]** | **P-val.** |
| --- | --- | --- |
| (Intercept) | 0.266 [0.201, 0.351] | **<0.0001** |
| Vaccination: Yes | 0.537 [0.219, 1.321] | 0.1760 |

**S2.3.13 (cond)	rcomp ~ severity2 + vaccination2**

| **Term** | **OR [95% CI]** | **P-val.** |
| --- | --- | --- |
| (Intercept) | 0.036 [0.017, 0.078] | **<0.0001** |
| Severity: Critical Sepsis | 20.305 [8.946, 46.089] | **<0.0001** |
| Vaccination: Yes | 1.587 [0.529, 4.761] | 0.4103 |

**S2.3.14 (crude)	rcomp ~ wave3**

| **Term** | **OR [95% CI]** | **P-val.** |
| --- | --- | --- |
| (Intercept) | 0.566 [0.362, 0.886] | **0.0127** |
| Wave: 2 | 0.332 [0.182, 0.607] | **0.0003** |
| Wave: 3 | 0.252 [0.116, 0.547] | **0.0005** |

**S2.3.15 (cond)	rcomp ~ severity2 + wave3**

| **Term** | **OR [95% CI]** | **P-val.** |
| --- | --- | --- |
| (Intercept) | 0.073 [0.031, 0.175] | **<0.0001** |
| Severity: Critical Sepsis | 21.268 [9.213, 49.095] | **<0.0001** |
| Wave: 2 | 0.298 [0.148, 0.600] | **0.0007** |
| Wave: 3 | 0.663 [0.257, 1.710] | 0.3949 |

**S2.3.16 (crude)	rcomp ~ age3**

| **Term** | **OR [95% CI]** | **P-val.** |
| --- | --- | --- |
| (Intercept) | 0.211 [0.098, 0.451] | **0.0001** |
| Age: 40-60 yrs | 1.141 [0.491, 2.651] | 0.7586 |
| Age: >60 yrs | 1.310 [0.540, 3.179] | 0.5500 |

**S2.3.17 (cond)	rcomp ~ severity2 + age3**

| **Term** | **OR [95% CI]** | **P-val.** |
| --- | --- | --- |
| (Intercept) | 0.031 [0.010, 0.092] | **<0.0001** |
| Severity: Critical Sepsis | 19.560 [8.887, 43.048] | **<0.0001** |
| Age: 40-60 yrs | 1.107 [0.430, 2.851] | 0.8326 |
| Age: >60 yrs | 1.713 [0.623, 4.712] | 0.2972 |

**S2.3.18 (crude)	rcomp ~ clusters2**

| **Term** | **OR [95% CI]** | **P-val.** |
| --- | --- | --- |
| (Intercept) | 0.070 [0.037, 0.134] | **<0.0001** |
| Clustering Symptoms: Cluster 2 | 6.115 [3.006, 12.441] | **<0.0001** |

**S2.3.19 (cond)	rcomp ~ severity2 + clusters2**

| **Term** | **OR [95% CI]** | **P-val.** |
| --- | --- | --- |
| (Intercept) | 0.022 [0.009, 0.054] | **<0.0001** |
| Severity: Critical Sepsis | 13.913 [6.259, 30.930] | **<0.0001** |
| Clustering Symptoms: Cluster 2 | 3.007 [1.384, 6.537] | **0.0054** |

**S2.3.20 (crude)	rcomp ~ clusterc2**

| **Term** | **OR [95% CI]** | **P-val.** |
| --- | --- | --- |
| (Intercept) | 0.284 [0.206, 0.391] | **<0.0001** |
| Clustering Comorbidities: Cluster 2 | 0.672 [0.382, 1.184] | 0.1691 |

**S2.3.21 (cond)	rcomp ~ severity2 + clusterc2**

| **Term** | **OR [95% CI]** | **P-val.** |
| --- | --- | --- |
| (Intercept) | 0.047 [0.023, 0.099] | **<0.0001** |
| Severity: Critical Sepsis | 18.981 [8.666, 41.576] | **<0.0001** |
| Clustering Comorbidities: Cluster 2 | 0.610 [0.322, 1.156] | 0.1299 |

**S2.3.22 (comp) rcomp ~ severity2+sex2+obesity2+smoking3+dursymp2+ethnicity3+vaccination2+ wave3 + age3+clusters2+clusterc2**

| **Term** | **OR [95% CI]** | **P-val.** |
| --- | --- | --- |
| (Intercept) | 0.015 [0.002, 0.117] | **0.0001** |
| Severity: Critical Sepsis | 19.659 [7.565, 51.083] | **<0.0001** |
| Sex: Female | 0.619 [0.287, 1.337] | 0.2222 |
| Obesity: Yes | 2.465 [1.118, 5.436] | **0.0253** |
| Smoking status: Ex-smoker | 0.699 [0.305, 1.603] | 0.3976 |
| Smoking status: Current | 0.928 [0.140, 6.170] | 0.9385 |
| Duration symptoms: More than 1 week | 1.407 [0.691, 2.864] | 0.3467 |
| Ethnicity: Non-White | 0.755 [0.264, 2.164] | 0.6015 |
| Ethnicity: Unknown | 2.020 [0.867, 4.706] | 0.1031 |
| Vaccination: Yes | 1.761 [0.439, 7.073] | 0.4247 |
| Wave: 2 | 0.298 [0.134, 0.662] | **0.0030** |
| Wave: 3 | 0.984 [0.277, 3.495] | 0.9804 |
| Age: 40-60 yrs | 1.082 [0.347, 3.370] | 0.8923 |
| Age: >60 yrs | 2.640 [0.707, 9.862] | 0.1487 |
| Clustering Symptoms: Cluster 2 | 3.320 [1.371, 8.041] | **0.0078** |
| Clustering Comorbidities: Cluster 2 | 0.585 [0.264, 1.297] | 0.1873 |

**S2.4 Cardiac events**

**S2.4.1 (sev)	carE ~ severity4**

| **Term** | **OR [95% CI]** | **P-val.** |
| --- | --- | --- |
| (Intercept) | 0.032 [0.004, 0.236] | **0.0007** |
| Severity: Severe | 0.949 [0.095, 9.455] | 0.9644 |
| Severity: Critical ARDS | 1.329 [0.133, 13.282] | 0.8089 |
| Severity: Critical Sepsis | 6.948 [0.907, 53.235] | 0.0621 |

**S2.4.2 (crude)	carE ~ sex2**

| **Term** | **OR [95% CI]** | **P-val.** |
| --- | --- | --- |
| (Intercept) | 0.114 [0.074, 0.175] | **<0.0001** |
| Sex: Female | 0.777 [0.357, 1.691] | 0.5251 |

**S2.4.3 (cond)	carE ~ severity4 + sex2**

| **Term** | **OR [95% CI]** | **P-val.** |
| --- | --- | --- |
| (Intercept) | 0.033 [0.004, 0.247] | **0.0009** |
| Severity: Severe | 0.949 [0.095, 9.459] | 0.9647 |
| Severity: Critical ARDS | 1.326 [0.133, 13.263] | 0.8100 |
| Severity: Critical Sepsis | 6.912 [0.900, 53.066] | 0.0630 |
| Sex: Female | 0.955 [0.426, 2.141] | 0.9119 |

**S2.4.4 (crude)	carE ~ obesity2**

| **Term** | **OR [95% CI]** | **P-val.** |
| --- | --- | --- |
| (Intercept) | 0.091 [0.058, 0.144] | **<0.0001** |
| Obesity: Yes | 1.505 [0.719, 3.150] | 0.2778 |

**S2.4.5 (cond)	carE ~ severity4 + obesity2**

| **Term** | **OR [95% CI]** | **P-val.** |
| --- | --- | --- |
| (Intercept) | 0.031 [0.004, 0.227] | **0.0006** |
| Severity: Severe | 0.940 [0.094, 9.366] | 0.9576 |
| Severity: Critical ARDS | 1.271 [0.126, 12.772] | 0.8388 |
| Severity: Critical Sepsis | 6.664 [0.865, 51.309] | 0.0686 |
| Obesity: Yes | 1.264 [0.587, 2.721] | 0.5497 |

**S2.4.6 (crude)	carE ~ smoking3**

| **Term** | **OR [95% CI]** | **P-val.** |
| --- | --- | --- |
| (Intercept) | 0.122 [0.081, 0.183] | **<0.0001** |
| Smoking status: Ex-smoker | 0.546 [0.217, 1.372] | 0.1982 |
| Smoking status: Current | 0.683 [0.085, 5.466] | 0.7191 |

**S2.4.7 (cond)	carE ~ severity4 + smoking3**

| **Term** | **OR [95% CI]** | **P-val.** |
| --- | --- | --- |
| (Intercept) | 0.036 [0.005, 0.271] | **0.0012** |
| Severity: Severe | 0.944 [0.094, 9.444] | 0.9610 |
| Severity: Critical ARDS | 1.363 [0.136, 13.712] | 0.7925 |
| Severity: Critical Sepsis | 6.752 [0.876, 52.059] | 0.0669 |
| Smoking status: Ex-smoker | 0.660 [0.255, 1.706] | 0.3911 |
| Smoking status: Current | 0.881 [0.102, 7.593] | 0.9079 |

**S2.4.8 (crude)	carE ~ dursymp2**

| **Term** | **OR [95% CI]** | **P-val.** |
| --- | --- | --- |
| (Intercept) | 0.133 [0.082, 0.218] | **<0.0001** |
| Duration symptoms: More than 1 week | 0.625 [0.304, 1.285] | 0.2011 |

**S2.4.9 (cond)	carE ~ severity4 + dursymp2**

| **Term** | **OR [95% CI]** | **P-val.** |
| --- | --- | --- |
| (Intercept) | 0.041 [0.005, 0.308] | **0.0019** |
| Severity: Severe | 0.985 [0.099, 9.842] | 0.9897 |
| Severity: Critical ARDS | 1.313 [0.131, 13.160] | 0.8168 |
| Severity: Critical Sepsis | 7.133 [0.928, 54.810] | 0.0590 |
| Duration symptoms: More than 1 week | 0.603 [0.286, 1.271] | 0.1836 |

**S2.4.10 (crude)	carE ~ ethnicity3**

| **Term** | **OR [95% CI]** | **P-val.** |
| --- | --- | --- |
| (Intercept) | 0.085 [0.052, 0.137] | **<0.0001** |
| Ethnicity: Non-White | 1.203 [0.457, 3.168] | 0.7077 |
| Ethnicity: Unknown | 2.477 [1.043, 5.880] | **0.0398** |

**S2.4.11 (cond)	carE ~ severity4 + ethnicity3**

| **Term** | **OR [95% CI]** | **P-val.** |
| --- | --- | --- |
| (Intercept) | 0.031 [0.004, 0.235] | **0.0008** |
| Severity: Severe | 0.959 [0.096, 9.596] | 0.9717 |
| Severity: Critical ARDS | 1.329 [0.132, 13.351] | 0.8089 |
| Severity: Critical Sepsis | 6.715 [0.858, 52.582] | 0.0697 |
| Ethnicity: Non-White | 1.106 [0.406, 3.015] | 0.8437 |
| Ethnicity: Unknown | 1.168 [0.468, 2.914] | 0.7395 |

**S2.4.12 (crude)	carE ~ vaccination2**

| **Term** | **OR [95% CI]** | **P-val.** |
| --- | --- | --- |
| (Intercept) | 0.107 [0.073, 0.157] | **<0.0001** |
| Vaccination: Yes | 0.850 [0.285, 2.534] | 0.7699 |

**S2.4.13 (cond)	carE ~ severity4 + vaccination2**

| **Term** | **OR [95% CI]** | **P-val.** |
| --- | --- | --- |
| (Intercept) | 0.029 [0.004, 0.219] | **0.0006** |
| Severity: Severe | 0.860 [0.085, 8.686] | 0.8983 |
| Severity: Critical ARDS | 1.318 [0.132, 13.206] | 0.8144 |
| Severity: Critical Sepsis | 7.377 [0.955, 56.966] | 0.0554 |
| Vaccination: Yes | 1.806 [0.541, 6.029] | 0.3366 |

**S2.4.14 (crude)	carE ~ wave3**

| **Term** | **OR [95% CI]** | **P-val.** |
| --- | --- | --- |
| (Intercept) | 0.153 [0.081, 0.288] | **<0.0001** |
| Wave: 2 | 0.695 [0.310, 1.560] | 0.3782 |
| Wave: 3 | 0.394 [0.131, 1.188] | 0.0983 |

**S2.4.15 (cond)	carE ~ severity4 + wave3**

| **Term** | **OR [95% CI]** | **P-val.** |
| --- | --- | --- |
| (Intercept) | 0.039 [0.005, 0.319] | **0.0025** |
| Severity: Severe | 0.948 [0.094, 9.595] | 0.9642 |
| Severity: Critical ARDS | 1.282 [0.128, 12.873] | 0.8326 |
| Severity: Critical Sepsis | 6.728 [0.871, 51.985] | 0.0677 |
| Wave: 2 | 0.780 [0.339, 1.797] | 0.5599 |
| Wave: 3 | 0.824 [0.249, 2.734] | 0.7523 |

**S2.4.16 (crude)	carE ~ age3**

| **Term** | **OR [95% CI]** | **P-val.** |
| --- | --- | --- |
| (Intercept) | 0.095 [0.034, 0.266] | **<0.0001** |
| Age: 40-60 yrs | 1.228 [0.399, 3.784] | 0.7205 |
| Age: >60 yrs | 0.926 [0.270, 3.174] | 0.9032 |

**S2.4.17 (cond)	carE ~ severity4 + age3**

| **Term** | **OR [95% CI]** | **P-val.** |
| --- | --- | --- |
| (Intercept) | 0.030 [0.003, 0.278] | **0.0020** |
| Severity: Severe | 0.932 [0.093, 9.316] | 0.9524 |
| Severity: Critical ARDS | 1.279 [0.127, 12.918] | 0.8347 |
| Severity: Critical Sepsis | 6.742 [0.873, 52.056] | 0.0673 |
| Age: 40-60 yrs | 1.190 [0.373, 3.795] | 0.7693 |
| Age: >60 yrs | 1.025 [0.288, 3.652] | 0.9694 |

**S2.4.18 (crude)	carE ~ clusters2**

| **Term** | **OR [95% CI]** | **P-val.** |
| --- | --- | --- |
| (Intercept) | 0.041 [0.018, 0.093] | **<0.0001** |
| Clustering Symptoms: Cluster 2 | 3.888 [1.562, 9.676] | **0.0035** |

**S2.4.19 (cond)	carE ~ severity4 + clusters2**

| **Term** | **OR [95% CI]** | **P-val.** |
| --- | --- | --- |
| (Intercept) | 0.019 [0.002, 0.157] | **0.0002** |
| Severity: Severe | 1.182 [0.116, 12.002] | 0.8874 |
| Severity: Critical ARDS | 1.465 [0.145, 14.755] | 0.7460 |
| Severity: Critical Sepsis | 6.189 [0.802, 47.744] | 0.0803 |
| Clustering Symptoms: Cluster 2 | 2.225 [0.840, 5.896] | 0.1076 |

**S2.4.20 (crude)	carE ~ clusterc2**

| **Term** | **OR [95% CI]** | **P-val.** |
| --- | --- | --- |
| (Intercept) | 0.085 [0.052, 0.139] | **<0.0001** |
| Clustering Comorbidities: Cluster 2 | 1.637 [0.797, 3.363] | 0.1799 |

**S2.4.21 (cond)	carE ~ severity4 + clusterc2**

| **Term** | **OR [95% CI]** | **P-val.** |
| --- | --- | --- |
| (Intercept) | 0.025 [0.003, 0.191] | **0.0004** |
| Severity: Severe | 0.954 [0.096, 9.533] | 0.9682 |
| Severity: Critical ARDS | 1.379 [0.137, 13.830] | 0.7849 |
| Severity: Critical Sepsis | 7.160 [0.931, 55.048] | 0.0586 |
| Clustering Comorbidities: Cluster 2 | 1.721 [0.816, 3.632] | 0.1542 |

**S2.4.22 (comp) carE ~ severity4+sex2+obesity2+smoking3+dursymp2+ethnicity3+vaccination2+ wave3 + age3+clusters2+clusterc2**

| **Term** | **OR [95% CI]** | **P-val.** |
| --- | --- | --- |
| (Intercept) | 0.014 [0.001, 0.247] | **0.0036** |
| Severity: Severe | 1.130 [0.105, 12.207] | 0.9196 |
| Severity: Critical ARDS | 1.405 [0.132, 14.914] | 0.7780 |
| Severity: Critical Sepsis | 6.477 [0.781, 53.715] | 0.0834 |
| Sex: Female | 0.992 [0.417, 2.358] | 0.9856 |
| Obesity: Yes | 1.468 [0.622, 3.465] | 0.3806 |
| Smoking status: Ex-smoker | 0.652 [0.241, 1.769] | 0.4013 |
| Smoking status: Current | 1.062 [0.114, 9.915] | 0.9577 |
| Duration symptoms: More than 1 week | 0.657 [0.298, 1.449] | 0.2983 |
| Ethnicity: Non-White | 1.065 [0.345, 3.283] | 0.9133 |
| Ethnicity: Unknown | 1.036 [0.376, 2.857] | 0.9449 |
| Vaccination: Yes | 2.944 [0.630, 13.769] | 0.1701 |
| Wave: 2 | 0.854 [0.342, 2.132] | 0.7347 |
| Wave: 3 | 0.562 [0.122, 2.598] | 0.4611 |
| Age: 40-60 yrs | 1.335 [0.372, 4.793] | 0.6575 |
| Age: >60 yrs | 1.585 [0.362, 6.938] | 0.5407 |
| Clustering Symptoms: Cluster 2 | 2.006 [0.719, 5.601] | 0.1837 |
| Clustering Comorbidities: Cluster 2 | 1.969 [0.851, 4.558] | 0.1136 |

**S2.5 Pneumothorax**

**S2.5.1 (sev)	pneumotx ~ severity2**

| **Term** | **OR [95% CI]** | **P-val.** |
| --- | --- | --- |
| (Intercept) | 0.025 [0.010, 0.060] | **<0.0001** |
| Severity: Critical Sepsis | 5.835 [2.113, 16.115] | **0.0007** |

**S2.5.2 (crude)	pneumotx ~ sex2**

| **Term** | **OR [95% CI]** | **P-val.** |
| --- | --- | --- |
| (Intercept) | 0.071 [0.042, 0.121] | **<0.0001** |
| Sex: Female | 0.974 [0.401, 2.366] | 0.9535 |

**S2.5.3 (cond)	pneumotx ~ severity2 + sex2**

| **Term** | **OR [95% CI]** | **P-val.** |
| --- | --- | --- |
| (Intercept) | 0.023 [0.009, 0.061] | **<0.0001** |
| Severity: Critical Sepsis | 5.959 [2.144, 16.561] | **0.0006** |
| Sex: Female | 1.198 [0.480, 2.987] | 0.6987 |

**S2.5.4 (crude)	pneumotx ~ obesity2**

| **Term** | **OR [95% CI]** | **P-val.** |
| --- | --- | --- |
| (Intercept) | 0.067 [0.040, 0.112] | **<0.0001** |
| Obesity: Yes | 1.200 [0.493, 2.922] | 0.6880 |

**S2.5.5 (cond)	pneumotx ~ severity2 + obesity2**

| **Term** | **OR [95% CI]** | **P-val.** |
| --- | --- | --- |
| (Intercept) | 0.025 [0.010, 0.062] | **<0.0001** |
| Severity: Critical Sepsis | 5.830 [2.103, 16.157] | **0.0007** |
| Obesity: Yes | 1.011 [0.406, 2.514] | 0.9817 |

**S2.5.6 (crude)	pneumotx ~ smoking2**

| **Term** | **OR [95% CI]** | **P-val.** |
| --- | --- | --- |
| (Intercept) | 0.081 [0.050, 0.132] | **<0.0001** |
| Smoking status: Smoke(d) | 0.590 [0.213, 1.633] | 0.3100 |

**S2.5.7 (cond)	pneumotx ~ severity2 + smoking2**

| **Term** | **OR [95% CI]** | **P-val.** |
| --- | --- | --- |
| (Intercept) | 0.028 [0.011, 0.071] | **<0.0001** |
| Severity: Critical Sepsis | 5.649 [2.036, 15.671] | **0.0009** |
| Smoking status: Smoke(d) | 0.723 [0.255, 2.047] | 0.5412 |

**S2.5.8 (crude)	pneumotx ~ dursymp2**

| **Term** | **OR [95% CI]** | **P-val.** |
| --- | --- | --- |
| (Intercept) | 0.117 [0.070, 0.196] | **<0.0001** |
| Duration symptoms: More than 1 week | 0.319 [0.128, 0.796] | **0.0143** |

**S2.5.9 (cond)	pneumotx ~ severity2 + dursymp2**

| **Term** | **OR [95% CI]** | **P-val.** |
| --- | --- | --- |
| (Intercept) | 0.041 [0.016, 0.104] | **<0.0001** |
| Severity: Critical Sepsis | 6.081 [2.182, 16.945] | **0.0006** |
| Duration symptoms: More than 1 week | 0.301 [0.118, 0.765] | **0.0117** |

**S2.5.10 (crude)	pneumotx ~ ethnicity3**

| **Term** | **OR [95% CI]** | **P-val.** |
| --- | --- | --- |
| (Intercept) | 0.055 [0.031, 0.098] | **<0.0001** |
| Ethnicity: Non-White | 0.883 [0.242, 3.228] | 0.8508 |
| Ethnicity: Unknown | 3.318 [1.282, 8.592] | **0.0135** |

**S2.5.11 (cond)	pneumotx ~ severity2 + ethnicity3**

| **Term** | **OR [95% CI]** | **P-val.** |
| --- | --- | --- |
| (Intercept) | 0.025 [0.010, 0.063] | **<0.0001** |
| Severity: Critical Sepsis | 4.917 [1.684, 14.354] | **0.0036** |
| Ethnicity: Non-White | 0.809 [0.217, 3.014] | 0.7517 |
| Ethnicity: Unknown | 1.707 [0.620, 4.699] | 0.3009 |

**S2.5.12 (crude)	pneumotx ~ vaccination2**

| **Term** | **OR [95% CI]** | **P-val.** |
| --- | --- | --- |
| (Intercept) | 0.079 [0.051, 0.122] | **<0.0001** |
| Vaccination: Yes | 0.269 [0.035, 2.042] | 0.2042 |

**S2.5.13 (cond)	pneumotx ~ severity2 + vaccination2**

| **Term** | **OR [95% CI]** | **P-val.** |
| --- | --- | --- |
| (Intercept) | 0.028 [0.011, 0.070] | **<0.0001** |
| Severity: Critical Sepsis | 5.368 [1.916, 15.040] | **0.0014** |
| Vaccination: Yes | 0.476 [0.060, 3.787] | 0.4829 |

**S2.5.14 (crude)	pneumotx ~ wave3**

| **Term** | **OR [95% CI]** | **P-val.** |
| --- | --- | --- |
| (Intercept) | 0.078 [0.034, 0.179] | **<0.0001** |
| Wave: 2 | 0.850 [0.303, 2.384] | 0.7580 |
| Wave: 3 | 0.939 [0.290, 3.036] | 0.9163 |

**S2.5.15 (cond)	pneumotx ~ severity2 + wave3**

| **Term** | **OR [95% CI]** | **P-val.** |
| --- | --- | --- |
| (Intercept) | 0.018 [0.005, 0.065] | **<0.0001** |
| Severity: Critical Sepsis | 7.389 [2.478, 22.030] | **0.0003** |
| Wave: 2 | 0.962 [0.336, 2.754] | 0.9420 |
| Wave: 3 | 2.109 [0.588, 7.559] | 0.2519 |

**S2.5.16 (crude)	pneumotx ~ age3**

| **Term** | **OR [95% CI]** | **P-val.** |
| --- | --- | --- |
| (Intercept) | 0.095 [0.034, 0.266] | **<0.0001** |
| Age: 40-60 yrs | 1.092 [0.351, 3.398] | 0.8786 |
| Age: >60 yrs | 0.095 [0.010, 0.879] | **0.0381** |

**S2.5.17 (cond)	pneumotx ~ severity2 + age3**

| **Term** | **OR [95% CI]** | **P-val.** |
| --- | --- | --- |
| (Intercept) | 0.035 [0.009, 0.128] | **<0.0001** |
| Severity: Critical Sepsis | 5.569 [1.998, 15.517] | **0.0010** |
| Age: 40-60 yrs | 1.059 [0.330, 3.393] | 0.9237 |
| Age: >60 yrs | 0.099 [0.011, 0.929] | **0.0429** |

**S2.5.18 (crude)	pneumotx ~ clusters2**

| **Term** | **OR [95% CI]** | **P-val.** |
| --- | --- | --- |
| (Intercept) | 0.020 [0.006, 0.063] | **<0.0001** |
| Clustering Symptoms: Cluster 2 | 5.644 [1.645, 19.365] | **0.0059** |

**S2.5.19 (cond)	pneumotx ~ severity2 + clusters2**

| **Term** | **OR [95% CI]** | **P-val.** |
| --- | --- | --- |
| (Intercept) | 0.013 [0.004, 0.044] | **<0.0001** |
| Severity: Critical Sepsis | 4.063 [1.412, 11.693] | **0.0094** |
| Clustering Symptoms: Cluster 2 | 3.428 [0.948, 12.398] | 0.0603 |

**S2.5.20 (crude)	pneumotx ~ clusterc2**

| **Term** | **OR [95% CI]** | **P-val.** |
| --- | --- | --- |
| (Intercept) | 0.059 [0.033, 0.105] | **<0.0001** |
| Clustering Comorbidities: Cluster 2 | 1.566 [0.670, 3.659] | 0.3002 |

**S2.5.21 (cond)	pneumotx ~ severity2 + clusterc2**

| **Term** | **OR [95% CI]** | **P-val.** |
| --- | --- | --- |
| (Intercept) | 0.020 [0.008, 0.054] | **<0.0001** |
| Severity: Critical Sepsis | 5.886 [2.128, 16.281] | **0.0006** |
| Clustering Comorbidities: Cluster 2 | 1.613 [0.677, 3.845] | 0.2802 |

**S2.5.22 (comp) pneumotx ~ severity2+sex2+obesity2+smoking2+dursymp2+ethnicity3+vaccination2+ wave3 + age3+clusters2+clusterc2**

| **Term** | **OR [95% CI]** | **P-val.** |
| --- | --- | --- |
| (Intercept) | 0.018 [0.002, 0.180] | **0.0006** |
| Severity: Critical Sepsis | 4.248 [1.224, 14.751] | **0.0227** |
| Sex: Female | 0.946 [0.331, 2.703] | 0.9167 |
| Obesity: Yes | 0.773 [0.267, 2.241] | 0.6353 |
| Smoking status: Smoke(d) | 0.824 [0.268, 2.535] | 0.7360 |
| Duration symptoms: More than 1 week | 0.322 [0.120, 0.869] | **0.0252** |
| Ethnicity: Non-White | 0.534 [0.130, 2.192] | 0.3836 |
| Ethnicity: Unknown | 1.008 [0.313, 3.254] | 0.9888 |
| Vaccination: Yes | 0.307 [0.031, 3.014] | 0.3110 |
| Wave: 2 | 1.294 [0.400, 4.186] | 0.6670 |
| Wave: 3 | 3.638 [0.790, 16.762] | 0.0976 |
| Age: 40-60 yrs | 1.512 [0.384, 5.955] | 0.5540 |
| Age: >60 yrs | 0.162 [0.014, 1.810] | 0.1394 |
| Clustering Symptoms: Cluster 2 | 3.258 [0.794, 13.370] | 0.1012 |
| Clustering Comorbidities: Cluster 2 | 1.155 [0.425, 3.141] | 0.7775 |

**S2.6 Survival to discharge**

**S2.6.1 (sev)	I(survival2=='Alive') ~ severity3**

| **Term** | **OR [95% CI]** | **P-val.** |
| --- | --- | --- |
| (Intercept) | 25.800 [10.560, 63.037] | **<0.0001** |
| Severity: Critical ARDS | 0.446 [0.131, 1.513] | 0.1951 |
| Severity: Critical Sepsis | 0.051 [0.020, 0.131] | **<0.0001** |

**S2.6.2 (crude)	I(survival2=='Alive') ~ sex2**

| **Term** | **OR [95% CI]** | **P-val.** |
| --- | --- | --- |
| (Intercept) | 3.490 [2.557, 4.765] | **<0.0001** |
| Sex: Female | 1.315 [0.754, 2.294] | 0.3342 |

**S2.6.3 (cond)	I(survival2=='Alive') ~ severity3 + sex2**

| **Term** | **OR [95% CI]** | **P-val.** |
| --- | --- | --- |
| (Intercept) | 25.813 [10.182, 65.441] | **<0.0001** |
| Severity: Critical ARDS | 0.446 [0.131, 1.514] | 0.1952 |
| Severity: Critical Sepsis | 0.051 [0.019, 0.132] | **<0.0001** |
| Sex: Female | 0.999 [0.531, 1.879] | 0.9970 |

**S2.6.4 (crude)	I(survival2=='Alive') ~ obesity2**

| **Term** | **OR [95% CI]** | **P-val.** |
| --- | --- | --- |
| (Intercept) | 3.939 [2.879, 5.389] | **<0.0001** |
| Obesity: Yes | 0.910 [0.525, 1.578] | 0.7363 |

**S2.6.5 (cond)	I(survival2=='Alive') ~ severity3 + obesity2**

| **Term** | **OR [95% CI]** | **P-val.** |
| --- | --- | --- |
| (Intercept) | 24.663 [10.021, 60.697] | **<0.0001** |
| Severity: Critical ARDS | 0.431 [0.126, 1.468] | 0.1781 |
| Severity: Critical Sepsis | 0.049 [0.019, 0.127] | **<0.0001** |
| Obesity: Yes | 1.254 [0.675, 2.328] | 0.4737 |

**S2.6.6 (crude)	I(survival2=='Alive') ~ smoking2**

| **Term** | **OR [95% CI]** | **P-val.** |
| --- | --- | --- |
| (Intercept) | 3.321 [2.464, 4.478] | **<0.0001** |
| Smoking status: Smoke(d) | 1.647 [0.907, 2.992] | 0.1014 |

**S2.6.7 (cond)	I(survival2=='Alive') ~ severity3 + smoking2**

| **Term** | **OR [95% CI]** | **P-val.** |
| --- | --- | --- |
| (Intercept) | 23.808 [9.537, 59.429] | **<0.0001** |
| Severity: Critical ARDS | 0.441 [0.130, 1.498] | 0.1893 |
| Severity: Critical Sepsis | 0.052 [0.020, 0.134] | **<0.0001** |
| Smoking status: Smoke(d) | 1.288 [0.659, 2.517] | 0.4589 |

**S2.6.8 (crude)	I(survival2=='Alive') ~ dursymp2**

| **Term** | **OR [95% CI]** | **P-val.** |
| --- | --- | --- |
| (Intercept) | 3.968 [2.676, 5.883] | **<0.0001** |
| Duration symptoms: More than 1 week | 0.936 [0.556, 1.576] | 0.8038 |

**S2.6.9 (cond)	I(survival2=='Alive') ~ severity3 + dursymp2**

| **Term** | **OR [95% CI]** | **P-val.** |
| --- | --- | --- |
| (Intercept) | 27.682 [10.543, 72.682] | **<0.0001** |
| Severity: Critical ARDS | 0.441 [0.130, 1.499] | 0.1898 |
| Severity: Critical Sepsis | 0.050 [0.019, 0.131] | **<0.0001** |
| Duration symptoms: More than 1 week | 0.891 [0.495, 1.603] | 0.7000 |

**S2.6.10 (crude)	I(survival2=='Alive') ~ ethnicity3**

| **Term** | **OR [95% CI]** | **P-val.** |
| --- | --- | --- |
| (Intercept) | 6.091 [4.215, 8.802] | **<0.0001** |
| Ethnicity: Non-White | 1.384 [0.582, 3.289] | 0.4621 |
| Ethnicity: Unknown | 0.095 [0.048, 0.185] | **<0.0001** |

**S2.6.11 (cond)	I(survival2=='Alive') ~ severity3 + ethnicity3**

| **Term** | **OR [95% CI]** | **P-val.** |
| --- | --- | --- |
| (Intercept) | 24.976 [10.074, 61.924] | **<0.0001** |
| Severity: Critical ARDS | 0.538 [0.155, 1.862] | 0.3276 |
| Severity: Critical Sepsis | 0.079 [0.030, 0.211] | **<0.0001** |
| Ethnicity: Non-White | 1.620 [0.645, 4.070] | 0.3050 |
| Ethnicity: Unknown | 0.210 [0.100, 0.438] | **<0.0001** |

**S2.6.12 (crude)	I(survival2=='Alive') ~ vaccination2**

| **Term** | **OR [95% CI]** | **P-val.** |
| --- | --- | --- |
| (Intercept) | 3.576 [2.722, 4.698] | **<0.0001** |
| Vaccination: Yes | 1.718 [0.739, 3.996] | 0.2090 |

**S2.6.13 (cond)	I(survival2=='Alive') ~ severity3 + vaccination2**

| **Term** | **OR [95% CI]** | **P-val.** |
| --- | --- | --- |
| (Intercept) | 30.233 [11.565, 79.034] | **<0.0001** |
| Severity: Critical ARDS | 0.419 [0.122, 1.437] | 0.1667 |
| Severity: Critical Sepsis | 0.044 [0.016, 0.121] | **<0.0001** |
| Vaccination: Yes | 0.577 [0.206, 1.614] | 0.2949 |

**S2.6.14 (crude)	I(survival2=='Alive') ~ wave3**

| **Term** | **OR [95% CI]** | **P-val.** |
| --- | --- | --- |
| (Intercept) | 2.773 [1.703, 4.514] | **<0.0001** |
| Wave: 2 | 1.329 [0.726, 2.433] | 0.3572 |
| Wave: 3 | 2.164 [1.009, 4.641] | **0.0474** |

**S2.6.15 (cond)	I(survival2=='Alive') ~ severity3 + wave3**

| **Term** | **OR [95% CI]** | **P-val.** |
| --- | --- | --- |
| (Intercept) | 30.855 [10.089, 94.365] | **<0.0001** |
| Severity: Critical ARDS | 0.393 [0.113, 1.368] | 0.1422 |
| Severity: Critical Sepsis | 0.041 [0.015, 0.115] | **<0.0001** |
| Wave: 2 | 1.140 [0.581, 2.237] | 0.7028 |
| Wave: 3 | 0.639 [0.250, 1.634] | 0.3500 |

**S2.6.16 (crude)	I(survival2=='Alive') ~ age3**

| **Term** | **OR [95% CI]** | **P-val.** |
| --- | --- | --- |
| (Intercept) | 8.200 [3.241, 20.750] | **<0.0001** |
| Age: 40-60 yrs | 0.449 [0.167, 1.209] | 0.1132 |
| Age: >60 yrs | 0.393 [0.141, 1.094] | 0.0738 |

**S2.6.17 (cond)	I(survival2=='Alive') ~ severity3 + age3**

| **Term** | **OR [95% CI]** | **P-val.** |
| --- | --- | --- |
| (Intercept) | 71.813 [18.851, 273.567] | **<0.0001** |
| Severity: Critical ARDS | 0.414 [0.121, 1.420] | 0.1609 |
| Severity: Critical Sepsis | 0.045 [0.017, 0.120] | **<0.0001** |
| Age: 40-60 yrs | 0.412 [0.142, 1.192] | 0.1017 |
| Age: >60 yrs | 0.262 [0.086, 0.802] | **0.0190** |

**S2.6.18 (crude)	I(survival2=='Alive') ~ clusters2**

| **Term** | **OR [95% CI]** | **P-val.** |
| --- | --- | --- |
| (Intercept) | 7.611 [4.656, 12.441] | **<0.0001** |
| Clustering Symptoms: Cluster 2 | 0.339 [0.190, 0.607] | **0.0003** |

**S2.6.19 (cond)	I(survival2=='Alive') ~ severity3 + clusters2**

| **Term** | **OR [95% CI]** | **P-val.** |
| --- | --- | --- |
| (Intercept) | 28.733 [11.195, 73.743] | **<0.0001** |
| Severity: Critical ARDS | 0.453 [0.133, 1.540] | 0.2048 |
| Severity: Critical Sepsis | 0.056 [0.021, 0.148] | **<0.0001** |
| Clustering Symptoms: Cluster 2 | 0.772 [0.394, 1.513] | 0.4513 |

**S2.6.20 (crude)	I(survival2=='Alive') ~ clusterc2**

| **Term** | **OR [95% CI]** | **P-val.** |
| --- | --- | --- |
| (Intercept) | 3.018 [2.225, 4.094] | **<0.0001** |
| Clustering Comorbidities: Cluster 2 | 2.080 [1.161, 3.728] | **0.0139** |

**S2.6.21 (cond)	I(survival2=='Alive') ~ severity3 + clusterc2**

| **Term** | **OR [95% CI]** | **P-val.** |
| --- | --- | --- |
| (Intercept) | 19.631 [7.925, 48.627] | **<0.0001** |
| Severity: Critical ARDS | 0.465 [0.136, 1.586] | 0.2214 |
| Severity: Critical Sepsis | 0.048 [0.019, 0.126] | **<0.0001** |
| Clustering Comorbidities: Cluster 2 | 2.463 [1.286, 4.719] | **0.0066** |

**S2.6.22 (comp) I(survival2=='Alive') ~ severity3+sex2+obesity2+smoking2+dursymp2+ethnicity3+vaccination2+ wave3 + age3+clusters2+clusterc2**

| **Term** | **OR [95% CI]** | **P-val.** |
| --- | --- | --- |
| (Intercept) | 184.181 [20.646, 1643.024] | **<0.0001** |
| Severity: Critical ARDS | 0.334 [0.088, 1.266] | 0.1068 |
| Severity: Critical Sepsis | 0.040 [0.012, 0.134] | **<0.0001** |
| Sex: Female | 1.220 [0.568, 2.617] | 0.6104 |
| Obesity: Yes | 0.958 [0.454, 2.018] | 0.9095 |
| Smoking status: Smoke(d) | 1.264 [0.587, 2.722] | 0.5493 |
| Duration symptoms: More than 1 week | 0.786 [0.393, 1.570] | 0.4948 |
| Ethnicity: Non-White | 1.283 [0.467, 3.525] | 0.6290 |
| Ethnicity: Unknown | 0.152 [0.066, 0.353] | **<0.0001** |
| Vaccination: Yes | 0.853 [0.225, 3.244] | 0.8160 |
| Wave: 2 | 0.887 [0.396, 1.985] | 0.7699 |
| Wave: 3 | 0.265 [0.068, 1.027] | 0.0546 |
| Age: 40-60 yrs | 0.361 [0.102, 1.280] | 0.1146 |
| Age: >60 yrs | 0.205 [0.050, 0.834] | **0.0269** |
| Clustering Symptoms: Cluster 2 | 0.774 [0.355, 1.690] | 0.5205 |
| Clustering Comorbidities: Cluster 2 | 2.679 [1.206, 5.948] | **0.0155** |

**S2.7 Time to death (Cox)**

**S2.7.1 (sev) Surv(time.in, time.out, survival2=='Death', type='counting') ~ severity3**

| **Term** | **HR [95% CI]** | **P-val.** |
| --- | --- | --- |
| Severity: Critical ARDS | 1.524 [0.378, 6.144] | 0.5536 |
| Severity: Critical Sepsis | 2.694 [0.808, 8.985] | 0.1068 |

**S2.7.2 (crude) Surv(time.in, time.out, survival2=='Death', type='counting') ~ sex2**

| **Term** | **HR [95% CI]** | **P-val.** |
| --- | --- | --- |
| Sex: Female | 0.690 [0.243, 1.962] | 0.4866 |

**S2.7.3 (cond) Surv(time.in, time.out, survival2=='Death', type='counting') ~ severity3 + sex2**

| **Term** | **HR [95% CI]** | **P-val.** |
| --- | --- | --- |
| Severity: Critical ARDS | 1.516 [0.376, 6.119] | 0.5587 |
| Severity: Critical Sepsis | 2.695 [0.806, 9.009] | 0.1074 |
| Sex: Female | 0.691 [0.243, 1.963] | 0.4873 |

**S2.7.4 (crude) Surv(time.in, time.out, survival2=='Death', type='counting') ~ obesity2**

| **Term** | **HR [95% CI]** | **P-val.** |
| --- | --- | --- |
| Obesity: Yes | 1.162 [0.425, 3.175] | 0.7701 |

**S2.7.5 (cond) Surv(time.in, time.out, survival2=='Death', type='counting') ~ severity3 + obesity2**

| **Term** | **HR [95% CI]** | **P-val.** |
| --- | --- | --- |
| Severity: Critical ARDS | 1.516 [0.375, 6.125] | 0.5592 |
| Severity: Critical Sepsis | 2.674 [0.798, 8.959] | 0.1108 |
| Obesity: Yes | 1.075 [0.393, 2.943] | 0.8873 |

**S2.7.6 (crude) Surv(time.in, time.out, survival2=='Death', type='counting') ~ smoking2**

| **Term** | **HR [95% CI]** | **P-val.** |
| --- | --- | --- |
| Smoking status: Smoke(d) | 1.417 [0.536, 3.746] | 0.4821 |

**S2.7.7 (cond) Surv(time.in, time.out, survival2=='Death', type='counting') ~ severity3 + smoking2**

| **Term** | **HR [95% CI]** | **P-val.** |
| --- | --- | --- |
| Severity: Critical ARDS | 1.669 [0.412, 6.761] | 0.4733 |
| Severity: Critical Sepsis | 3.480 [1.000, 12.115] | 0.0500 |
| Smoking status: Smoke(d) | 1.891 [0.679, 5.266] | 0.2229 |

**S2.7.8 (crude) Surv(time.in, time.out, survival2=='Death', type='counting') ~ dursymp2**

| **Term** | **HR [95% CI]** | **P-val.** |
| --- | --- | --- |
| Duration symptoms: More than 1 week | 0.838 [0.310, 2.263] | 0.7274 |

**S2.7.9 (cond) Surv(time.in, time.out, survival2=='Death', type='counting') ~ severity3 + dursymp2**

| **Term** | **HR [95% CI]** | **P-val.** |
| --- | --- | --- |
| Severity: Critical ARDS | 1.394 [0.334, 5.821] | 0.6485 |
| Severity: Critical Sepsis | 2.722 [0.814, 9.096] | 0.1039 |
| Duration symptoms: More than 1 week | 0.755 [0.267, 2.139] | 0.5972 |

**S2.7.10 (crude) Surv(time.in, time.out, survival2=='Death', type='counting') ~ ethnicity2**

| **Term** | **HR [95% CI]** | **P-val.** |
| --- | --- | --- |
| Ethnicity: Non-White | 0.475 [0.136, 1.662] | 0.2439 |

**S2.7.11 (cond) Surv(time.in, time.out, survival2=='Death', type='counting') ~ severity3 + ethnicity2**

| **Term** | **HR [95% CI]** | **P-val.** |
| --- | --- | --- |
| Severity: Critical ARDS | 1.600 [0.396, 6.463] | 0.5092 |
| Severity: Critical Sepsis | 3.080 [0.913, 10.392] | 0.0698 |
| Ethnicity: Non-White | 0.403 [0.114, 1.428] | 0.1591 |

**S2.7.12 (crude) Surv(time.in, time.out, survival2=='Death', type='counting') ~ vaccination2**

| **Term** | **HR [95% CI]** | **P-val.** |
| --- | --- | --- |
| Vaccination: Yes | 1.281 [0.450, 3.644] | 0.6425 |

**S2.7.13 (cond) Surv(time.in, time.out, survival2=='Death', type='counting') ~ severity3 + vaccination2**

| **Term** | **HR [95% CI]** | **P-val.** |
| --- | --- | --- |
| Severity: Critical ARDS | 1.634 [0.399, 6.683] | 0.4946 |
| Severity: Critical Sepsis | 2.875 [0.853, 9.694] | 0.0885 |
| Vaccination: Yes | 1.488 [0.514, 4.304] | 0.4633 |

**S2.7.14 (crude) Surv(time.in, time.out, survival2=='Death', type='counting') ~ clusters2**

| **Term** | **HR [95% CI]** | **P-val.** |
| --- | --- | --- |
| Clustering Symptoms: Cluster 2 | 0.330 [0.074, 1.466] | 0.1451 |

**S2.7.15 (cond) Surv(time.in, time.out, survival2=='Death', type='counting') ~ severity3 + clusters2**

| **Term** | **HR [95% CI]** | **P-val.** |
| --- | --- | --- |
| Severity: Critical ARDS | 1.704 [0.378, 7.676] | 0.4874 |
| Severity: Critical Sepsis | 3.302 [0.885, 12.328] | 0.0755 |
| Clustering Symptoms: Cluster 2 | 0.313 [0.070, 1.400] | 0.1284 |

**S2.7.16 (crude) Surv(time.in, time.out, survival2=='Death', type='counting') ~ clusterc2**

| **Term** | **HR [95% CI]** | **P-val.** |
| --- | --- | --- |
| Clustering Comorbidities: Cluster 2 | 0.158 [0.021, 1.195] | 0.0739 |

**S2.7.17 (cond) Surv(time.in, time.out, survival2=='Death', type='counting') ~ severity3 + clusterc2**

| **Term** | **HR [95% CI]** | **P-val.** |
| --- | --- | --- |
| Severity: Critical ARDS | 1.654 [0.406, 6.736] | 0.4827 |
| Severity: Critical Sepsis | 3.431 [1.026, 11.474] | **0.0453** |
| Clustering Comorbidities: Cluster 2 | 0.128 [0.017, 0.972] | **0.0469** |

**S2.7.18 (comp) Surv(time.in, time.out, survival2=='Death', type='counting') ~ severity3+sex2+obesity2+smoking2+dursymp2+ethnicity2+vaccination2 + clusters2+clusterc2**

| **Term** | **HR [95% CI]** | **P-val.** |
| --- | --- | --- |
| Severity: Critical ARDS | 3.263 [0.472, 22.546] | 0.2305 |
| Severity: Critical Sepsis | 30.920 [3.128, 305.606] | **0.0033** |
| Sex: Female | 0.384 [0.116, 1.272] | 0.1175 |
| Obesity: Yes | 2.013 [0.634, 6.394] | 0.2353 |
| Smoking status: Smoke(d) | 2.151 [0.581, 7.962] | 0.2515 |
| Duration symptoms: More than 1 week | 0.180 [0.041, 0.789] | **0.0230** |
| Ethnicity: Non-White | 0.378 [0.089, 1.603] | 0.1869 |
| Vaccination: Yes | 6.360 [1.226, 33.007] | **0.0277** |
| Clustering Symptoms: Cluster 2 | 0.243 [0.044, 1.339] | 0.1043 |
| Clustering Comorbidities: Cluster 2 | 0.030 [0.003, 0.318] | **0.0035** |

**S2.8 MRC breathlessness**

**S2.8.1 (sev)	mrc2 ~ time.factor + severity3 + (1|patient)**

| **Term** | **OR [95% CI]** | **P-val.** |
| --- | --- | --- |
| (Intercept) | 1.275 [0.337, 4.822] | 0.7204 |
| Time: 6 months | 0.699 [0.283, 1.725] | 0.4366 |
| Time: 12 months | 0.873 [0.344, 2.213] | 0.7745 |
| Severity: Critical ARDS | 1.626 [0.186, 14.176] | 0.6602 |
| Severity: Critical Sepsis | 31.786 [2.704, 373.638] | **0.0059** |

**S2.8.2 (crude)	mrc2 ~ time.factor + sex2 + (1|patient)**

| **Term** | **OR [95% CI]** | **P-val.** |
| --- | --- | --- |
| (Intercept) | 1.183 [0.370, 3.778] | 0.7770 |
| Time: 6 months | 0.722 [0.292, 1.785] | 0.4802 |
| Time: 12 months | 1.041 [0.410, 2.643] | 0.9330 |
| Sex: Female | 27.530 [2.727, 277.951] | **0.0049** |

**S2.8.3 (cond)	mrc2 ~ time.factor + severity3 + sex2 + (1|patient)**

| **Term** | **OR [95% CI]** | **P-val.** |
| --- | --- | --- |
| (Intercept) | 0.234 [0.036, 1.532] | 0.1298 |
| Time: 6 months | 0.680 [0.263, 1.758] | 0.4261 |
| Time: 12 months | 0.872 [0.332, 2.291] | 0.7813 |
| Severity: Critical ARDS | 2.713 [0.272, 27.042] | 0.3948 |
| Severity: Critical Sepsis | 73.828 [2.973, 1833.578] | **0.0087** |
| Sex: Female | 51.944 [3.076, 877.188] | **0.0062** |

**S2.8.4 (crude)	mrc2 ~ time.factor + obesity2 + (1|patient)**

| **Term** | **OR [95% CI]** | **P-val.** |
| --- | --- | --- |
| (Intercept) | 1.856 [0.571, 6.030] | 0.3036 |
| Time: 6 months | 0.727 [0.297, 1.783] | 0.4863 |
| Time: 12 months | 0.989 [0.394, 2.482] | 0.9814 |
| Obesity: Yes | 9.695 [1.100, 85.413] | **0.0407** |

**S2.8.5 (cond)	mrc2 ~ time.factor + severity3 + obesity2 + (1|patient)**

| **Term** | **OR [95% CI]** | **P-val.** |
| --- | --- | --- |
| (Intercept) | 0.848 [0.203, 3.535] | 0.8209 |
| Time: 6 months | 0.700 [0.281, 1.746] | 0.4447 |
| Time: 12 months | 0.862 [0.337, 2.205] | 0.7565 |
| Severity: Critical ARDS | 1.382 [0.152, 12.540] | 0.7739 |
| Severity: Critical Sepsis | 22.721 [2.110, 244.696] | **0.0100** |
| Obesity: Yes | 5.648 [0.835, 38.212] | 0.0759 |

**S2.8.6 (crude)	mrc2 ~ time.factor + smoking2 + (1|patient)**

| **Term** | **OR [95% CI]** | **P-val.** |
| --- | --- | --- |
| (Intercept) | 3.995 [1.202, 13.273] | **0.0238** |
| Time: 6 months | 0.728 [0.307, 1.727] | 0.4718 |
| Time: 12 months | 1.029 [0.423, 2.501] | 0.9498 |
| Smoking status: Smoke(d) | 0.535 [0.105, 2.716] | 0.4506 |

**S2.8.7 (cond)	mrc2 ~ time.factor + severity3 + smoking2 + (1|patient)**

| **Term** | **OR [95% CI]** | **P-val.** |
| --- | --- | --- |
| (Intercept) | 1.519 [0.356, 6.484] | 0.5726 |
| Time: 6 months | 0.697 [0.281, 1.726] | 0.4351 |
| Time: 12 months | 0.872 [0.344, 2.216] | 0.7743 |
| Severity: Critical ARDS | 1.739 [0.191, 15.806] | 0.6232 |
| Severity: Critical Sepsis | 32.396 [2.689, 390.294] | **0.0062** |
| Smoking status: Smoke(d) | 0.563 [0.095, 3.335] | 0.5271 |

**S2.8.8 (crude)	mrc2 ~ time.factor + dursymp2 + (1|patient)**

| **Term** | **OR [95% CI]** | **P-val.** |
| --- | --- | --- |
| (Intercept) | 1.963 [0.534, 7.211] | 0.3099 |
| Time: 6 months | 0.742 [0.313, 1.759] | 0.4986 |
| Time: 12 months | 1.037 [0.427, 2.515] | 0.9367 |
| Duration symptoms: More than 1 week | 2.266 [0.492, 10.444] | 0.2939 |

**S2.8.9 (cond)	mrc2 ~ time.factor + severity3 + dursymp2 + (1|patient)**

| **Term** | **OR [95% CI]** | **P-val.** |
| --- | --- | --- |
| (Intercept) | 0.838 [0.158, 4.434] | 0.8350 |
| Time: 6 months | 0.709 [0.287, 1.754] | 0.4569 |
| Time: 12 months | 0.880 [0.347, 2.233] | 0.7886 |
| Severity: Critical ARDS | 1.680 [0.193, 14.617] | 0.6384 |
| Severity: Critical Sepsis | 30.390 [2.576, 358.537] | **0.0067** |
| Duration symptoms: More than 1 week | 2.001 [0.375, 10.670] | 0.4165 |

**S2.8.10 (crude)	mrc2 ~ time.factor + ethnicity2 + (1|patient)**

| **Term** | **OR [95% CI]** | **P-val.** |
| --- | --- | --- |
| (Intercept) | 2.678 [0.926, 7.743] | 0.0690 |
| Time: 6 months | 0.727 [0.306, 1.725] | 0.4696 |
| Time: 12 months | 1.025 [0.421, 2.495] | 0.9562 |
| Ethnicity: Non-White | 2.400 [0.403, 14.288] | 0.3361 |

**S2.8.11 (cond)	mrc2 ~ time.factor + severity3 + ethnicity2 + (1|patient)**

| **Term** | **OR [95% CI]** | **P-val.** |
| --- | --- | --- |
| (Intercept) | 1.215 [0.310, 4.767] | 0.7798 |
| Time: 6 months | 0.699 [0.283, 1.725] | 0.4373 |
| Time: 12 months | 0.874 [0.345, 2.215] | 0.7766 |
| Severity: Critical ARDS | 1.607 [0.185, 13.988] | 0.6672 |
| Severity: Critical Sepsis | 29.921 [2.517, 355.751] | **0.0071** |
| Ethnicity: Non-White | 1.324 [0.201, 8.712] | 0.7701 |

**S2.8.12 (crude)	mrc2 ~ time.factor + vaccination2 + (1|patient)**

| **Term** | **OR [95% CI]** | **P-val.** |
| --- | --- | --- |
| (Intercept) | 3.429 [1.169, 10.055] | **0.0248** |
| Time: 6 months | 0.729 [0.308, 1.727] | 0.4727 |
| Time: 12 months | 1.018 [0.418, 2.479] | 0.9678 |
| Vaccination: Yes | 0.660 [0.075, 5.812] | 0.7081 |

**S2.8.13 (cond)	mrc2 ~ time.factor + severity3 + vaccination2 + (1|patient)**

| **Term** | **OR [95% CI]** | **P-val.** |
| --- | --- | --- |
| (Intercept) | 1.207 [0.287, 5.069] | 0.7975 |
| Time: 6 months | 0.699 [0.283, 1.726] | 0.4370 |
| Time: 12 months | 0.878 [0.346, 2.233] | 0.7854 |
| Severity: Critical ARDS | 1.687 [0.186, 15.288] | 0.6421 |
| Severity: Critical Sepsis | 33.174 [2.701, 407.451] | **0.0062** |
| Vaccination: Yes | 1.288 [0.110, 15.133] | 0.8406 |

**S2.8.14 (crude)	mrc2 ~ time.factor + wave3 + (1|patient)**

| **Term** | **OR [95% CI]** | **P-val.** |
| --- | --- | --- |
| (Intercept) | 3.409 [0.593, 19.588] | 0.1691 |
| Time: 6 months | 0.696 [0.285, 1.701] | 0.4272 |
| Time: 12 months | 0.874 [0.349, 2.189] | 0.7741 |
| Wave: Second Wave | 2.582 [0.351, 19.002] | 0.3516 |
| Wave: Third wave | 0.174 [0.013, 2.420] | 0.1931 |

**S2.8.15 (cond)	mrc2 ~ time.factor + severity3 + wave3 + (1|patient)**

| **Term** | **OR [95% CI]** | **P-val.** |
| --- | --- | --- |
| (Intercept) | 0.889 [0.099, 7.968] | 0.9165 |
| Time: 6 months | 0.655 [0.260, 1.649] | 0.3685 |
| Time: 12 months | 0.784 [0.304, 2.020] | 0.6139 |
| Severity: Critical ARDS | 1.442 [0.144, 14.399] | 0.7552 |
| Severity: Critical Sepsis | 26.188 [2.199, 311.872] | **0.0098** |
| Wave: Second Wave | 3.693 [0.477, 28.600] | 0.2110 |
| Wave: Third wave | 0.471 [0.028, 7.961] | 0.6016 |

**S2.8.16 (crude)	mrc2 ~ time.factor + age3 + (1|patient)**

| **Term** | **OR [95% CI]** | **P-val.** |
| --- | --- | --- |
| (Intercept) | 0.784 [0.105, 5.851] | 0.8120 |
| Time: 6 months | 0.714 [0.299, 1.705] | 0.4485 |
| Time: 12 months | 0.990 [0.404, 2.424] | 0.9816 |
| Age: 40-60 yrs | 5.395 [0.475, 61.305] | 0.1741 |
| Age: >60 yrs | 6.412 [0.492, 83.540] | 0.1560 |

**S2.8.17 (cond)	mrc2 ~ time.factor + severity3 + age3 + (1|patient)**

| **Term** | **OR [95% CI]** | **P-val.** |
| --- | --- | --- |
| (Intercept) | 0.158 [0.011, 2.377] | 0.1823 |
| Time: 6 months | 0.675 [0.268, 1.704] | 0.4055 |
| Time: 12 months | 0.821 [0.318, 2.121] | 0.6838 |
| Severity: Critical ARDS | 1.945 [0.184, 20.598] | 0.5804 |
| Severity: Critical Sepsis | 48.804 [3.602, 661.280] | **0.0035** |
| Age: 40-60 yrs | 7.776 [0.468, 129.199] | 0.1526 |
| Age: >60 yrs | 17.650 [0.758, 410.773] | 0.0738 |

**S2.8.18 (crude)	mrc2 ~ time.factor + clusters2 + (1|patient)**

| **Term** | **OR [95% CI]** | **P-val.** |
| --- | --- | --- |
| (Intercept) | 2.120 [0.614, 7.316] | 0.2346 |
| Time: 6 months | 0.740 [0.311, 1.757] | 0.4943 |
| Time: 12 months | 1.012 [0.416, 2.463] | 0.9785 |
| Clustering Symptoms: Cluster 2 | 2.237 [0.476, 10.517] | 0.3080 |

**S2.8.19 (cond)	mrc2 ~ time.factor + severity3 + clusters2 + (1|patient)**

| **Term** | **OR [95% CI]** | **P-val.** |
| --- | --- | --- |
| (Intercept) | 1.299 [0.281, 6.012] | 0.7379 |
| Time: 6 months | 0.698 [0.282, 1.725] | 0.4359 |
| Time: 12 months | 0.873 [0.344, 2.214] | 0.7752 |
| Severity: Critical ARDS | 1.630 [0.186, 14.278] | 0.6588 |
| Severity: Critical Sepsis | 32.294 [2.519, 413.941] | **0.0076** |
| Clustering Symptoms: Cluster 2 | 0.959 [0.171, 5.373] | 0.9616 |

**S2.8.20 (crude)	mrc2 ~ time.factor + clusterc2 + (1|patient)**

| **Term** | **OR [95% CI]** | **P-val.** |
| --- | --- | --- |
| (Intercept) | 13.821 [1.253, 152.490] | **0.0320** |
| Time: 6 months | 0.727 [0.293, 1.802] | 0.4910 |
| Time: 12 months | 0.968 [0.382, 2.456] | 0.9461 |
| Clustering Comorbidities: Cluster 2 | 0.057 [0.003, 0.994] | **0.0496** |

**S2.8.21 (cond)	mrc2 ~ time.factor + severity3 + clusterc2 + (1|patient)**

| **Term** | **OR [95% CI]** | **P-val.** |
| --- | --- | --- |
| (Intercept) | 4.420 [0.780, 25.064] | 0.0932 |
| Time: 6 months | 0.696 [0.275, 1.760] | 0.4435 |
| Time: 12 months | 0.829 [0.321, 2.143] | 0.6988 |
| Severity: Critical ARDS | 1.401 [0.148, 13.298] | 0.7692 |
| Severity: Critical Sepsis | 32.245 [3.050, 340.898] | **0.0039** |
| Clustering Comorbidities: Cluster 2 | 0.058 [0.006, 0.540] | **0.0124** |

**S2.8.22 (comp) mrc2 ~ severity3+time.factor+sex2+obesity2+smoking2+dursymp2+ethnicity2+vaccination2+ wave3 + age3+clusters2+clusterc2+(1|patient)**

| **Term** | **OR [95% CI]** | **P-val.** |
| --- | --- | --- |
| (Intercept) | 0.002 [0.000, 2.191] | 0.0828 |
| Severity: Critical ARDS | 2.819 [0.188, 42.207] | 0.4528 |
| Severity: Critical Sepsis | 87.818 [1.511, 5102.447] | **0.0308** |
| Time: 6 months | 0.649 [0.233, 1.802] | 0.4062 |
| Time: 12 months | 0.743 [0.265, 2.085] | 0.5725 |
| Sex: Female | 99.796 [2.416, 4122.305] | **0.0153** |
| Obesity: Yes | 3.754 [0.324, 43.567] | 0.2902 |
| Smoking status: Smoke(d) | 0.977 [0.112, 8.564] | 0.9836 |
| Duration symptoms: More than 1 week | 5.851 [0.467, 73.392] | 0.1710 |
| Ethnicity: Non-White | 3.170 [0.207, 48.506] | 0.4072 |
| Vaccination: Yes | 1.056 [0.027, 42.060] | 0.9769 |
| Wave: Second Wave | 7.897 [0.451, 138.410] | 0.1573 |
| Wave: Third wave | 2.120 [0.037, 122.825] | 0.7167 |
| Age: 40-60 yrs | 4.915 [0.156, 155.010] | 0.3659 |
| Age: >60 yrs | 12.594 [0.180, 880.664] | 0.2424 |
| Clustering Symptoms: Cluster 2 | 4.613 [0.355, 59.965] | 0.2427 |
| Clustering Comorbidities: Cluster 2 | 0.128 [0.011, 1.478] | 0.0996 |

**S2.9 Mood disorder**

**S2.9.1 (sev)	depression2 ~ time.factor + severity3 + (1|patient)**

| **Term** | **OR [95% CI]** | **P-val.** |
| --- | --- | --- |
| (Intercept) | 1.068 [0.432, 2.644] | 0.8862 |
| Time: 6 months | 0.893 [0.410, 1.948] | 0.7766 |
| Time: 12 months | 1.433 [0.641, 3.202] | 0.3803 |
| Severity: Critical ARDS | 0.726 [0.174, 3.034] | 0.6611 |
| Severity: Critical Sepsis | 4.950 [1.166, 21.016] | **0.0302** |

**S2.9.2 (crude)	depression2 ~ time.factor + sex2 + (1|patient)**

| **Term** | **OR [95% CI]** | **P-val.** |
| --- | --- | --- |
| (Intercept) | 0.785 [0.355, 1.735] | 0.5495 |
| Time: 6 months | 0.919 [0.421, 2.009] | 0.8330 |
| Time: 12 months | 1.611 [0.722, 3.596] | 0.2442 |
| Sex: Female | 7.164 [1.817, 28.247] | **0.0049** |

**S2.9.3 (cond)	depression2 ~ time.factor + severity3 + sex2 + (1|patient)**

| **Term** | **OR [95% CI]** | **P-val.** |
| --- | --- | --- |
| (Intercept) | 0.415 [0.137, 1.259] | 0.1204 |
| Time: 6 months | 0.907 [0.408, 2.014] | 0.8105 |
| Time: 12 months | 1.454 [0.640, 3.302] | 0.3713 |
| Severity: Critical ARDS | 0.889 [0.202, 3.915] | 0.8760 |
| Severity: Critical Sepsis | 7.618 [1.593, 36.419] | **0.0110** |
| Sex: Female | 9.788 [2.192, 43.697] | **0.0028** |

**S2.9.4 (crude)	depression2 ~ time.factor + obesity2 + (1|patient)**

| **Term** | **OR [95% CI]** | **P-val.** |
| --- | --- | --- |
| (Intercept) | 0.818 [0.369, 1.809] | 0.6191 |
| Time: 6 months | 0.939 [0.428, 2.058] | 0.8750 |
| Time: 12 months | 1.553 [0.692, 3.485] | 0.2856 |
| Obesity: Yes | 7.632 [1.800, 32.356] | **0.0058** |

**S2.9.5 (cond)	depression2 ~ time.factor + severity3 + obesity2 + (1|patient)**

| **Term** | **OR [95% CI]** | **P-val.** |
| --- | --- | --- |
| (Intercept) | 0.670 [0.247, 1.816] | 0.4315 |
| Time: 6 months | 0.913 [0.413, 2.020] | 0.8219 |
| Time: 12 months | 1.445 [0.636, 3.279] | 0.3792 |
| Severity: Critical ARDS | 0.549 [0.123, 2.457] | 0.4328 |
| Severity: Critical Sepsis | 3.847 [0.886, 16.711] | 0.0722 |
| Obesity: Yes | 7.133 [1.630, 31.217] | **0.0091** |

**S2.9.6 (crude)	depression2 ~ time.factor + smoking2 + (1|patient)**

| **Term** | **OR [95% CI]** | **P-val.** |
| --- | --- | --- |
| (Intercept) | 1.721 [0.777, 3.815] | 0.1811 |
| Time: 6 months | 0.910 [0.421, 1.967] | 0.8102 |
| Time: 12 months | 1.562 [0.706, 3.456] | 0.2712 |
| Smoking status: Smoke(d) | 0.688 [0.207, 2.284] | 0.5415 |

**S2.9.7 (cond)	depression2 ~ time.factor + severity3 + smoking2 + (1|patient)**

| **Term** | **OR [95% CI]** | **P-val.** |
| --- | --- | --- |
| (Intercept) | 1.176 [0.441, 3.134] | 0.7454 |
| Time: 6 months | 0.895 [0.410, 1.953] | 0.7804 |
| Time: 12 months | 1.432 [0.641, 3.201] | 0.3815 |
| Severity: Critical ARDS | 0.741 [0.177, 3.108] | 0.6824 |
| Severity: Critical Sepsis | 4.942 [1.163, 21.003] | **0.0305** |
| Smoking status: Smoke(d) | 0.728 [0.215, 2.471] | 0.6109 |

**S2.9.8 (crude)	depression2 ~ time.factor + dursymp2 + (1|patient)**

| **Term** | **OR [95% CI]** | **P-val.** |
| --- | --- | --- |
| (Intercept) | 1.920 [0.697, 5.286] | 0.2070 |
| Time: 6 months | 0.901 [0.416, 1.953] | 0.7924 |
| Time: 12 months | 1.566 [0.706, 3.471] | 0.2698 |
| Duration symptoms: More than 1 week | 0.692 [0.216, 2.218] | 0.5358 |

**S2.9.9 (cond)	depression2 ~ time.factor + severity3 + dursymp2 + (1|patient)**

| **Term** | **OR [95% CI]** | **P-val.** |
| --- | --- | --- |
| (Intercept) | 1.443 [0.452, 4.612] | 0.5359 |
| Time: 6 months | 0.884 [0.404, 1.933] | 0.7569 |
| Time: 12 months | 1.431 [0.639, 3.207] | 0.3834 |
| Severity: Critical ARDS | 0.715 [0.168, 3.036] | 0.6494 |
| Severity: Critical Sepsis | 5.210 [1.201, 22.605] | **0.0275** |
| Duration symptoms: More than 1 week | 0.604 [0.183, 1.993] | 0.4081 |

**S2.9.10 (crude)	depression2 ~ time.factor + ethnicity2 + (1|patient)**

| **Term** | **OR [95% CI]** | **P-val.** |
| --- | --- | --- |
| (Intercept) | 1.257 [0.597, 2.649] | 0.5470 |
| Time: 6 months | 0.910 [0.419, 1.974] | 0.8111 |
| Time: 12 months | 1.569 [0.707, 3.482] | 0.2685 |
| Ethnicity: Non-White | 2.404 [0.602, 9.606] | 0.2145 |

**S2.9.11 (cond) depression2 ~ time.factor + severity3 + ethnicity2 + (1|patient)**

| **Term** | **OR [95% CI]** | **P-val.** |
| --- | --- | --- |
| (Intercept) | 0.968 [0.378, 2.480] | 0.9458 |
| Time: 6 months | 0.894 [0.409, 1.954] | 0.7792 |
| Time: 12 months | 1.445 [0.645, 3.235] | 0.3706 |
| Severity: Critical ARDS | 0.709 [0.168, 2.993] | 0.6397 |
| Severity: Critical Sepsis | 4.502 [1.047, 19.353] | **0.0432** |
| Ethnicity: Non-White | 1.798 [0.445, 7.253] | 0.4100 |

**S2.9.12 (crude)	depression2 ~ time.factor + vaccination2 + (1|patient)**

| **Term** | **OR [95% CI]** | **P-val.** |
| --- | --- | --- |
| (Intercept) | 1.746 [0.824, 3.702] | 0.1458 |
| Time: 6 months | 0.908 [0.419, 1.968] | 0.8068 |
| Time: 12 months | 1.499 [0.673, 3.338] | 0.3214 |
| Vaccination: Yes | 0.435 [0.082, 2.309] | 0.3286 |

**S2.9.13 (cond) depression2 ~ time.factor + severity3 + vaccination2 + (1|patient)**

| **Term** | **OR [95% CI]** | **P-val.** |
| --- | --- | --- |
| (Intercept) | 1.229 [0.454, 3.323] | 0.6849 |
| Time: 6 months | 0.891 [0.408, 1.947] | 0.7730 |
| Time: 12 months | 1.397 [0.623, 3.135] | 0.4170 |
| Severity: Critical ARDS | 0.669 [0.156, 2.872] | 0.5885 |
| Severity: Critical Sepsis | 4.535 [1.053, 19.536] | **0.0425** |
| Vaccination: Yes | 0.548 [0.099, 3.043] | 0.4913 |

**S2.9.14 (crude)	depression2 ~ time.factor + wave3 + (1|patient)**

| **Term** | **OR [95% CI]** | **P-val.** |
| --- | --- | --- |
| (Intercept) | 2.209 [0.669, 7.294] | 0.1936 |
| Time: 6 months | 0.925 [0.421, 2.030] | 0.8453 |
| Time: 12 months | 1.337 [0.593, 3.014] | 0.4835 |
| Wave: Second Wave | 1.068 [0.271, 4.208] | 0.9249 |
| Wave: Third wave | 0.204 [0.034, 1.213] | 0.0805 |

**S2.9.15 (cond)	depression2 ~ time.factor + severity3 + wave3 + (1|patient)**

| **Term** | **OR [95% CI]** | **P-val.** |
| --- | --- | --- |
| (Intercept) | 1.686 [0.369, 7.702] | 0.5005 |
| Time: 6 months | 0.895 [0.406, 1.976] | 0.7844 |
| Time: 12 months | 1.278 [0.565, 2.895] | 0.5559 |
| Severity: Critical ARDS | 0.532 [0.115, 2.457] | 0.4191 |
| Severity: Critical Sepsis | 3.454 [0.769, 15.514] | 0.1059 |
| Wave: Second Wave | 1.120 [0.274, 4.567] | 0.8749 |
| Wave: Third wave | 0.264 [0.040, 1.747] | 0.1674 |

**S2.9.16 (crude)	depression2 ~ time.factor + age3 + (1|patient)**

| **Term** | **OR [95% CI]** | **P-val.** |
| --- | --- | --- |
| (Intercept) | 2.709 [0.607, 12.090] | 0.1915 |
| Time: 6 months | 0.910 [0.420, 1.971] | 0.8110 |
| Time: 12 months | 1.582 [0.712, 3.513] | 0.2600 |
| Age: 40-60 yrs | 0.620 [0.122, 3.144] | 0.5636 |
| Age: >60 yrs | 0.366 [0.063, 2.133] | 0.2640 |

**S2.9.17 (cond)	depression2 ~ time.factor + severity3 + age3 + (1|patient)**

| **Term** | **OR [95% CI]** | **P-val.** |
| --- | --- | --- |
| (Intercept) | 1.764 [0.354, 8.789] | 0.4887 |
| Time: 6 months | 0.894 [0.410, 1.951] | 0.7782 |
| Time: 12 months | 1.455 [0.650, 3.258] | 0.3619 |
| Severity: Critical ARDS | 0.686 [0.159, 2.951] | 0.6123 |
| Severity: Critical Sepsis | 4.488 [1.040, 19.367] | **0.0442** |
| Age: 40-60 yrs | 0.661 [0.128, 3.407] | 0.6211 |
| Age: >60 yrs | 0.464 [0.079, 2.740] | 0.3969 |

**S2.9.18 (crude)	depression2 ~ time.factor + clusters2 + (1|patient)**

| **Term** | **OR [95% CI]** | **P-val.** |
| --- | --- | --- |
| (Intercept) | 1.003 [0.406, 2.479] | 0.9955 |
| Time: 6 months | 0.936 [0.431, 2.034] | 0.8674 |
| Time: 12 months | 1.527 [0.687, 3.397] | 0.2990 |
| Clustering Symptoms: Cluster 2 | 2.240 [0.686, 7.318] | 0.1817 |

**S2.9.19 (cond) depression2 ~ time.factor + severity3 + clusters2 + (1|patient)**

| **Term** | **OR [95% CI]** | **P-val.** |
| --- | --- | --- |
| (Intercept) | 0.911 [0.321, 2.586] | 0.8617 |
| Time: 6 months | 0.906 [0.415, 1.981] | 0.8048 |
| Time: 12 months | 1.427 [0.638, 3.193] | 0.3863 |
| Severity: Critical ARDS | 0.710 [0.168, 2.997] | 0.6416 |
| Severity: Critical Sepsis | 4.354 [0.981, 19.328] | 0.0531 |
| Clustering Symptoms: Cluster 2 | 1.458 [0.432, 4.922] | 0.5433 |

**S2.9.20 (crude)	depression2 ~ time.factor + clusterc2 + (1|patient)**

| **Term** | **OR [95% CI]** | **P-val.** |
| --- | --- | --- |
| (Intercept) | 3.124 [1.198, 8.147] | **0.0198** |
| Time: 6 months | 0.924 [0.424, 2.016] | 0.8431 |
| Time: 12 months | 1.537 [0.688, 3.435] | 0.2948 |
| Clustering Comorbidities: Cluster 2 | 0.201 [0.055, 0.733] | **0.0151** |

**S2.9.21 (cond) depression2 ~ time.factor + severity3 + clusterc2 + (1|patient)**

| **Term** | **OR [95% CI]** | **P-val.** |
| --- | --- | --- |
| (Intercept) | 2.253 [0.749, 6.776] | 0.1483 |
| Time: 6 months | 0.907 [0.413, 1.996] | 0.8093 |
| Time: 12 months | 1.402 [0.622, 3.164] | 0.4153 |
| Severity: Critical ARDS | 0.675 [0.156, 2.913] | 0.5983 |
| Severity: Critical Sepsis | 5.235 [1.206, 22.715] | **0.0271** |
| Clustering Comorbidities: Cluster 2 | 0.187 [0.050, 0.693] | **0.0121** |

**S2.9.22 (comp) depression2 ~ severity3+time.factor+sex2+obesity2+smoking2+dursymp2+ethnicity2+vaccination2+ wave3 + age3+clusters2+clusterc2+(1|patient)**

| **Term** | **OR [95% CI]** | **P-val.** |
| --- | --- | --- |
| (Intercept) | 2.931 [0.111, 77.714] | 0.5202 |
| Severity: Critical ARDS | 0.449 [0.081, 2.477] | 0.3580 |
| Severity: Critical Sepsis | 2.915 [0.527, 16.116] | 0.2202 |
| Time: 6 months | 0.976 [0.424, 2.245] | 0.9546 |
| Time: 12 months | 1.336 [0.567, 3.149] | 0.5081 |
| Sex: Female | 8.300 [1.726, 39.921] | **0.0083** |
| Obesity: Yes | 3.231 [0.664, 15.718] | 0.1462 |
| Smoking status: Smoke(d) | 1.085 [0.277, 4.252] | 0.9067 |
| Duration symptoms: More than 1 week | 0.888 [0.223, 3.541] | 0.8666 |
| Ethnicity: Non-White | 1.763 [0.359, 8.668] | 0.4851 |
| Vaccination: Yes | 1.139 [0.118, 11.014] | 0.9104 |
| Wave: Second Wave | 0.956 [0.176, 5.200] | 0.9589 |
| Wave: Third wave | 0.206 [0.016, 2.613] | 0.2230 |
| Age: 40-60 yrs | 0.307 [0.043, 2.191] | 0.2389 |
| Age: >60 yrs | 0.200 [0.021, 1.915] | 0.1627 |
| Clustering Symptoms: Cluster 2 | 1.658 [0.391, 7.025] | 0.4926 |
| Clustering Comorbidities: Cluster 2 | 0.231 [0.051, 1.047] | 0.0573 |
